# Inhibition of CD38 enzyme activity on engrafted human immune cells enhances NAD+ metabolism and inhibits inflammation in an *in-vivo* model of xeno-GvHD

**DOI:** 10.3389/fimmu.2025.1640611

**Published:** 2025-10-13

**Authors:** Ghenima Ahmil-Boiteau, Pranjali Dalvi, Kevin Dang, Harshad S. Ugamraj, Giulia Castello, James Allison, Ute Schellenberger, Roland Buelow, Suhasini Iyer, Ben Buelow, Eduardo Chini, Bruce Blazar, Maria Cristina Cuturi, Wim van Schooten, Laure-Hélène Ouisse

**Affiliations:** ^1^ INSERM, Nantes Université, CHU Nantes, Center for Research in Transplantation and Translational Immunology (CR2TI), UMR 1064, Nantes, France; ^2^ Teneobio, Inc., Menlo Park, CA, United States; ^3^ Department of Anesthesiology and Perioperative Medicine, Mayo Clinic, Jacksonville, FL, United States; ^4^ Masonic Cancer Center, and Division of Blood and Marrow Transplantation, Department of Pediatrics, University of Minnesota, Minneapolis, MN, United States

**Keywords:** CD38, GvHD, NAD+, inflammation, TNB-738

## Abstract

**Introduction:**

CD38 is highly expressed on immune cells. It catabolizes NAD+, which is a critical cofactor for enzymes involved in metabolism and energy production.

**Methods and results:**

We developed TNB-738, a fully human antibody that potently inhibits human CD38 enzymatic activity on human immune cells, resulting in a dose-dependent increase of intracellular NAD+ levels. TNB-738 does not show immune effector functions, does not induce direct cell killing of CD38 positive cells and is not internalized. In vivo, treatment with TNB-738 following infusion of human PBMCs into NSG mice resulted in significantly lower clinical scores, prolonged overall survival, less expansion of engrafted human CD45+ cells and a significant expansion of Tregs.

**Discussion:**

CD38 positive T cells regulate NAD+ metabolism in inflamed tissues and blockade of CD38 enzyme activity by TNB-738 could represent a novel class of therapeutics for the treatment of inflammatory conditions, including GvHD.

## Introduction

CD38 is a multifunctional ectoenzyme expressed predominantly on immune cells and is upregulated in response to stimulation by cytokines, endotoxin, and other inflammatory insults (1). Its primary role is to catabolize NAD+ and its precursor NMN. Activation of immune cells thus upregulates extracellular NADase and NMNase enzymatic activities, which leads to substantial declines of NAD+ and NMN within CD38 positive immune cells and CD38 negative cells of surrounding tissues (1). Since CD38 expression is known to increase with age and inflammation (1), inhibition of CD38 enzymatic activity is emerging as a therapeutic approach to boost cellular NAD+ levels for the treatment of diseases associated with aging, fibrosis, and inflammation (2, 3).

NAD+ is a cofactor for enzymes critical in cellular energy pathways, DNA repair, gene regulation, and protein homeostasis (4). Several enzymes in the Krebs cycle and glycolysis are NAD+ dependent and, hence, bioavailability of NAD+ regulates these pathways. Energy metabolism of T cells changes as they are activated and start to differentiate. Activation of T cells is initiated through the interaction of the T cell receptor with its cognate MHC-peptide complex on antigen presenting cells (APC), followed by additional stimulation via soluble cytokines and transmembrane costimulatory molecules on APCs. One of the downstream effects is that glucose uptake increases, and glycolysis is upregulated. Intervening in glycolysis with antagonists can both disrupt differentiation of naïve cells into memory T cells (T_mem_) and prevent activation of established T_mem_ cells. Mitochondria play a critical role in energy metabolism and many studies have shown that increases in intracellular NAD+ lead to increased mitochondrial respiration. Moreover, various NAD+ dependent enzymes regulate epigenetic changes and post-translational modifications of proteins. Examples of such enzymes are Sirtuins and PARPs, which are known to play a role in cell activation and differentiation. NAD+ has pleiotropic effects on many intracellular pathways and increasing NAD+ in immune cells and tissues could be beneficial in many disease indications (2).

Inhibition of CD38 with either small chemical compounds or antibodies results in less inflammation and higher intracellular NAD+ concentrations in aged mice (1). As animals age, NAD+ levels decrease, which augments the chronic inflammation characteristic of aged tissues, also called “Inflammaging”. Myeloid-derived cells and T cells infiltrate tissues in response to senescence-associated secretory phenotype (SASP) cytokines produced by local senescent cells. These infiltrating immune cells highly express CD38 (1). Similarly, in a bleomycin induced injury model, inhibition of CD38 in mice resulted in reduced inflammation and fibrosis of the lung and skin (3). We present in this paper that inhibition of the enzyme activity of CD38 on human T cells protects NSG mice against Graft-versus-Host Disease (GvHD) while leaving the Graft-versus-Tumor (GvT) response intact.

GvHD is a life-threatening complication of allogeneic hematopoietic stem cell transplants (allo-HSCT) driven by alloreactive donor T cells. Routinely, patients are treated with immunosuppressive therapy (IST) prophylactically to prevent GvHD. Approximately 30-70% of patients on standard IST develop chronic or acute GvHD (5) (6). Acute GvHD typically occurs within 3–6 months after transplantation. Chronic GvHD is predominantly a fibroproliferative disorder that can affect any tissue or organ system in the body. Both forms of GvHD are difficult to treat, steroids being first line treatments and further treatments are needed (7–11) Complications of IST include increased risk of infections, organ toxicities, and recurrent malignancies. Ideally, treatments should target the underlying multi-organ inflammation caused by the graft but leave the anti-tumor immune responses intact. Infusion of human immune cells into immune-comprised NOD SCID gamma (NSG) mice results in a xenogeneic GVHD reaction with severe inflammation and, ultimately, lethal tissue damage and serves as a preclinical model system in which to test anti-inflammatory therapies targeting T cells (12, 13).

TNB-738, a fully human antibody with a silenced IgG4, potently inhibits human CD38 enzymatic activity on human immune cells, resulting in a dose dependent increase of intracellular NAD+ levels as well as a downstream increase of sirtuin 1 expression and activity *in vitro* and *in vivo* (5). Sirtuins are NAD-dependent histone deacetylases present in different compartments of the cell and have been shown to regulate T cell memory development and other aspects of immunity (6). TNB-738 does not show *in vitro* effector functions, does not induce direct cell killing of CD38 positive cells nor removal of CD38 from the membrane by internalization, supporting inhibition of membrane CD38 enzymatic activity as the mechanism of action (5, 6). Interestingly, the use of daratumumab, an FDA approved monoclonal antibody against CD38, in a cohort of 34 multiple myeloma patients after allo-HSCT resulted in low aGvHD (15%) and no cGvHD, suggesting that CD38 is an attractive target for the treatment of GvHD (7). However, daratumumab depletes many immune cells, including T cells, NK cells and monocytes, which could explain the beneficial effects of daratumumab in allo-HSCT on top of the removal of CD38 by internalization. We hypothesize that TNB-738 would be a safer and more effective alternative, as the GvT activity of the allograft would be preserved.

*In vivo*, treatment of human T cell engrafted NSG mice with TNB-738 resulted in increased NAD+ levels and sirtuin 1 activity in the engrafted T cells and surrounding murine tissues. This indicates that NAD+ metabolism of both infiltrating human T cells and surrounding tissues is modified by the inhibition of human CD38 on engrafted immune cells. We conclude this for two reasons, namely (1); TNB-738 is specific for human CD38 and does not bind mouse CD38, and (2) antibody Ab68, which is specific for mouse CD38, had no effect on GvHD. Treatment with TNB-738 for 18 days following infusion of human peripheral blood mononuclear cells (PBMC) into NSG mice resulted in significantly lower clinical scores and prolonged survival. Treatment with TNB-738 led to less overall expansion of engrafted human CD45+ cells, but a significant expansion of Tregs compared to vehicle treated animals. Spleen and bone marrow of treated animals contained human T cells in significant numbers, which were fully responsive to stimuli in *ex vivo* experiments. Importantly, TNB-738 treatment did not reduce the anti-tumor response of the grafted human T cells (GvT) in humanized NSG mice versus mice treated with vehicle. RNA sequencing of human CD45 (huCD45) cells purified from the blood of xeno-GvHD mice treated with TNB-738 at day 11 after transplantation showed significantly increased expression of genes associated with mitochondrial homeostasis and decreased expression of T cell activating. Untargeted metabolomics of the liver tissue from these mice showed decreased readouts of oxidative stress, inflammation, and tissue turnover indicating that TNB-738 improved overall health compared. In conclusion, CD38 positive T cells regulate NAD+ metabolism in inflamed tissues and blockade of the enzymatic function of CD38 by TNB-738 could represent a novel class of therapeutics for the treatment of inflammatory conditions, including GvHD.

## Materials and methods

### NAD+ concentration

Frozen spleen and liver tissues from the xeno-GvHD mice were homogenized in 1X Extraction Buffer provided with the Cell Biolab NAD+ assay kit (San Diego, CA). Tissue homogenates were centrifuged 14000 rpm for 5 minutes at 4^∘^C. Supernatants were retrieved, and NAD+ was extracted and measured using Cell Biolab NAD+ assay kit according to manufacturer’s instructions. Briefly, 0.1 N HCl was added to the supernatants and incubated at 80^∘^C for an hour protected from light then neutralized by adding the assay buffer. An NAD+ standard linear regression plot was made using provided NAD+ standard solution. Equal volumes of NAD+ cycling reagent was added with either the standard or sample and incubated for 3 hours at room temperature protected from light. Following incubation, absorbance was measured at 450 nm.

### Sirtuin1 activity

Frozen spleen and liver tissues from the xeno-GvHD mice were homogenized in 1X M-PER™ Mammalian Protein Extraction Reagent (ThermoFisher Scientific) followed by centrifugation at 14000 rpm for 5 minutes at 4^∘^C. Supernatants were retrieved, and protein concentration was measured using BCA reagent (ThermoFisher). Equal amount of protein across both treated and untreated groups was incubated with 1:30 of the anti-SIRT1 antibody (Abcam, Cambridge, MA) for 4 hours on a rotator at 4^∘^C. Protein A slurry (Thermofisher) was added, and samples were further incubated for 2 hours at room temperature followed by addition of immunoprecipitation buffer (IP) and centrifugation at 2500 g for 2 minutes. The wash steps with IP buffer were repeated. IgG elution buffer was added for 5 minutes, after which the eluate was centrifuged at 2500 g for 2 minutes. The supernatant was neutralized with 1 M Tris (pH 9) at 1:10. The SIRT Glo assay was performed on the elute according to manufacturer’s instructions (Promega, Madison, WI). Briefly, the SIRT-Glo reagent was made by mixing the SIRT-Glo buffer, SIRT-Glo substrate, and the developer reagent. Equal volume of SIRT-Glo reagent and sample was added to each well and was shaken at 500 rpm for 10 seconds. The plate was incubated at room temperature for 30 minutes. Following incubation, relative luminescence units (RLU) were measured.

### Blood collection and PBMC separation

Blood was collected at the Etablissement Français du Sang (Nantes, France) from healthy donors. Written informed consent was provided according to institutional guidelines. PBMCs were isolated by Ficoll–Paque density-gradient centrifugation (Eurobio, Courtaboeuf, France). Remaining red blood cells and platelets were eliminated with a hypotonic solution. The cells were then washed, counted, and used fresh or frozen for later use. Fresh and frozen PMBCs caused identical survival outcomes, clinical scores and weight loss in our xeno-GvHD model in NSg mice.

### Xeno-graft versus host disease mouse model

NOD/SCID/IL2Rγ−/− (NSG) purchased from Charles River were irradiated with 1.5 Gy and injected intravenously (*i.v*) the day after with 15.10^6^ fresh PBMCs or 20.10^6^ thawed isolated PBMCs in a final volume of 200 µl PBS. 100 or 130 µg of TNB-738 (anti-human CD38), 100µg of Ab68 (anti-mouse CD38) or PBS (control group) was injected intraperitoneally (*i.p*) twice a week from day 0 until day 18 or until euthanasia. Humanized NSG mice were euthanized when percentage of weight loss was >20% of their initial weight for the survival study.

The clinical score of the animals was noted using a point system established in the form of a grid. The clinical score combines the percentage of weight loss, activity, posture, and fur texture of each animal (8). Each criterion was evaluated on a scale of 0-3 (**Table 1**).

**Table 1:** Clinical score.

Parameters	0 point	1 point	2 points	3 points	4 points
Weight loss	<5%	5-10%	11-15%	15-20%	>20%
Posture	Normal	Hunching noted only at rest	Severe hunching impairs movement		
Activity	Normal	Mild decreased	Moderately decreased	Stationary unless stimulated	
Fur texture	Normal	Mild decreased	Moderately decreased	Severe ruffling/poor grooming	

### Graft versus tumor model in NSG mice7

A total of 5.10^6^ MDA-MB-231 breast cancer cells were injected subcutaneously in 100 μL of PBS into the flank of male NSG mice (7 to 12 weeks old). When the tumor was detected, 10.10^6^ PBMCs from a HV donor were injected i.v (day 0). Transplanted mice received anti-human CD38 mAb (TNB-738) twice a week at 130 µg per injection intraperitoneally or PBS starting from day 0 until day 18. Body weight and tumor size were measured at least twice a week for 50 days, and mice were euthanized when percentage of weight loss was ≥20% of their initial weight or when the tumor size reached 3000 mm^3^. Tumor volume was calculated using the following formula: V = π/6 × f(length × width)^3/2^; females: f = H/LW = 1.58 ± 0.01; males: f = H/LW = 1.69 ± 0.03 (9, 10),

### Analysis of immune cells in spleen and bone marrow

Post-mortem, the spleens and femurs from NSG humanized mice were collected for immune cell analysis. Spleens were minced and dissolved in PBS. To extract the bone marrow, the femurs were cut at both ends (proximal and distal), and the inside of the femurs were rinsed with PBS using a syringe and needle. The cellular suspension was filtered through a 100 µm cell strainer to isolate splenocytes and bone marrow cells (BM). Red blood cells and platelets were eliminated with a hypotonic solution and centrifugation. Cells were washed, re-suspended in PBS-FCS-2%-1mM EDTA, and counted. Single-cell suspensions were stained with viability dye eFluor450 (eBiosciences) and with anti-human CD45 APC H7 (BD Biosciences), anti-humanCD19 APC (BD Biosciences), anti-humanCD69 APC (Miltenyi), Viability Dye eFluor 500 or Viability Dye eFluor 450 (eBiosciences), anti-human CD4 PercpCy5.5 (BD Biosciences), CD3 Pecy7 (BD Biosciences), Anti-humanFoxP3 PE (eBiosciences), CD3 BV510 (BD Biosciences), anti-human CD4 PeCy7 (BD Biosciences), anti-human CD16 FITC (BD Biosciences), and anti-human CD14 PE (BD Biosciences). For intracellular staining, cells were fixed and permeabilized with Fix/Perm kit (eBiosciences), and intracellular staining was performed in Permeabilization buffer. Analysis was performed on a BD FACS Verse with FACSuite Software version 1.0.6. Post-acquisition analysis was performed using FlowJo software.

### Histological analysis

Liver and colon were sampled, fixed in 10% neutral buffered formalin, and further embedded in paraffin wax. 5-μm-thick sections were then stained with routine hematoxylin-eosin-saffron (HES) for histopathological evaluation by a veterinary pathologist blind to treatment. GvHD assessment was performed using a semiquantitative scoring system (0.5 to 4.0 grades) as previously published (11) (**Tables 2**, **3**).

**Table 2:** Histological scoring system of liver.

Grade	Histological characteristics
0	Normal
0, 5	Minimal perivascular portal cuffing, 1–2 cells in thickness
1	Perivascular cuffing, 1 to 2 cells in thickness, involving up to 15% of vessels, 1–2 cells in thickness, involving up to 15% of vessels
1, 5	Previous feature with additional infiltration into parenchyma
2	Perivascular cuffing, 2 to 3 cells in thickness, involving up to 25% of vessels and infiltration into parenchyma
2, 5	Perivascular cuffing, 2 to 3 cells in thickness, involving 25% to 50% of vessels and infiltration into parenchyma
3	Perivascular cuffing, 4 to 5 cells in thickness, involving 25% to 50% of vessels, isolated hepatocytic necrosis and infiltration into parenchyma proper
3, 5	Perivascular cuffing, 6 to 7 cells in thickness, involving greater than 50% of vessels, necrotic foci and infiltration into parenchyma proper with severe disruption of structure
4	Perivascular cuffing, greater than 8 cells in thickness, involving greater than 50% of vessels, large necrotic foci and infiltration into parenchyma

**Table 3:** Histological scoring system of colon.

Grade	Histological characteristics
0	Normal
0, 5	Minimal infiltration in lamina propria
1	Minor infiltration of up to 20% of lamina propria, 1 to 2 cell thickness in intermucosal areas
1, 5	Minor infiltration of less than or equal to one third of the lamina propria, 1 to 2 cell thickness in intermucosal areas
2	Necrotic cells in less than 25% of crypts, infiltration of less than or equal to one third of the lamina propria, 3 cell thickness in intermucosal areas
2, 5	Necrotic cells in 25% to 50% of crypts, infiltration of less than or equal to one third of lamina propria, 3 to 4 cell thickness in intermucosal areas
3	Necrotic cells in greater than 50% of crypts, infiltration of lamina propria (5 to 6 cell thickness in intermucosal areas with loss of >25% of goblet cells
3, 5	Necrotic cells in greater than 50% of crypts, infiltration of lamina propria resulting in displacement of >50% of mucosa with loss of 50% of goblet cells
4	Necrotic cells in greater than 50% of crypts, infiltration of lamina propria resulting in displacement of greater than 50% of mucosa with loss of 75% to 100% of goblet cells

### Immunohistochemistry analysis

4-µm-thick sections were produced from formalin-fixed paraffin-embedded tissue samples. After dewaxing using grading alcohol, a demasking step was performed with incubation in citrate buffer (pH=6, Dako) at 98°C for 40 minutes and endogen peroxydases were further blocked using H2O2 (3%) for 10 minutes at room temperature (RT). Protein saturation was released with goat serum (5% in PBS) before incubation with primary rabbit polyclonal antibody against CD3 (1:150, Dako) for 1 hour at 37°C. After washing, a secondary anti-rabbit antibody was used (1:300, Dako) for 1 hour at RT. Antibody complexes were then revealed using OPAL 540 reagent (1:100, Akoya biosciences, Marlborough, MA) for 10 minutes. After removal of any unbound fluor using citrate buffer (pH=6, Dako) at 98°C for 20 minutes, slides were rinsed before nuclei counterstaining with DAPI for 15 minutes at RT and mounting with Mowiol medium. Digitization of whole slides was performed using fluorescence detection (Zeiss Axioscan Z1, x10 magnification).

### Multiplex immunoassays

To determine the cytokine concentration in serum, analysis was carried out according to the manufacturer instructions using the Ella Simple Plex system (Protein Simple kit, Biotechne SAS, Rennes, France) for IL-1rα, IL-1b, IL-2, IL-4, IL-6, IL-10, IL12p70, IL17A, IFNɣ, TNFα, or using a LuminexMagpix-based assay (Procartaplex kit, ThermoFisher Scientific, Courtabœuf, France) for IL-1β, IL-2, IL-6, IL-10, IL17A, IFNɣ, TNFα.

### Purification of human T cells for injection into NSG mice

T cells were purified from hPBMC with a Pan T cell negative selection kit from Miltenyi Biotec. Purified T cells were resuspended at 10.10^6^ in 200 µl of PBS and injected (*i.v*) in irradiated NSG mice.

### Preparation of CD25 negative T cells and injection into NSG mice

CD25 high cells were depleted from hPBMCs using CD25 MicroBeads II, human (Miltenyi Biotec) according to manufacturer’s instructions, and were separated using an autoMACS Pro Separator (Miltenyi Biotec). CD25^-^ cells were resuspended at 20.10^6^ in 200 µl of PBS and injected (*i.v*) in irradiated NSG mice.

### Proliferation of human cells in NSG mice

hPBMC were stained with CPD eFluor 450 (eBiosciences) according to the manufacturer’s instructions. Fluorescent dye-labelled PBMCs were adoptively transferred into the lateral tail vein of host irradiated NSG mice (20.10^6^ in 200 µl of PBS with insulin syringe). Mice were treated with TNB-738 (130 µg per mouse) twice a week starting on the day of injection of PBMCs (D0). After 4, 7 days, spleens of humanized mice were harvested, and splenocytes were isolated (see above, “Analysis of immune cells in spleen and bone marrow”) for staining and analysis by flow cytometry. Sera were collected for cytokine analysis.

### Anergy test

hPBMC were isolated from the spleen of treated animals by depleting mouse CD45 cells with mCD45 microbeads (Miltenyi) according to the manufacturer’s instructions. hCD45 were then stained with CPD450 (eBiosciences) and cultured with IL-2 (100 U/mL) for 6 days. Cells were then collected, stained with anti-human CD3 PeCy7 (BD biosciences), anti-human CD4 PercPCy5.5 (BD biosciences), and Viability Dye eFluor 506 (eBiosciences), and analyzed with a FACS Verse cytometer with FACSuite Software version 1.0.6; post-acquisition analysis were performed using FlowJo software.

### *Ex-vivo* xeno MLR

hPBMC were stained with CPD eFluor 450 (eBiosciences) according to the manufacturer’s instructions. NSG spleens were harvested, perfused with collagenase D, minced, and incubated for 20 min at 37°C. Spleen fragments were suspended in PBS-FCS 2%-1 mM EDTA and then forced through a mesh filter. A hypotonic solution and low centrifugation were used to remove remaining red cells and platelets. Cells were washed, re-suspended in PBS-FCS-2%-1 mM EDTA, and counted. hPBMC were co-cultured with NSG splenocytes for 7 days at a ratio of 1:1 in AIM V Serum Free Medium (Gibco) in the presence or absence of TNB-738 at a final concentration of 10 µg/mL. Cells were then collected, stained with anti-human CD3 PeCy7 (BD biosciences), anti-human CD4 PercPCy5.5 (BD biosciences), anti-human CD25 APCCy7 (BD biosciences), anti-human CD127 PE (BD biosciences), anti-human CD69 APC (Myltenyi Biotec), and Viability Dye eFluor 506 (eBiosciences). Cells were analyzed with a FACS Verse cytometer with FACSuite Software version 1.0.6; post-acquisition analysis were performed using FlowJo software.

### Untargeted metabolomics

To determine if TNB-738 treatment causes significant changes in the levels of known and novel metabolites in the GvHD induced mouse liver, untargeted metabolomics was performed at Metabolon, Morrisville NC. Harvested mouse livers on day 11 of treatment with TNB-738 or PBS were used, since this is the first timepoint after treatment where the first clinical signs of GvHD were observed. Liver tissue was extracted with methanol and divided into five fractions: two for analysis by two reverse phase (RP)/UPLC-MS/MS methods using positive ion mode electrospray ionization (ESI), one for analysis by RP/UPLC-MS/MS using negative ion mode ESI, and one reserved for back-up. Metabolyzer software was used to match ions to an in-house library of standards for the identification of metabolites and their quantification by peak area integration. The results were presented as concentrations of metabolites and statistical significance was determined by Welch’s two-sample t-test.

### Statistical analysis

Two-way repeated measure ANOVA was used to analyze mouse weight loss, clinical score, and tumor volume. Log Rank (Mantel Cox) test was used to analyze survival. Unpaired t test was used to compare cell immunophenotyping between two groups: untreated and treated (TNB-738).

## Results

### TNB-738 is a bispecific antibody that inhibits CD38 enzymatic activity without depleting CD38-expressing cells

**Figure 1:**
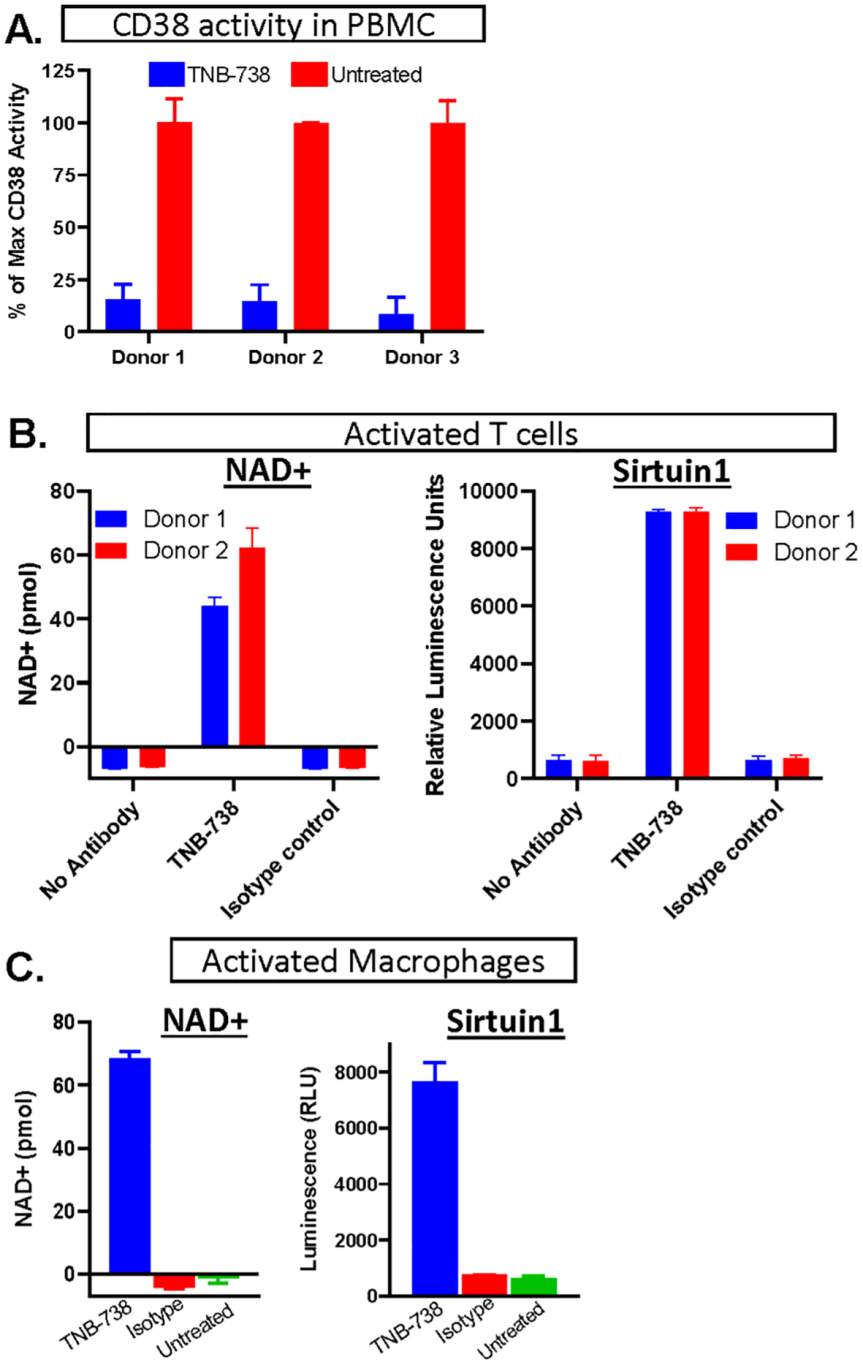
Inhibition of CD38 leads to increased NAD+ concentrations and higher NAD+-dependent enzyme activities. **(A)** hPBMC of three donors were treated (in blue) with TNB738 or vehicle (in red) and CD38 activity was measured as described in PMID: 35867844. Histograms represent percentage of maximal CD38 activity. **(B)** PBMCs derived from 2 different healthy volunteers were activated for 3 days in 2 independent experiments by adding anti-CD3 (OKT3) and IL-2, with or without TNB-738 or isotype control. On the third day, additional IL-2 was added. NAD+ and sirtuin1 activity measurement by SIRTGlo assay were performed the next day. **(C)** Monocytes (CD14+) isolated from hPBMC derived from 2 different healthy volunteers were activated for 6 days in 2 independent experiments by IFN-y, G-MCSF and LPS for M1 conversion and IL-4, M-CSF for M2 conversion. M1 and M2 macrophages expressed high levels of CD38 and hydrolase was assessed in the presence of TNB-738 or isotype control using the NAD+ assay and a sirtuin1 activity assay using by SIRTGlo.

TNB-738 is a fully human bispecific antibody that pairs heavy chain only UniAbs CD38_F11A and CD38_F12A on a silenced IgG4 Fc using a knobs-into-holes technology generated using rats humanized for their immunoglobulin genes (5, 12). Monospecific UniAbs, CD38_F11A and CD38_F12A, are partial inhibitors of human CD38, inhibiting hydrolase activity at approximately 50%. In combination, either as two individual UniAbs or paired in a biparatopic format (TNB-738), the antibodies synergize to inhibit approximately 90% hydrolase activities *in vitro* on PBMC (**Figure 1A**) (5). The immediate downstream effect of inhibiting CD38 enzyme activities is that the catabolism of NAD+ and NAD+ precursors, such as NMN, is blocked. As a consequence, intracellular NAD+ levels increased in CD38+ primary immune cells as well as activities of NAD-dependent enzymes, such as Sirtuin 1 (**Figures 1B, C**). Importantly, TNB-738 has a silenced IgG4 Fc tail, which abrogates all Fc-mediated immune functions, such as direct apoptosis, ADCC, and CDC of CD38 positive cells (5). In contrast, commercially available monoclonal antibodies, such as daratumumab and isatuximab, are potent cytotoxic antibodies, in use for the treatment of Multiple Myeloma. TNB-738 also does not mediate antibody-mediated internalization (5). Altogether, these data demonstrate that TNB-738 strongly inhibits the hydrolase activity of human CD38, resulting in increased intracellular NAD+ and sirtuin1 levels.

### TNB-738 prevents GvHD and supports long-term engraftment of human T cells in NSG mice

**Figure 2:**
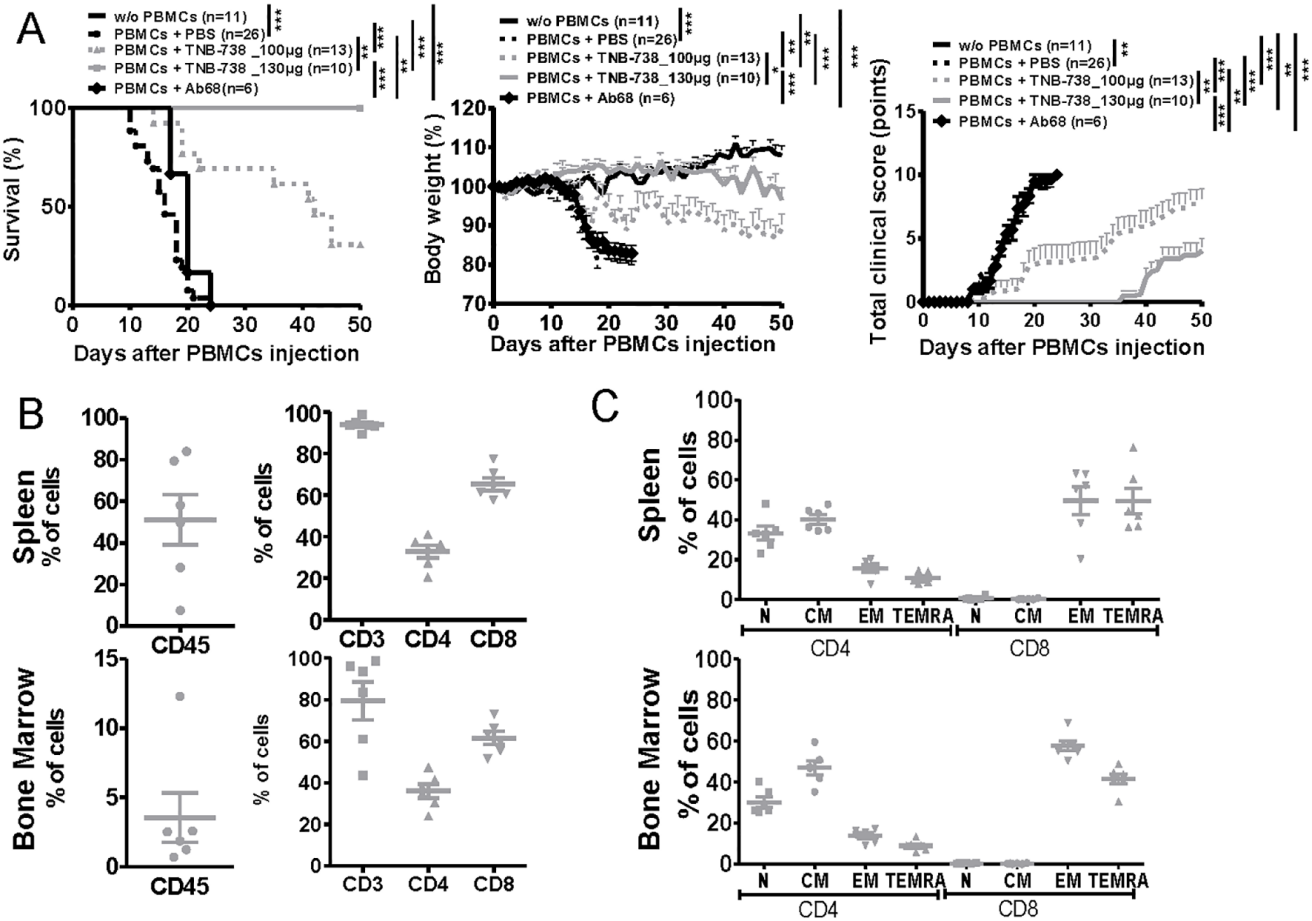
TNB-738 prevents Xeno-GvHD in humanized NSG mice with long-term survival of human T cells at day 50. **(A)** NSG mice were irradiated (1.5 Gy) and 24h later received hPBMC i.v (day 0) or not (black line). Animals were then treated with i.p injections of PBS (dotted black line); TNB-738 at 100µg (dotted gray line) or 130µg (gray line), anti-mouse CD38 Ab68 as a control at 100µg (black line with lozenge) from day 0 to day 18 twice a week. Mice were sacrificed when they lost 20% of initial body weight (IBW). Left graph represents survival curve, Middle graph shows mean of body weight in each group as a percentage of IBW, right graph shows total clinical score as a mean for each group. (data from 3 independent experiments). **(B)** Percentage of CD45+ cells (left graph) or CD3+, CD4+ and CD8+ cells (right graph) in spleen (upper graph) or bone marrow (lower graph) in mice treated with TNB-738 130µg at day 50. **(C)** Percentage of Naïve hCCR7+CD45RA+ (N), Central Memory hCCR7+CD45RA- cells (CM), Terminal effector memory CD45RA+ cells hCCR7-CD45RA+ (TEMRA) and Effector Memory hCCR7-CD45RA- (EM) on hCD4+ and hCD8+ from spleen (upper graph) or bone marrow (lower graph), *p<0.05 **p<0.01 ***p< 0.001.

To assess the potential of inhibiting CD38 hydrolase activity in preventing GvHD, NSG mice injected with human PBMCs were treated with TNB-738 and clinical status followed over time. We observed that TNB-738 treatment significantly delayed GvHD occurrence in a dose-dependent manner and mean survival was extended from day 15.25 (± 3.5) of untreated control mice to day 37.1 (± 13.7) and more than 50 days in mice receiving TNB-738 at 100 µg or 130 µg per injection, respectively (**Figure 2A**). In addition, clinical scores of both TNB-738 groups were significantly lower than the control group (mean of 9.3 for PBS group, 7.8 for TNB-738 100 µg group and 4 for TNB 738 130 µg group). Even if no death was observed at the highest dose of TNB-738 group some mice showed a low clinical score (2/10 mice had no clinical signs of GvHD, 5/10 mice had clinical score between 1 and 5 and, 3/10 mice had a clinical score between 6 and 9). These clinical scores were attributed primarily to severe ruffling and poor grooming of fur texture, accompanied by moderate hunching, slight decrease in activity and some weight loss (**Figure 2A**, right graph). To evaluate the role of mouse CD38 in our xeno-GvHD model, NSG mice were injected with human PBMCs and treated with an anti-mouse CD38 (Ab68) that specifically inhibits mouse CD38 hydrolase activity. Ab68 is an anti-CD38 antibody with a silenced mouse IgG2a Fc tail, which nullifies all Fc mediated functions (1). All mice treated with Ab68 were sacrificed before D25 due to severe loss of weight and clinical signs of disease (**Figure 2A**). In conclusion, only inhibition of human CD38, but not mouse CD38 hydrolase activity, prevented xeno-GvHD in our model. At day 50 all mice from the TNB-738 (130 µg) group were sacrificed and spleens and bone marrows (BM) analyzed. Both tissues contained human CD45 cells; on average 51% of all cells were human cells in spleens and 3.5% in BM. Subpopulations of human immune cells in spleens and BM were similar; majority of cells were positive for human CD3 (mean: 93.9% in spleen and 79.4% in BM). Of these human CD3+ cells in the spleen, 32.8% were CD4 and 65.2% CD8 cells. In the bone marrow, 36% of human CD3+ T were CD4 and 61.6% CD8 cells (**Figure 2B**, **Supplementary Figure 1A**). No overt GvHD was observed in the high TNB-738 group, but human T cells did express the activation marker CD69 (12% of spleen and 26% of bone marrow CD4+cells and 25% of spleen and 48% of bone marrow CD8+ cells - **Supplementary Figure 1B**). These results indicate that human T cells survive long term in TNB-738 treated humanized mice and that a significant percentage of these cells express an activation marker. We also analyzed whether these surviving human T cells at day 50 were naïve or memory T cells. Similar distributions were observed in spleen and BM, namely (1); roughly 70% of CD4+ T cells were naïve and central memory cells (33% naïve, 40% Central Memory, 10% Terminal Effector Memory T cells (TEMRA) and 16% Terminal effector cells in the spleen) (2), majority of CD8+ T cells in both tissues were terminal effector memory cells and TEMRA cells (0.7% naïve, 0.3% Central Memory, 49% TEMRA and 50% Terminal effector cells in the spleen) (**Figure 2C**, **Supplementary Figure 1C**).

### Graft versus tumor response in humanized NSG mice treated with TNB-738 was preserved

**Figure 3:**
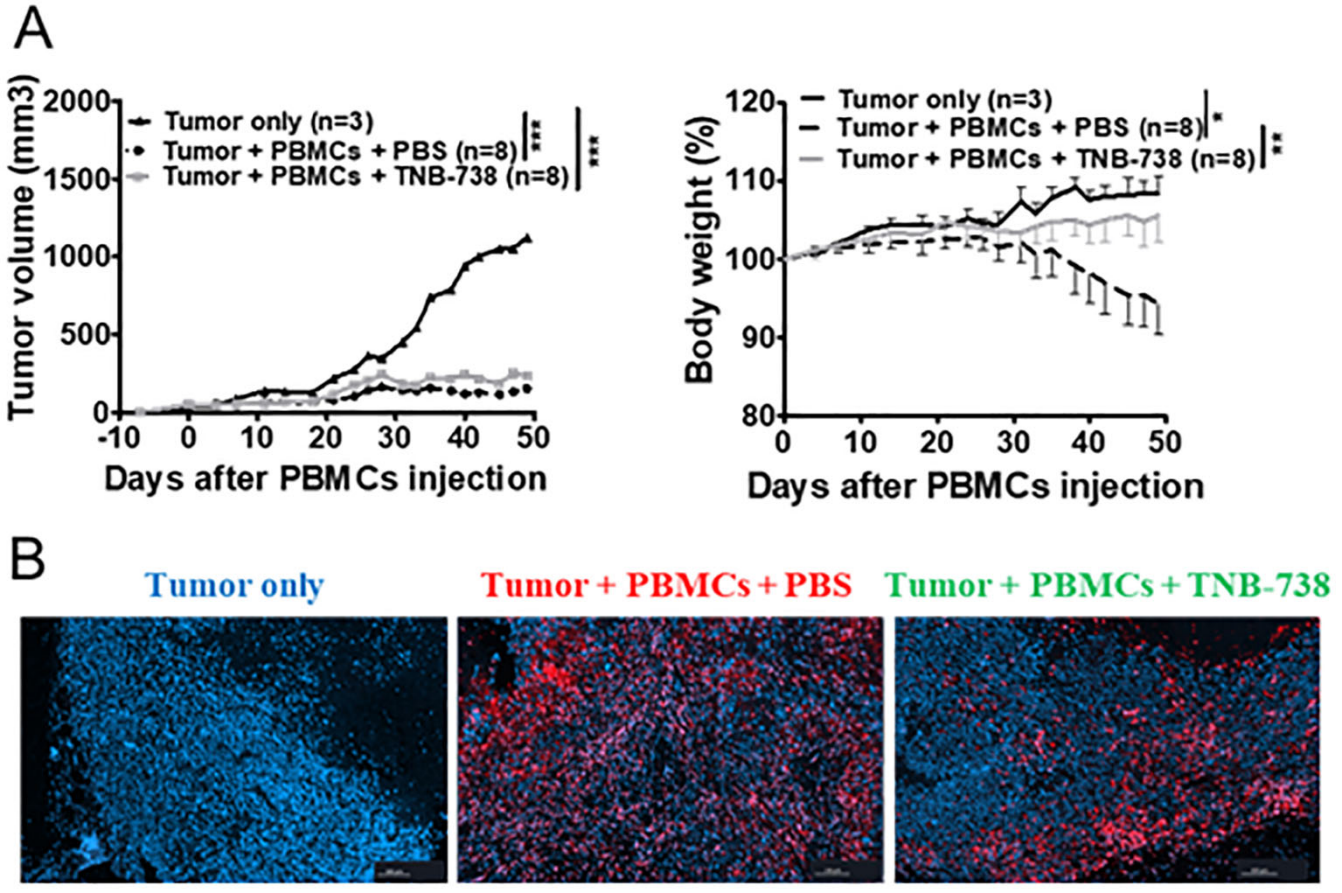
Treatment with TNB-738 inhibits acute GVHD mediated by T cells, preserves graft versus tumor responses. **(A)** NSG mice received 5x10^6^ MDA-MB-231 breast cancer cells subcutaneously (s.c). When the tumor was detected, 1x10^7^ hPBMCs were injected intravenous (i.v) (day 0) or not (Black line). Animals were then treated with intraperitoneal (i.p) injections of PBS (Dotted black lines) or TNB-738 at 130µg (gray line) from day 0 to day 18 twice a week. Mice were sacrificed when they lost 20% of initial body weight (IBW) or if tumor volume > 3000 mm^3^. The left graph shows tumor volume as a mean for each group, right graph represents mean of body weight in each group as a percentage of IBW, *p<0.05 **p<0.01 ***p< 0.001. **(B)** At D50 tumors were harvested and analyzed by immunochemistry. Pictures show one representative image of tumor-only group (left picture), PBS treated group (middle picture) or TNB-738 treated group (right picture). Red staining corresponding to CD3+ cells and nucleus stained with Dapi (blue).

To evaluate the capacity of human T cells from TNB-738 treated mice to reject tumors, we established a Graft versus Tumor (GvT) model using a human breast cancer cell line (MDA-MB-213 cells) in humanized NSG mice (**Figure 3A**). Tumor cells were injected subcutaneously and on day eight 10^7^ hPBMC were injected with TNB-738 or PBS. In the absence of hPBMC, tumor growth increased with time and mice were sacrificed when tumor volume reached 3000 mm^3^. Mice infused with hPBMC rejected MDA-MB-213 tumors (**Figure 3A**). Tumors were rejected both in PBS control and TNB-738 treated animals (**Figure 3A**, left graph), suggesting that TNB-738 did not impair the GvT effect of infused cells. At the same time TNB-738 treated mice neither lost weight nor developed overt GVHD (**Figure 3A**, right graph). Histological analysis showed that human T cells infiltrated tumors (**Figure 3B**), suggesting that GvT was T cell mediated. Thus, TNB-738 prevented acute GvHD without impacting the anti-tumor response in a GvT model in humanized NSG mice.

### TNB-738 treatment leads to increased levels of NAD+ and sirtuin1 while decreasing the expansion of hCD45 and hCD3 positive cells

**Figure 4:**
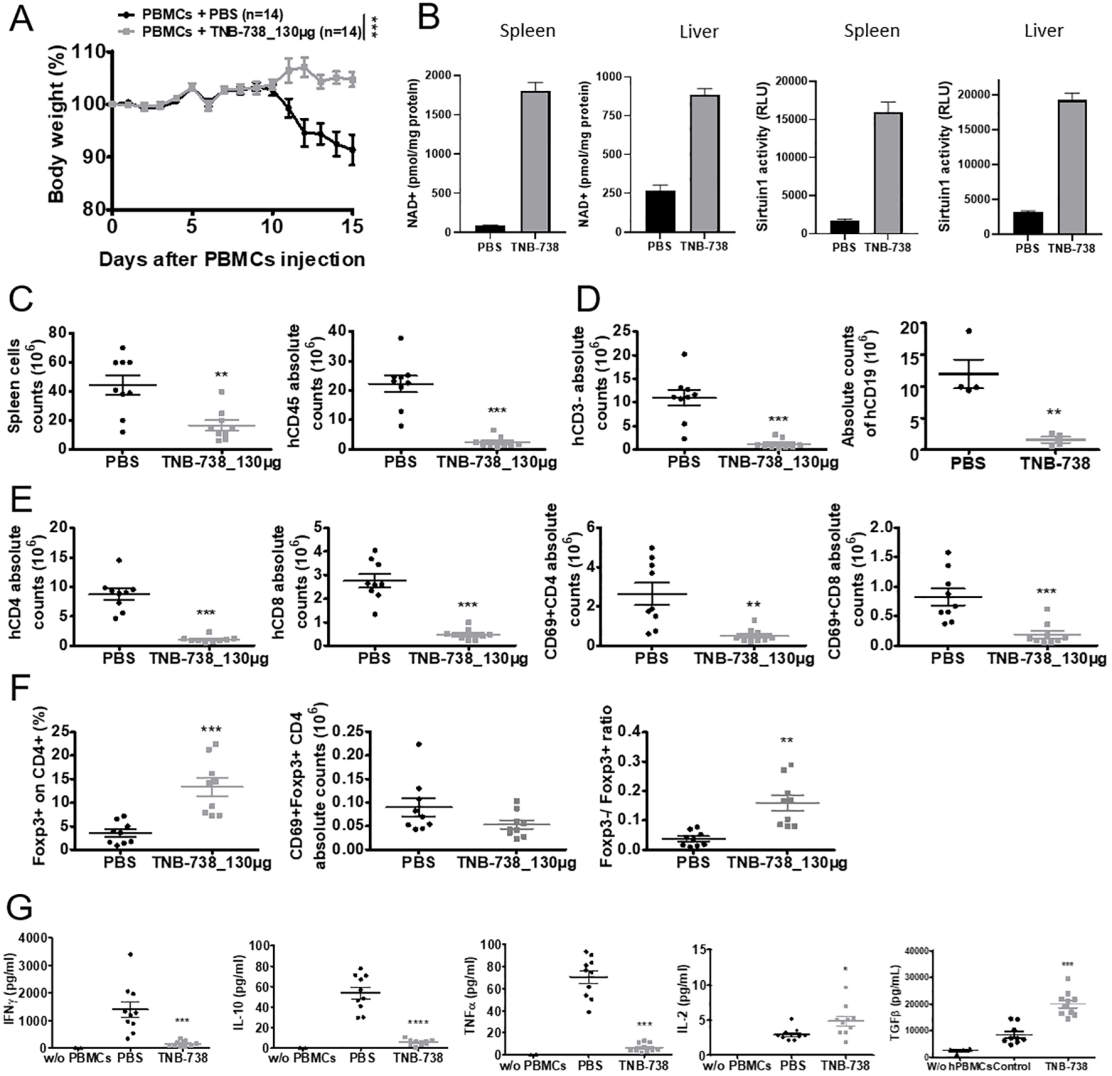
TNB-738 reduces inflammation in tissues at day 15 by limiting the expansion of human CD45 cells with relatively high Treg cell numbers. **(A)** NSG mice were irradiated and 24h later received hPBMCs intravenous (i.v) (day 0). Animals were then treated with intraperitoneal (i.p) injections of PBS (black line) or TNB-738 at 130µg (gray line) twice a week. All mice were sacrificed when mice from PBS group lost 20% of IBW, (datas from 7 independents experiments). Graph represents mean of body weight in each group as a percentage of IBW. **(B)** Protein was extracted from frozen spleen and liver tissues followed by protein estimation and analysis of NAD+ concentration (left 2 graphs) or sirtuin1 activity (right 2 graphs). **(C)** Left graph shows total number of spleen cells in PBS (black point) or TNB-738 (gray scare), right graph shows absolute number of hCD45+ spleen cells in PBS (black point) or TNB-738 (gray scare). **(D)** Graph shows total number of hCD3- (left graph) or hCD19+ (right graph) spleen cells in PBS (black point) or TNB-738 (gray scare). **(E)** Left graph shows total number of hCD4+ spleen cells in PBS (black point) or TNB-738 (gray scare), middle left graph shows absolute number of hCD8+ spleen cells in PBS (black point) or TNB-738 (gray scare), middle right graph shows total number of hCD4+CD69+ spleen cells in PBS (black point) or TNB-738 (gray scare), right graph shows absolute number of hCD8+CD69+ spleen cells in PBS (black point) or TNB-738 (gray scare). **(F)** Left graph shows percentage of hCD4+FOXP3+spleen cells in PBS (black point) or TNB-738 (gray scare), middle graph shows absolute number of hCD4+FOXP3+hCD69+ spleen cells in PBS (black point) or TNB-738 (gray scare), right graph shows hCD4+FOXP3-/hCD4+FOXP3+ ratio on spleen cells in PBS (black point) or TNB-738 (gray scare). **(G)** Cytokine levels in sera of from left to right: hIFNg, hIL-10, hTNF-a, hIL-2 or hTGFb levels in sera of PBS (black point), TNB-738 (gray scare) or NSG that did not receive hPBMC (black triangle). *p<0.05 **p<0.01 ***p< 0.001 ****p<0.0001.

To further determine the effect of TNB-738 on hPBMC mice were sacrificed at Day 15 after injection of hPBMC and TNB-738. At D15 none of the TNB-738-treated mice lost weight, whereas PBS treated mice lost significant weight (**Figure 4A**). Analysis of NAD+ and sirtuin1 activity in spleens and livers indicated that TNB-738 treatment significantly increased NAD levels (15 fold higher in spleens and 3.5 fold higher in livers) and sirtuin1 activities compared to PBS mice (7.5 fold higher in spleens and 4.5 fold higher in livers) (**Figure 4B**). In conclusion, TNB-738 inhibited CD38 enzyme activities *in vivo*, which resulted in higher concentrations of NAD+ and higher activities of sirtuin1. At D15, we observed a significant decrease of hCD45+ in spleens of TNB-738 treated animals (mean of 2.1x10^7^ cells in PBS group *vs* 4x10^6^ cells in TNB-738 group) (**Figure 4C**). In our NSG model of GvHD, hCD45+ infiltrating cells were mainly composed of T and B cells. Analysis of T cell subpopulations showed lower absolute numbers of CD4+ and CD8+ T cells in the TNB-738 group compared to PBS (8 times fewer CD4+ T cells and 6 times fewer CD8+ T cells in TNB-738 treated mice) (**Figure 4E**). Although absolute numbers of CD4+FOXP3+ T cells were equivalent in TNB-738 and PBS treated groups, percentage of CD4+FOXP3+ T cells in spleens of TNB-738-treated mice was significantly higher (13.4% in TNB-738 group and 3.62% in control PBS group) (**Figure 4F**). We analyzed activation of T cells by CD69 expression, which was significantly lower in CD4+ and CD8+ population in TNB-738 treated mice (5.2 times fewer CD4 and 4.4 times fewer CD8 T cells expressed CD69 in TNB-738 group) (**Figure 4E**). In contrast, CD69 expression of FoxP3+CD4+ cells showed no significant difference between groups (**Figure 4F**, middle graph). TNB-738 treated mice had significantly fewer hCD3-/hCD45+ cells in spleens, which were hCD19+ B cells (**Figure 4D**; mean of 1.1x10^7^ CD19+cells in PBS group versus 1x10^6^ CD19+ cells in TNB-738 group). Study of cytokine levels in sera of mice at D15 showed significantly lower IFN-γ (9.2 times less in TNB-738 group), IL-10 (9.7 times less in TNB-738 group) and TNF-α (11.03 times less in TNB-738 group) but a significant increase of IL-2 (1.7 times more in TNB-738 group) and TGF-β (2.4 times more in TNB-738 group) (**Figure 4G**). These data together indicate that TNB-738 treatment resulted in reduced expansion of hCD45 positive cells, while preserving the Treg CD4+FoxP3+ population either by conservation of pre-existing Tregs or emergence of new peripheral Tregs (pTregs). The reduced expansion of hCD45+ cells was associated with lower levels of IFNγ, IL-10 and TNF-α in sera of TNB-738 treated mice, but higher levels of IL-2 and TGF-β.

### Effect of TNB-738 treatment on GvHD is partially mediated by preservation of Treg cells

**Figure 5:**
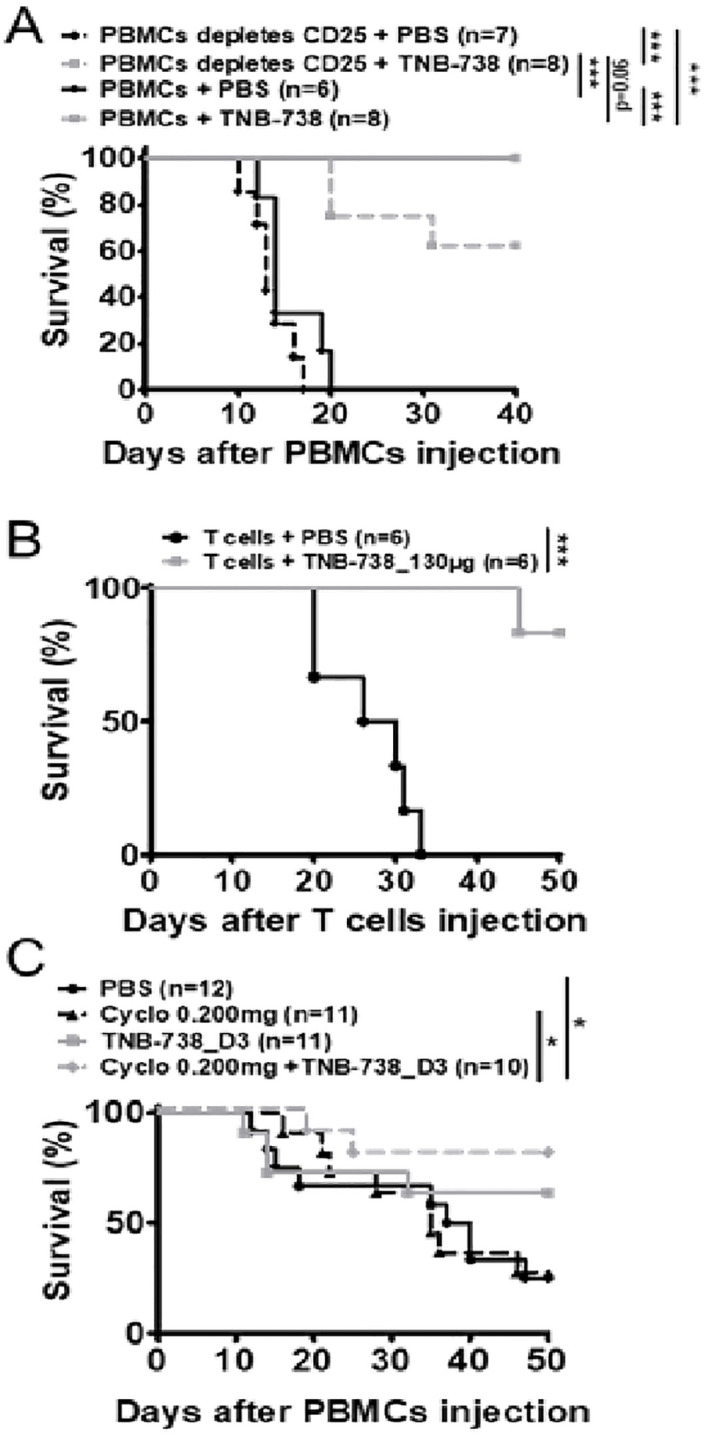
Treatment with TNB-738 inhibits acute GVHD mediated by T cells while preserving graft versus tumor responses. **(A)** NSG mice were irradiated and 24h later received hPBMC (solid lines) intravenous (i.v) (day 0) or hPBMC depleted of CD25+ cells (dotted lines). Animals were then treated with intraperitoneal (i.p) injections of PBS (black lines); TNB-738 at 130µg (gray line) day 0 to day 18 twice a week. Mice were sacrificed when they lost 20% of initial body weight (IBW). Graph represents survival curve (Data from 3 independent experiments). **(B)** NSG mice were irradiated and 24h later received purified human T cells intravenous (i.v) (day 0). Animals were then treated with intraperitoneal (i.p) injections of PBS (black line); or TNB-738 at 130µg (gray line) day 0 to day 18 twice a week. Mice were sacrificed when they lost 10-20% of initial body weight (IBW). Graph represents survival curve (Data from 2 independent experiments). **(C)** NSG mice were irradiated and 24h later received hPBMCs i.v (day 0). Animals were treated from D3 to D21 with intraperitoneal (i.p) injections of PBS (black line); TNB-738 130 µg (gray line), Cyclosporine 0.200 mg (dotted black line) or a combination of cyclosporine and TNB-738 (dotted black line). Graph represents survival curve *p<0.05 **p<0.01 ***p< 0.001.

As we observed a relative increase of Treg cells in TNB-738 treated animals, we evaluated whether Treg cells were essential for the effectiveness of TNB-738. To answer this hypothesis, immunodeficient NSG mice were injected with human PBMCs depleted of CD25 bright cells (**Figure 5A**, **Supplementary Figure 2A**). We observed significant differences between survival of mice treated with TNB-738 (gray curves) and injected with hPBMC depleted of CD25+ cells compared to PBS treated mice (black curve). At D40, GvHD occurred in 38% of mice that received hPBMC depleted of hCD25+ cells plus TNB-738 compared to no GvHD in mice that received total hPBMC cells plus TNB-738 (data not shown). However, survival in these two groups did not reach statistical significance (p=0.06).

To further investigate the role of T and B cells in our model of GvHD, immunodeficient NSG mice were injected with purified CD3+ cells (positively selected with microbeads) and treated with TNB-738 or with PBS. 1 out of 6 mice developed GvHD at D45 in the TNB-738 group, whereas all PBS control mice developed GvHD before day 32 after injection of human T cells (**Figure 5B**). In parallel, we observed a significant difference in body weight of TNB-738 treated mice compared to control mice (**Supplementary Figure 2B**). Because GvHD induces tissue damage, we harvested liver and colon for immunohistology analyses. Tissue damage was scored by an independent pathologist blinded for treatment groups. Results showed a significantly lower score in the TNB-738 group compared to the PBS control group (Mean score of 3.5 in PBS control *vs* 0.4 in TNB-738 for liver and mean score of 2.6 in PBS control *vs* 0.4 in TNB-738 for colon) (**Supplementary Figure 3A, B**). Focal infiltration of the lamina propria by human T cells and necrotic cells were observed in crypts of colons of PBS treated mice, which were absent in TNB-738 treated mice. Livers of PBS control mice showed perivascular cuffing in portal areas and infiltration of surrounding liver parenchyma; some isolated necrotic hepatocytes were also observed. In contrast, TNB-738 treated mice showed no signs of inflammation, which were similar to livers of wild-type NSG mice (**Supplementary Figure 3B**).

Cyclosporin is an immunosuppressive therapeutic commonly used in bone marrow transplant patients. We wondered whether combining a sub-optimal dose of cyclosporin, which inhibits T-cells proliferation by blocking IL-2 transcription [19], and suboptimal dosing of TNB-738 are effective in suppressing GvHD. In contrast to our previous experiments in which we started treatment with TNB-738 at day 0 after injection of hPBMC in NSG mice, we administered TNB-738 at day 3 after hPBMC injection. We observed no statistical differences in survival between the cyclosporin and TNB-738 treated animals, only the combination of both treatments showed a statistical increase of survival compared to PBS control mice (**Figure 5C**, **Supplementary Figure 2C**). In conclusion, combining sub-optimal cyclosporin and TNB-738 treatments worked synergistically in our model of GvHD in mice.

### Proliferation of T cells was reduced upon treatment with TNB-738

**Figure 6:**
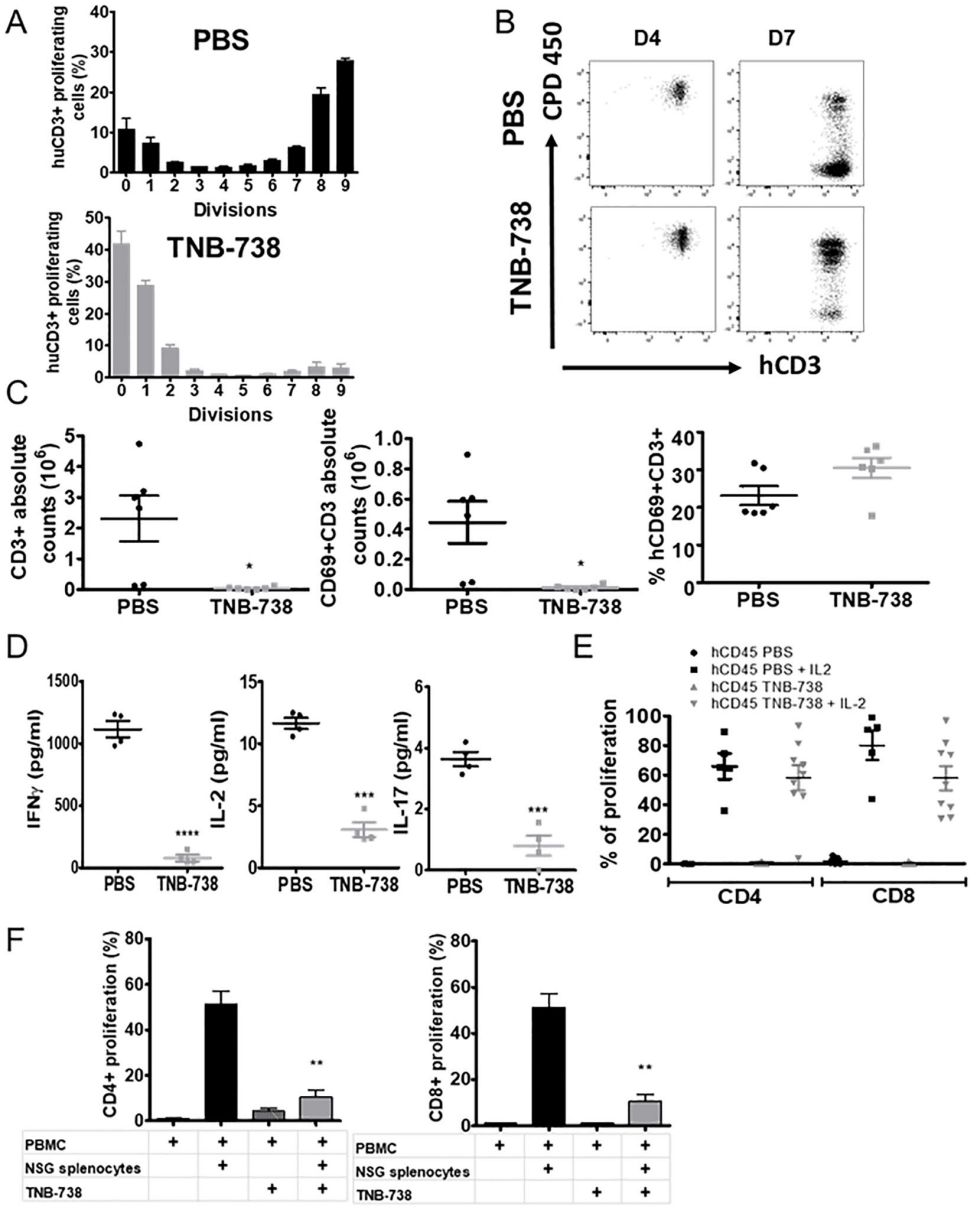
Treatment of GVHD induced NSG mice with TNB-738 causes reduction in T cell proliferation and inflammatory cytokines in serum. **(A)** NSG mice were irradiated and 24h later received CPD-labeled hPBMCs intravenous (i.v) (day 0). Animals were then treated with intraperitoneal (i.p) injections of PBS (Upper graph, black histograms) or TNB-738 at 130µg (Lower graph, gray histogram) twice a week. Mice were sacrificed at D7. Histograms represent percentages of each CPD dividing hCD3+ populations. **(B)** Significative dot plots of hCD3/CPD staining in PBS group (upper graphs) and TNB-738 group (lower graphs) at D4 (left graphs) or D7 (right graph). **(C)** Spleen cells of D7 treated mice were analyzed by cytometry. Graphs show PBS (black circle) or TNB-738 (grey square) from left to right: absolute number of hCD3+; absolute number of CD3+CD69+ cells and percentage of CD3+CD69+ cells. **(D)** Cytokine level in sera: IFNγ (left graph), IL-2 (middle graph) or IL-17 (right graph), in PBS (black round) or TNB-738 (grey square) groups at D7. **(E)** hCD45 from spleen cells of D15 treated mice were sorted and cultured with or without IL-2. Graph represents percentage of proliferation of hCD4+ or hCD8+ in each condition. **(F)** *In vitro* inhibition of proliferation by TNB-738 in CD4+ or CD8+ populations. hPBMCs were cultured with or without NSG splenocytes in presence or not of TNB-738 for 7 days. At D7 proliferation of hCD4+ (left histograms) (CPD staining) or hCD8+ (right histograms) were analyzed in each group. Graphs represent means of percentage of proliferation (mean of 7 experiments); * p<0.05 ** p<0.01, ***p< 0.001, ****p<0.0001.

To study the kinetics of proliferation of T cells, we labeled donor cells with CPD450 and harvested cells at different timepoints. Proliferation of T cells was analyzed by harvesting spleens at Day 4 (D4) and Day 7 (D7) after injection of hPBMC. At D4, we observed no proliferation of hCD45+ cells in both groups (**Figure 6B**). At D7, we observed significantly more proliferation of hCD45+ cells in PBS treated mice compared to TNB-738 treated mice (**Figures 6A, B**). At D7, 50% of hCD45 cells in PBS control mice were in the eighth or ninth generation, and fewer than 10% of hCD45+ cells did not proliferate. In contrast, less than 10% of hCD45+ cells of TNB-738 animals were in the 8^th^ or 9^th^ generation with roughly 40% of the cells not proliferating (**Figure 6A**). Proliferating cells were mainly CD3+ cells (**Figure 6B**). Inhibition of proliferation by TNB-738 was also observed *in vitro* using hPBMC co-cultured with NSG splenocytes (**Figure 6F**). In the same experiment we harvested sera and showed statistically lower levels of cytokines (IFNγ, IL-10, TNF-α, IL-2, IL-6 and IL-17) in animals treated with TNB-738 (**Figure 6D**). CD69 is an activation marker for T cells and expression of human CD69 was analyzed on T cells. Although we observed statistically lower absolute number of CD3+ and CD3+CD69+ cells in TNB-738 treated animals we did not observe a statistical difference in the percentage of CD3+CD69+ cells (**Figure 6C**). These results indicate lower proliferation and lower absolute numbers of T cells in TNB-738 treated animals. To assess whether T cells became anergic in the presence of TNB-738, we extracted hCD45 cells from spleens on day 15 of treatment and cultured these cells with or without IL-2. hCD4+ and hCD8+ cells recovered from spleens from all groups proliferated *ex vivo* identically in the presence of exogenous IL-2 (**Figure 6E**) indicating that T cells of TNB-738 treated mice were not anergic. Taken together with the fact that TNB-738 treatment preserved the GvT response, these results indicate that inhibition of the CD38 hydrolase activity on T cells reduces local inflammation of organs while maintaining tumor infiltration and lysis by T cells.

### RNA sequencing of purified hPBMC and untargeted metabolomics of liver tissue from xeno-GvHD mice treated with TNB-738 revealed distinct patterns of reduced inflammatory and apoptosis markers

**Figure 7:**
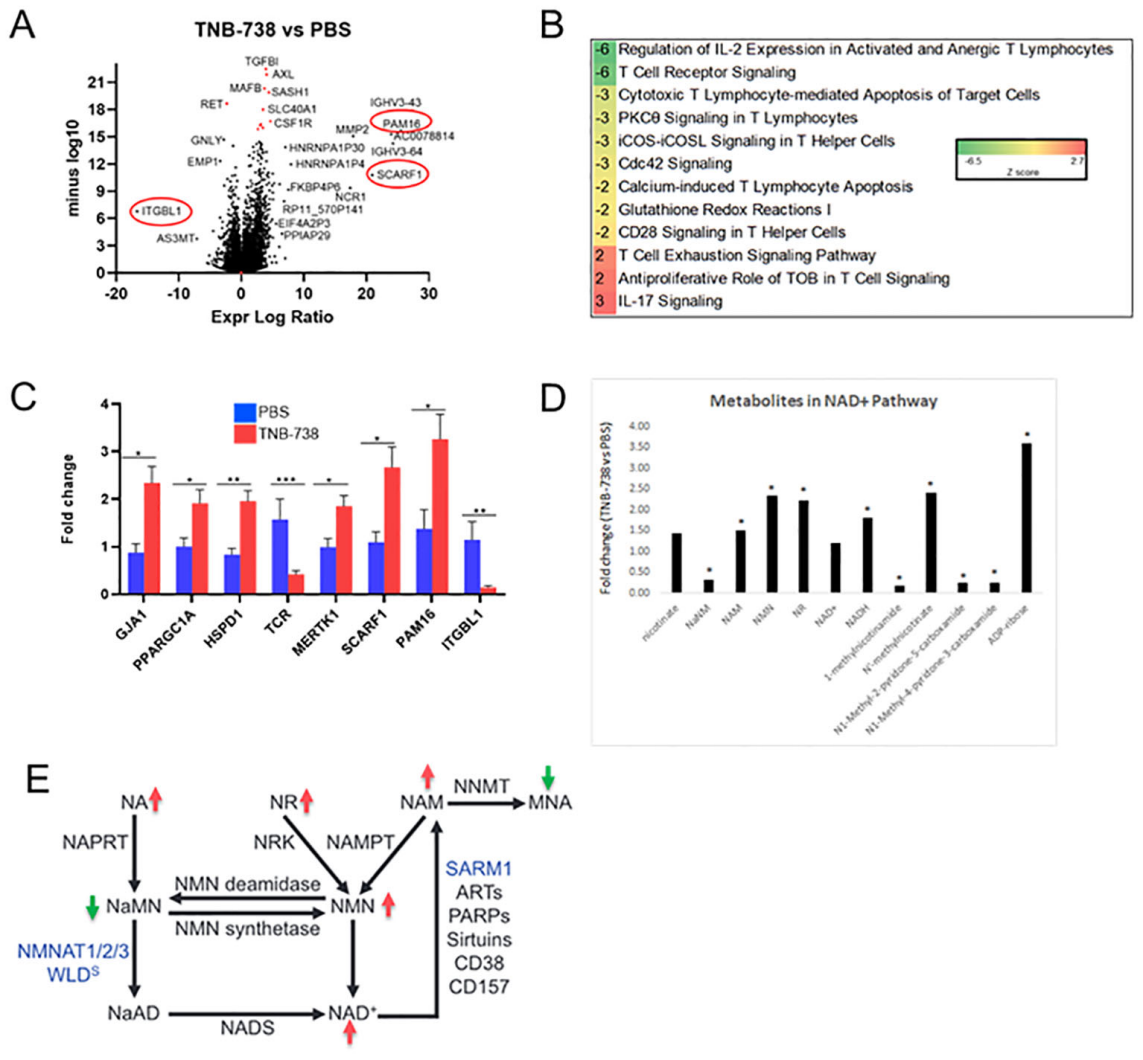
TNB-738 modulates T cell signaling by down-regulating pro-proliferation pathways at day 15 as determined by RNAseq and untargeted metabolomics. Harvested mouse livers on day 11 of treatment with TNB-738 or PBS were used for RNAseq and untargeted metabolomics. **(A)** Volcano plot highlighting significantly affected genes after TNB-738 treatment compared to PBS. **(B)** Ingenuity Pathway Analysis depicting the most affected signaling cascades due to TNB-738 treatment. **(C)** Some of the significantly differentiated genes based on the RNAseq data were verified using qPCR **(D)** Untargeted metabolomics revealed key metabolites differentially present in the liver of TNB-738 treated mice. **(E)** Summary of metabolic changes induced in mouse liver by TNB-738 treatment.

To precisely identify the signature molecules responsible for the immunomodulatory effect of TNB-738 on xeno-GvHD mice, we performed RNA sequencing of hPBMC after sacrificing the animals at day 11 of treatment and magnetic bead sorting for human CD45 cells. The volcano plot presented in **Figure 7A** includes all 17,000 genes that were expressed as calculated by DESeq2.

We observed multiple differentially expressed genes (DEG) between TNB-738 and PBS-treated animals (FDR Q-value <0.05), 241 genes with a log 2-fold change >1 (**Supplementary Table 1**). We validated the expression of PAM16, SCARF1 and ITGBL1, genes observed on either extreme sides of the X axis on the volcano plot by qRT-PCR and observed similar results (**Figure 7C**). Additionally, we verified by qRT-PCR the increased expression of GJA1, PGC1α and HSPD1; mitochondrial homeostasis markers involved in NAD+ pathway regulation (**Figure 7C**). Importantly, downregulation of TCR and MERTK genes by TNB-738 correlated with our *in-vivo* observations of reduced inflammation and T cell activation in treated mice (**Figure 7B**). The differential pathway summary of RNAseq analysis shown in **Figure 7C** implies that the genes that connect to GvHD signaling are modulated by TNB-738 treatment and that blocking CD38 enzyme activity controls hyper-activation of immune cells without clearing them.

Metabolomic analysis showed that TNB-738 treatment resulted in significantly increased levels of ADP ribose, nicotinate and nicotinamide metabolites and trending higher levels of NAD+ (**Figure 7D**). The markers of fatty acid synthesis e.g., malonate and malonylcarnitine were significantly increased in the liver post TNB-738 treatment along with significantly higher acyl-CoAs and acylcarnitines. TNB-738 maintains mitochondrial homeostasis by lowering 3-hydroxybutyrate (BHBA) levels and caused significantly decreased levels of numerous dihydroceramides, ceramides, and hexosylceramides in the liver, an indication of decreased inflammation and tissue turnover in the liver. Elevated levels of glutathione recycling metabolites, unchanged GSSG, increased GSH indicated reduced oxidative burden with TNB-738 treatment. Several readouts of inflammation were significantly decreased after TNB-738 treatment including 9-HODE and 13-HODE, dihydroxy fatty acids and eicosanoids. Together, decreased levels of these metabolites suggest that antibody treatment is reducing reactive oxygen species generation and inflammation (**Table 4**).

**Table 4:** Intracellular metabolites of livers of TNB-738 treated versus control animals indicate reduced oxidative stress, inflammation, and tissue turnover and improved energy balance.

Metabolite(s)	TNB-738 *vs* control	Biological relevance
NAD+, NMN, NR, NA		Increased energetics, redox reactions and/or posttranslational modifications
ADP Ribose		Altered poly ADP ribose polymerase activity and/or ADP ribosylation
Glycogen Metabolism		Differential glycogen synthesis or degradation
Acylcarnitines		Elevated fatty acid beta oxidation
BHBA		Reduced ketone body accumulation
Dicarboxylic fatty acids		Increased omega-oxidation, altered CYP hydroxylase activity
Aminosugars		Changes in glycosylation patterns
MAGs, Glycerophosphoglycerol		Altered lipolysis or lipid synthesis
Bile acids		Increased lipid absorption
Ceramides		Modulation of cell membrane composition and/or integrity, or lipoprotein particle composition and abundance. Decreased inflammation
Plasmalogens		Differential inflammation or ER stress
Inflammatory mediators		Differential inflammation and/or fibrosis
Dipeptides, Lysine Catabolites		Decreased protein degradation and/or tissue turnover
Purines and Pyrimidines		Decreased protein degradation and/or tissue turnover

Key: Significantly decreased; Significantly increased.

## Discussion

We present in this study, a collective scientific rationale to support TNB-738, an anti-CD38 enzyme inhibitor antibody, as prophylaxis and/or treatment of inflammatory diseases, such as acute GvHD in patients undergoing allogeneic bone marrow transplantations. Our *in vitro* and *in vivo* data show that inhibition of the CD38 hydrolase activity on T cells increases intracellular NAD+ levels of CD38-positive immune cells, but also of surrounding tissues devoid of human CD38. Treatment with TNB-738 in an animal model of severe inflammation mediated by infused human T cells lead to long-term survival of animals and greatly reduced inflammation in tissues. Human T cells recovered from spleens and bone marrows of treated animals were functional, survived long-term and rejected implanted tumor cells. Analysis of the transcriptome and metabolome of T cells and tissues revealed significant down-regulation of T cell signaling pathways and other inflammatory pathways, reduced oxidative stress and improved mitochondrial health. All our data together indicate that CD38 on T cells can play a dominant role in NAD+ metabolism and that inhibition of its enzyme function can modulate immune responses.

Inhibition of CD38 on primary immune cells, such as activated T cells and macrophages *in vitro* resulted in increased intracellular NAD+ concentrations and activation of NAD-dependent enzymes, such as Sirtuin 1. *In vivo*, inhibition of human CD38 by our human-specific TNB-738 antibody, but not our mouse-specific anti-CD38 antibody, also resulted in increased NAD+ levels and sirtuin1 activities of engrafted human immune cells and murine tissues. Since TNB-738 neither depletes CD38+ cells nor induces internalization of CD38 from the surface, we postulate that CD38 on immune cells can play a dominant role in regulating NAD+ metabolism in inflamed tissues.

Our results show that TNB-738 treatment controls T cells at multiple steps, from reduced T cell expansion and increased Treg numbers to diminished production of cytokines. There is a large unmet need in acute and chronic GvHD treatment. The most common treatment of GvHD is steroids, with ~60% of patients responding to steroids but that results in tumor relapse and risk of infection. Plus ~40% of patients need second line treatment as they are refractory or steroid dependent. Steroid-refractory patients have high mortality. Successful hematopoietic stem cell transplantation requires prophylaxis and a treatment approach that balances avoidance of GvHD while maintaining some donor T cell function against residual tumor cells. TNB-738 in a model of xeno-GvHD reduced tissue inflammation, but T cells survived long-term in the spleen and bone marrow and recovered human T cells from the spleen responded to stimuli ex vivo. Excitingly, the anti-tumor T cell response was conserved with human T cells infiltrating tumors. Long-term protection against xeno-GvHD was achieved with 6 injections of TNB-738 spread over 18 days, suggesting that modulation of T cell responses by blocking CD38 early after allo-HSCT could have significant clinical benefits to patients.

In a study looking at the peripheral blood T cells, it was shown that CD38 bright, CD8+ effector memory T cells (T_EM_ cells) increased 8 days before aGvHD disease manifested itself. Specifically, absolute CD38 bright, CD8+ T_EM_ cell counts in excess of 35 count/μL blood predicted onset of aGvHD (13). This study suggests that CD38 upregulation could be used as an early marker of GvHD onset. The protective role of TNB-738 was effective after injection of purified human T cells in our xeno-GvHD model, suggesting that CD38 on human T cells is the prime target of TNB-738.

TNB-738 treatment led to a higher percentage of Treg cells compared to effector T cells (ratio of Treg positive *vs* Treg negative cells was higher in TNB-738 treated animals). T cells recovered from spleens of TNB-738 treated mice were still functional; human T cells responded ex vivo to IL-2. The protective role of TNB-738 can partially be explained by Treg expansion since depletion of Tregs from the infused T cells resulted in partial loss of protection against aGvHD. This could indicate that in addition to Treg other mechanisms play a role in the suppression of GvHD in this model. It is a matter of further investigation whether the association of TNB-738 with treatments that increase the number or function of Tregs may have an additional benefit. Interestingly, our results showed that most T cells in the bone marrows and spleens of treated mice at day 50 are central memory, terminally differentiated effector memory T (T_EMRA_) cells and effector memory T (T_EM_) cells. This could explain the preserved GvT response, which is mainly mediated by memory T cells and GvHD mainly caused by naïve T cells (14). Histological analysis of tissues confirmed that TNB-738 prevents inflammation in the liver and colon. Inhibition of CD38 on T cells seems to lead to reduced hyper-inflammation and could be relevant to prevent onset of aGvHD in humans.

Infections in steroid refractory-GVHD patients are frequent and commonly severe, significantly contributing to mortality. Additional and better second line options and steroid-sparing treatments are needed. So far, cyclosporine has been one of the most used immunosuppressant for GVHD prophylaxis. Cyclosporin suppresses the transcription of IL-2 and other cytokines, thereby inhibiting the growth and differentiation of T cells. However, cyclosporin at efficacious doses given to GvHD patients, also induces T cell death and kidney toxicity as well as limits GvT responses. Hence, we tested if sub-optimal doses of cyclosporin could control GvHD when combined with a delayed administration of TNB-738, like in a clinical situation in which acute GVHD has been initiated. Administration of TNB-738 with suboptimal cyclosporin showed improved survival over cyclosporin alone, suggesting that this combination could be efficacious. These data suggest that combination treatments with lower doses of cyclosporin and TNB-738 could be employed in allo-HSCT patients.

Among the many roles of Sirtuin 1, it has been shown to modulate immune responses by regulating immune cell activation. In T cells, SIRT1 suppresses T-cell immunity by reducing transcription factors, such as NF-κB and activator protein-1 (15). *SIRT1*-deficient mice show elevated T cell activation and a lupus-like autoimmune phenotype (16). Enhanced p53 acetylation, promotion of induced regulatory T cell (iTreg) differentiation and inhibition of IFNγ production in allo-BMT model with SIRT1^-/-^ T cells has been shown (17). In another study, CD4 T cells from 92 patients with aGvHD were found to have *SIRT1* deficiencies, which caused excessive activation of the mTOR pathway, upregulation of STAT3 acetylation and phosphorylation in CD4+ T cells (18). These studies combined indicate that reduced *SIRT1* leads to more hyper-activated immune cells and severe aGvHD. Pathways associated with increased SIRT1 expression are induced by TNB-738 in our xeno-GvHD model. Transcriptomics analysis by RNAseq of engrafted hPBMC recovered from spleens of mice with xeno-GvHD showed a decrease in the PI3K pathway, which correlated with increased SIRT1 activity and gene expression. SIRT1 is a master metabolic regulator protecting cells from oxidative stress, promoting DNA stability, and decreasing age-related ailments (19). Treatment with TNB-738 was accompanied by increased PGC-1α expression, which could partly explain the anti-inflammatory effects of TNB-738. We also observed significant increase of transcripts related to mitochondrial respiratory health, namely (1): GJA1, a mitochondrial protein that downregulates the intrinsic apoptotic pathway during conditions of oxidative stress, (2) HSPD1, a mitochondrial protein that prevents protein misfolding stress conditions and, (3) PAM16, a mitochondrial protein that regulates reactive oxygen species (ROS) homeostasis. On the other hand, expression of ITGBL1 was greatly reduced, which indicates better cellular respiration and electron transfer in mitochondria. Increased SCARF1 expression indicates improved clearance of apoptotic cells, thus maintaining tolerance. Altogether, the inhibition of CD38 on T cells in an animal model of xeno-GvHD showed regulation of several genes involved in inflammation, T cell signaling, IL-2 signaling, ICOS signaling, and calcium-dependent enzymes involved in T cell proliferation.

Analysis of the metabolome of liver tissues revealed that CD38 inhibition modulated the NAD+ salvage pathway, indicated by high NAD+, NMN and NAM in cells. In contrast, metabolites associated with *de novo* NAD synthetic pathways were largely unchanged or decreased. ADP ribose is a breakdown product of NAD and was significantly increased after TNB738 treatment possibly because of heightened activities of NAD-dependent enzymes, such as NAMPT, PARPs, and Sirtuins. These enzymes are more active due to the abundance of NAD+ in TNB-738 treated animals. TNB-738 treatment increased markers of fatty acid synthesis in the liver indicating that fatty acids were mobilized for fatty acid β-oxidation in the liver. Collectively, these results clearly suggest that CD38 inhibition resulted in elevated levels of fatty acid β-oxidation, which is a beneficial energetic shift that could be important in the treatment of metabolic disorders. A rise in markers of fatty acid oxidation is expected to improve mitochondrial functions. TNB-738 caused significantly decreased levels of numerous dihydroceramides, ceramides, and hexosylceramides in the liver. Declines in ceramides in the liver following TNB-738 treatment may reflect decreased levels of inflammation, oxidative stress, and apoptosis (20). Unchanged levels of GSSG, increased levels of GSH, and elevated levels of glutathione recycling metabolites indicate reduced oxidative burden with TNB738 treatment. In the liver data set, 9- and 13-HODE, dihydroxy fatty acids, eicosanoids, and several other readouts of inflammation were significantly decreased after TNB738 treatment (21). Decreased levels of these metabolites suggest that antibody treatment reduced reactive oxygen species generation and inflammation. TNB-738 treatment in the liver resulted in robust biochemical shifts to carbohydrate and lipid metabolism. Importantly, the biochemical shifts in the liver could suggest decreased *de novo* lipogenesis along with increased lipolysis and fatty acid oxidation, key readouts of metabolic improvement. Significantly decreased levels of 1-methylnicotinamide (MNA), N-methyl-2-pyridone-5-carboxamide, and N-methyl-4-pyridone-3-carboxamide in the liver of TNB738-treated mice may be worth considering as important biochemical shifts related to NAD+ metabolism. MNA is an endogenous product of nicotinamide methylation by nicotinamide N-methyltransferase (NNMT). Importantly, MNA exhibits anti-inflammatory actions (22) and plays a role as an immune regulatory metabolite (23). Moreover, MNA has been reported to inhibit the generation of reactive oxygen species (22) and NNMT has been reported to be upregulated in cancer cells and is generally thought to promote cell proliferation and migration (24). Thus, lower levels of MNA in the liver could reflect reduced NNMT activity which could point to decreased cellular proliferation and tissue turnover. Overall, decreased readouts of oxidative stress, inflammation, and tissue turnover suggest that TNB-738 treatment improved the health of tissues in xeno-GvHD mice.

In conclusion, inhibition of the enzyme functions of CD38 on immune cells *in vitro* and *in vivo* increases intracellular NAD+ levels and activities of NAD-dependent enzymes. This change in NAD metabolism has strong anti-inflammatory effects in an animal model of xeno-GvHD. Human T cells survived long-term in treated NSG mice engrafted with human PBMC. In addition, engrafted T cells were functional indicated by the fact that T cells responded to Il-2 *ex vivo* and rejected implanted tumor cells *in vivo*. Transcriptome and metabolome analysis of human T cells and tissues of TNB-738 treated mice revealed upregulation of anti-T cell proliferative mechanisms and shifts to more favorable energy balances in tissues. In conclusion, TNB-738 has an attractive therapeutic profile for the treatment of inflammatory diseases, including GvHD.

## Data Availability

The original contributions presented in the study are included in the article/**Supplementary Material**, further inquiries can be directed to the corresponding author/s.

## References

[1] Chini CCS Peclat TR Warner GM Kashyap S Espindola-Netto JM de Oliveira GC. CD38 ecto-enzyme in immune cells is induced during aging and regulates NAD+ and NMN levels. Nat Metab. (2020) 2:1284–304. doi: 10.1038/s42255-020-00298-z, PMID: 33199925 PMC8752031

[2] Zeidler JD Hogan KA Agorrody G Peclat TR Kashyap S Kanamori KS. The CD38 glycohydrolase and the NAD sink: implications for pathological conditions. Am J Physiol Cell Physiol. (2022) 322:C521–45. doi: 10.1152/ajpcell.00451.2021, PMID: 35138178 PMC8917930

[3] Shi B Wang W Korman B Kai L Wang Q Wei J. Targeting CD38-dependent NAD+ metabolism to mitigate multiple organ fibrosis. iScience. (2021) 24(1):101902., PMID: 33385109 10.1016/j.isci.2020.101902 PMC7770554

[4] Covarrubias AJ Perrone R Grozio A Verdin E. NAD+ metabolism and its roles in cellular processes during ageing. Nat Rev Mol Cell Biol. (2021) 22:119–41. doi: 10.1038/s41580-020-00313-x, PMID: 33353981 PMC7963035

[5] Ugamraj HS Dang K Ouisse L-H Buelow B Chini EN Castello G. TNB-738, a biparatopic antibody, boosts intracellular NAD+ by inhibiting CD38 ecto-enzyme activity. MAbs. (2022) 14:2095949., PMID: 35867844 10.1080/19420862.2022.2095949 PMC9311320

[6] Tao Z Jin Z Wu J Cai G Yu X. Sirtuin family in autoimmune diseases. Front Immunol. (2023) 14:1186231. doi: 10.3389/fimmu.2023.1186231, PMID: 37483618 PMC10357840

[7] Nikolaenko L Chhabra S Biran N Chowdhury A Hari PN Krishnan A. Graft-versus-host disease in multiple myeloma patients treated with daratumumab after allogeneic transplantation. Clin Lymphoma Myeloma Leuk. (2020) 20:407–14., PMID: 32249196 10.1016/j.clml.2020.01.010 PMC9009296

[8] Cooke KR Hill GR Crawford JM Bungard D Brinson YS Delmonte J. Tumor necrosis factor- alpha production to lipopolysaccharide stimulation by donor cells predicts the severity of experimental acute graft-versus-host disease. J Clin Invest. (1998) 102:1882–91. doi: 10.1172/JCI4285, PMID: 9819375 PMC509139

[9] Feldman J. A mathematical model for tumor volume evaluation using two-dimensions1. J Appl Quantitative Methods. (2009).

[10] Boucault L Lopez Robles M-D Thiolat A Bézie S Schmueck-Henneresse M Braudeau C. Transient antibody targeting of CD45RC inhibits the development of graft-versus-host disease. Blood Adv. (2020) 4:2501–15. doi: 10.1182/bloodadvances.2020001688, PMID: 32511714 PMC7284095

[11] Blazar BR Taylor PA McElmurry R Tian L Panoskaltsis-Mortari A Lam S. Engraftment of severe combined immune deficient mice receiving allogeneic bone marrow via *In utero* or postnatal transfer. Blood. (1998) 92:3949–59. 9808589

[12] Clarke SC Ma B Trinklein ND Schellenberger U Osborn MJ Ouisse L-H. Multispecific antibody development platform based on human heavy chain antibodies. Front Immunol. (2018) 9:3037. doi: 10.3389/fimmu.2018.03037, PMID: 30666250 PMC6330309

[13] Khandelwal P Lane A Chaturvedi V Owsley E Davies SM Marmer D. Peripheral blood CD38 bright CD8+ Effector memory T cells predict acute graft-versus-host disease. Biol Blood Marrow Transplant. (2015) 21:1215–22., PMID: 25881755 10.1016/j.bbmt.2015.04.010

[14] Zheng H Matte-Martone C Li H Anderson BE Venketesan S Sheng Tan H. Effector memory CD4+ T cells mediate graft-versus-leukemia without inducing graft-versus-host disease. Blood. (2008) 111:2476–84., PMID: 18045967 10.1182/blood-2007-08-109678 PMC2234071

[15] Okasha SM Itoh M Tohda S. Sirtuin 1 activation suppresses the growth of T-lymphoblastic leukemia cells by inhibiting NOTCH and NF-κB pathways. Anticancer Res. (2020) 40:3155–61. doi: 10.21873/anticanres.14297, PMID: 32487610

[16] Sequeira J Boily G Bazinet S Saliba S He X Jardine K. sirt1-null mice develop an autoimmune-like condition. Exp Cell Res. (2008) 314:3069–74. doi: 10.1016/j.yexcr.2008.07.011, PMID: 18687325

[17] Daenthanasanmak A Iamsawat S Chakraborty P Nguyen HD Bastian D Liu C. Targeting Sirt-1 controls GVHD by inhibiting T-cell allo-response and promoting Treg stability in mice. Blood. (2019) 133:266–79., PMID: 30514750 10.1182/blood-2018-07-863233 PMC6337874

[18] Xu Y-J Chen F-P Chen Y Fu B Liu E-Y Zou L. A possible reason to induce acute graft-vs.-host disease after hematopoietic stem cell transplantation: lack of sirtuin-1 in CD4+ T cells. Front Immunol. (2018) 9:3078. doi: 10.3389/fimmu.2018.03078, PMID: 30622543 PMC6308326

[19] Pardo PS Boriek AM. SIRT1 regulation in ageing and obesity. Mech Ageing Dev. (2020) 188:111249. doi: 10.1016/j.mad.2020.111249, PMID: 32320732

[20] Sofi MH Heinrichs J Dany M Nguyen H Dai M Bastian D. Ceramide synthesis regulates T cell activity and GVHD development. JCI Insight. (2017) 2(10):e91701. doi: 10.1172/jci.insight.91701, PMID: 28515365 PMC5436544

[21] Ziegler TR Panoskaltsus-Mortari A Gu LH Jonas CR Farrell CL Lacey DL. Regulation of glutathione redox status in lung and liver by conditioning regimens and keratinocyte growth factor in murine allogeneic bone marrow transplantation. Transplantation. (2001) 72:1354–62. doi: 10.1097/00007890-200110270-00004, PMID: 11685103

[22] Biedroń R Ciszek M Tokarczyk M Bobek M Kurnyta M Słominska EM. 1-Methylnicotinamide and nicotinamide: two related anti-inflammatory agents that differentially affect the functions of activated macrophages. Arch Immunol Ther Exp (Warsz). (2008) 56:127–34., PMID: 18373238 10.1007/s00005-008-0009-2 PMC2766500

[23] Kilgour MK MacPherson S Zacharias LG Ellis AE Sheldon RD Liu EY. 1-Methylnicotinamide is an immune regulatory metabolite in human ovarian cancer. Sci Adv. (2021) 7(4):eabe1174. doi: 10.1126/sciadv.abe1174, PMID: 33523930 PMC7817098

[24] Hah Y-S Cho HY Jo SY Park YS Heo EP Yoon T-J. Nicotinamide N-methyltransferase induces the proliferation and invasion of squamous cell carcinoma cells. Oncol Rep. (2019) 42:1805–14., PMID: 31545452 10.3892/or.2019.7315 PMC6787961

