# Supplementary material for: Inhibition of CD38 enzyme activity on engrafted human immune cells enhances NAD+ metabolism and inhibits inflammation in an in-vivo model of xeno-GvHD
Source: Front Immunol. 2025 Oct 13;16:1640611. doi: 10.3389/fimmu.2025.1640611 (PMC12555386; doi:10.3389/fimmu.2025.1640611)
Supplement: Supplementary file 1 [file DataSheet1.pdf]

Supplementary figure 1 :

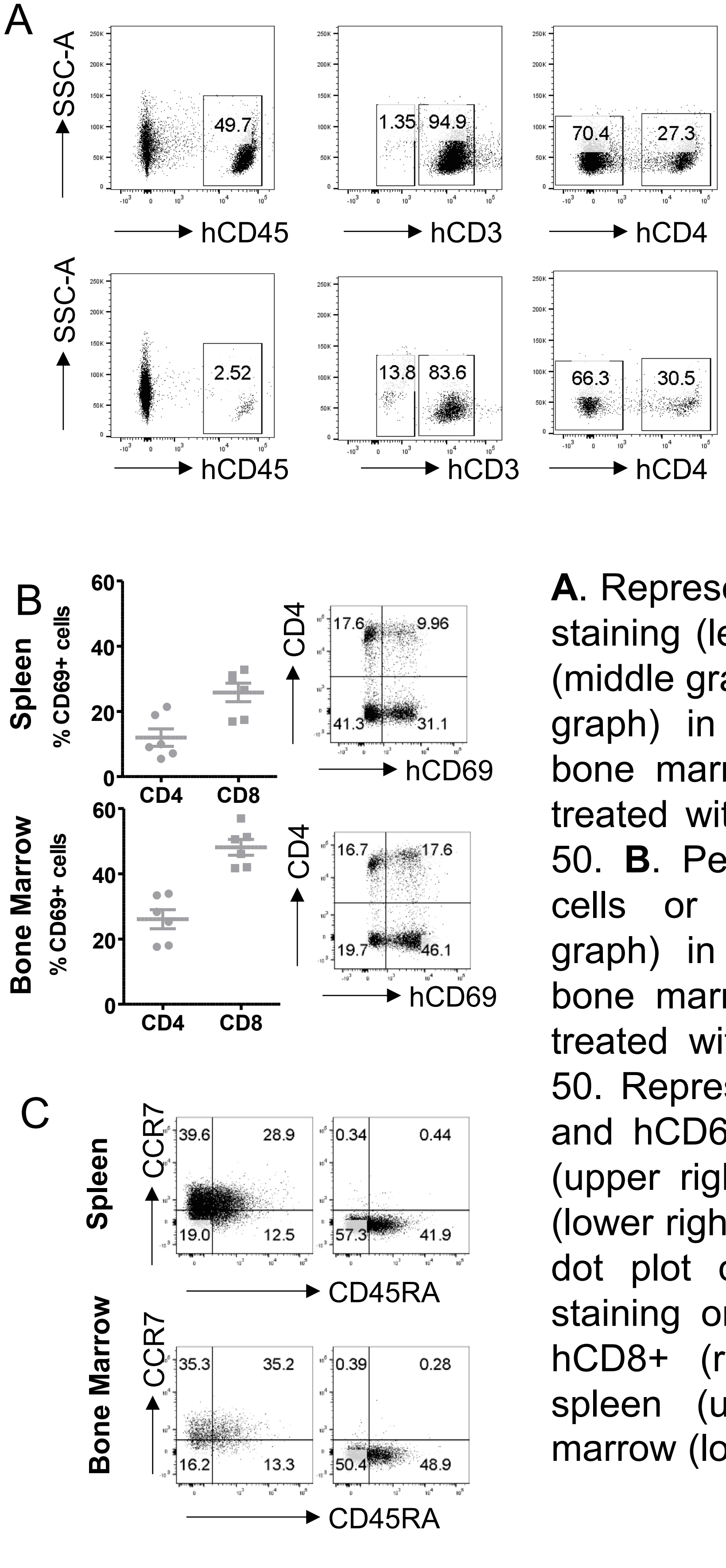

A. Representative dot plot of hCD45+ staining (left graph) or hCD3 staining (middle graph) or hCD4 staining (right graph) in spleen (upper graph) or bone marrow (lower graph) in mice treated with TNB-738 130 µg at day 50. **B**. Percentage of hCD4+CD69+ cells or hCD8+CD69+ cells (left graph) in spleen (upper graph) or bone marrow (lower graph) in mice treated with TNB-738 130µg at day 50. Representative dot plot of hCD4 and hCD69 staining on spleen cells (upper right graph) or bone marrow (lower right graph). **C**. Representative dot plot of hCD45RA and hCCR7 staining on hCD4+ (left dot plot) or hCD8+ (right dot plot) cells from spleen (upper dot plot) or bone marrow (lower dot plot).

Supplementary Figure 2 :

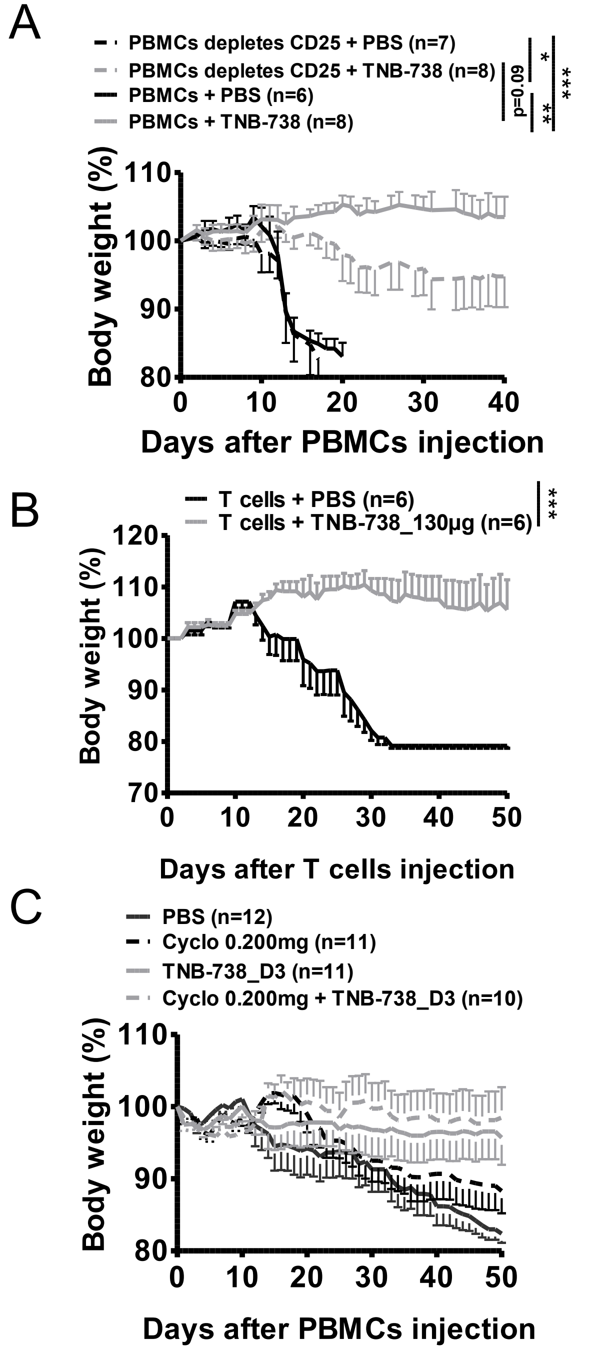

A. NSG mice were irradiated and 24h later received hPBMC (solid lines) intravenous (i.v) (day 0) or hPBMC depleted of CD25+ cells (dotted lines). Animals were then treated with intraperitoneal (i.p) injections of PBS (black lines); TNB-738 at 130µg (gray line) day 0 to day 18 twice a week. Mice were sacrificed when they lost 10-20% of initial body weight (IBW). Graph represents survival curve, right graph shows mean of body weight in each group as a percentage of IBW, (Data from 3 independent experiments). **B**. NSG mice were irradiated and 24h later received purified human T cells intravenous (i.v) (day 0). Animals were then treated with intraperitoneal (i.p) injections of PBS (black line); or TNB-738 at 130µg (gray line) day 0 to day 18 twice a week. Mice were sacrificed when they lost 20% of IBW. The left graph represents survival curve, the right graph shows mean of body weight in each group as a percentage of IBW, (Data from 2 independent experiments). **C**. NSG mice were irradiated and 24h later received hPBMCs i.v (day 0). Animals were treated from D3 to D21 with intraperitoneal (i.p) injections of PBS (black line); TNB-738 130 µg (gray line), Cyclosporine 0.200 mg (dotted black line) or a combination of cyclosporine and TNB-738 (dotted black line). Mice were sacrificed when they lost 10-20% of initial body weight (IBW). Left graph represents survival curve, right graph represents mean of body weight in each group as a percentage of IBW. * $p<0.05$ ** $p<0.01$ *** $p<0.001$.

Supplementary Figure 3 :

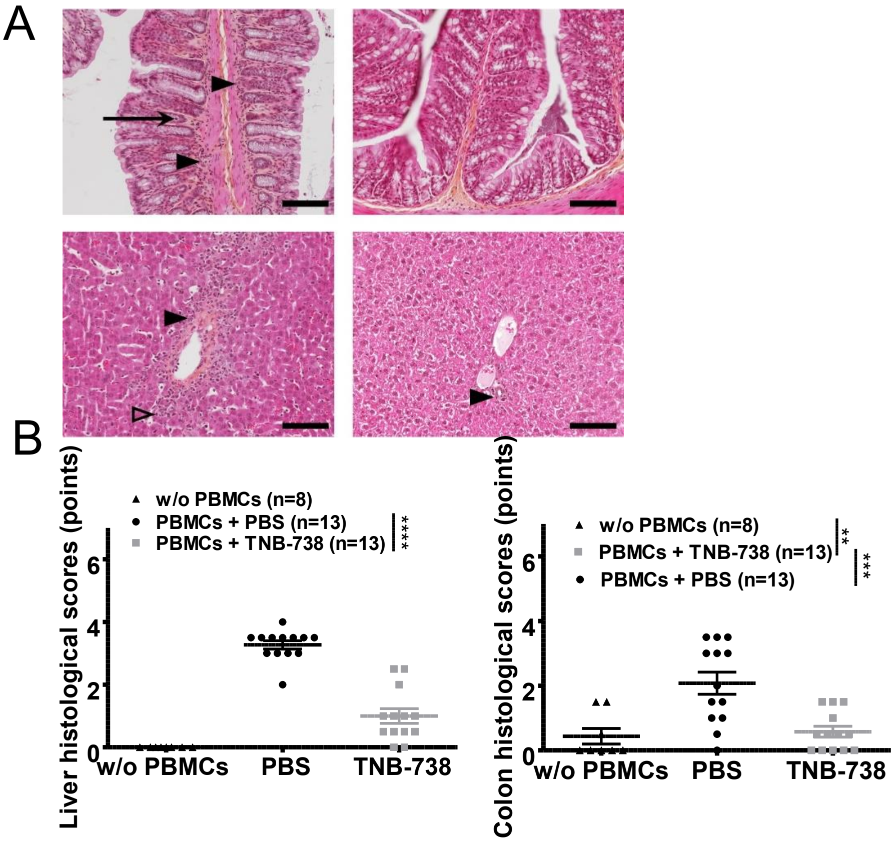

A. Histopathological significant presentation. In colon (Upper panel), focal infiltration of the lamina propria was present (arrowhead). A few necrotic cells were also observed in rare crypts (arrow). In liver (Lower panel), perivascular cuffing was mainly prominent in portal areas and infiltrated surrounding liver parenchyma (black arrowhead). Some isolated necrotic hepatocytes were also present (open arrow). Hemalun-Eosin-Saffron staining. Bar=100µm. **B**. Histological score of liver (left graph) or colon (right graph) in PBS (black point) or TNB-738 (gray scare) NSG that did not receive hPBMC (black triangle). * p<0.05 ** p<0.01 ***p< 0.001 ****p<0.0001.

Supplementary Table 1. Gene expression analysis summary.

Comparison	Group	Genes with Padj <0.01	Genes with Padj <0.01 and logc2FC > 1	Genes with Padj <0.01 and logc2FC < -1
1	TNB-738 vs. PBS	573	241	191
2	TNB-738 vs. Control	329	271	35
