# Supplementary material for: Inhibition of CD38 enzyme activity on engrafted human immune cells enhances NAD+ metabolism and inhibits inflammation in an in-vivo model of xeno-GvHD
Source: Front Immunol. 2025 Oct 13;16:1640611. doi: 10.3389/fimmu.2025.1640611 (PMC12555386; doi:10.3389/fimmu.2025.1640611)

## Slide 1
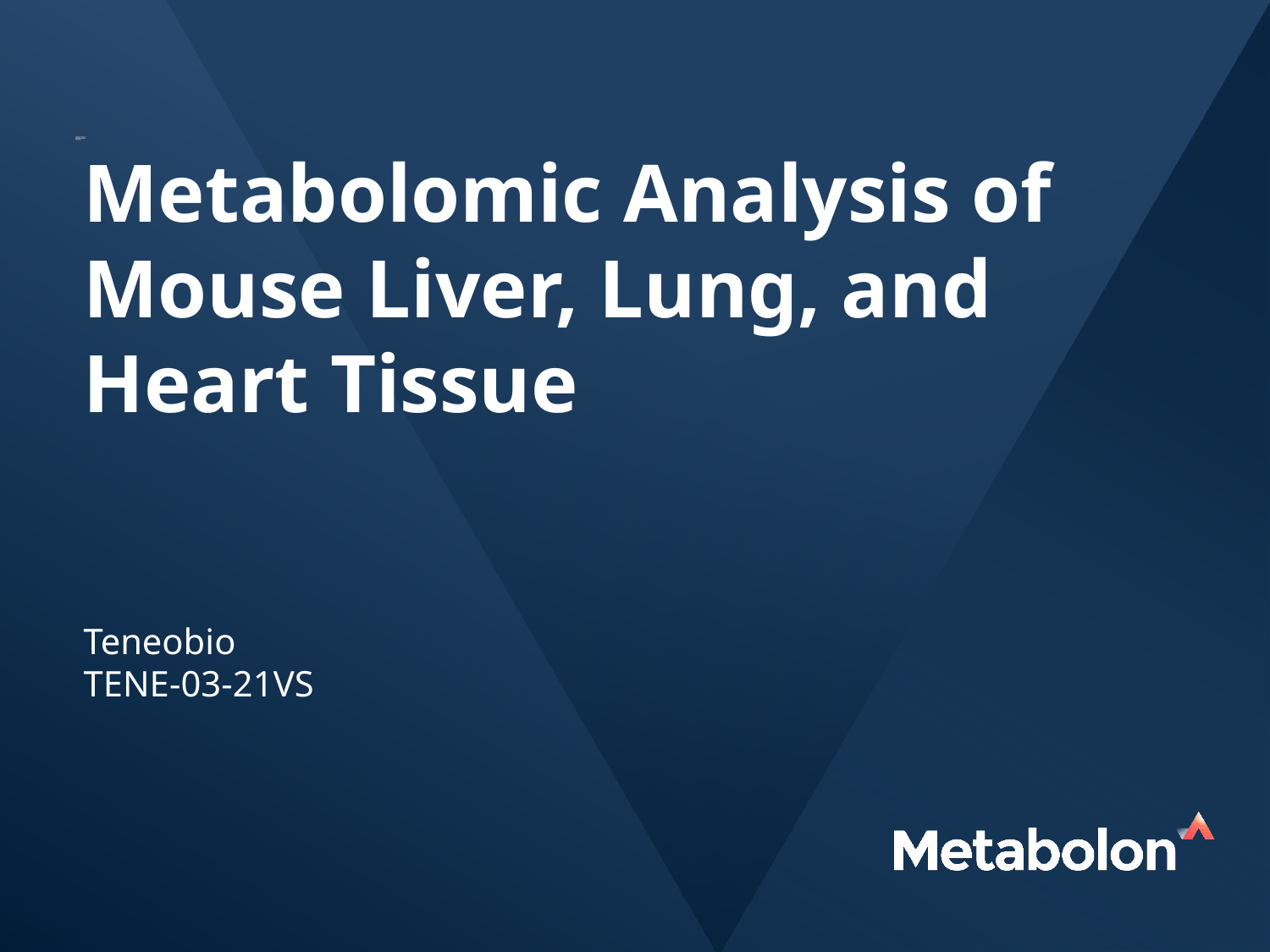

Metabolomic Analysis of Mouse Liver, Lung, and Heart Tissue
Teneobio
TENE-03-21VS

## Slide 2
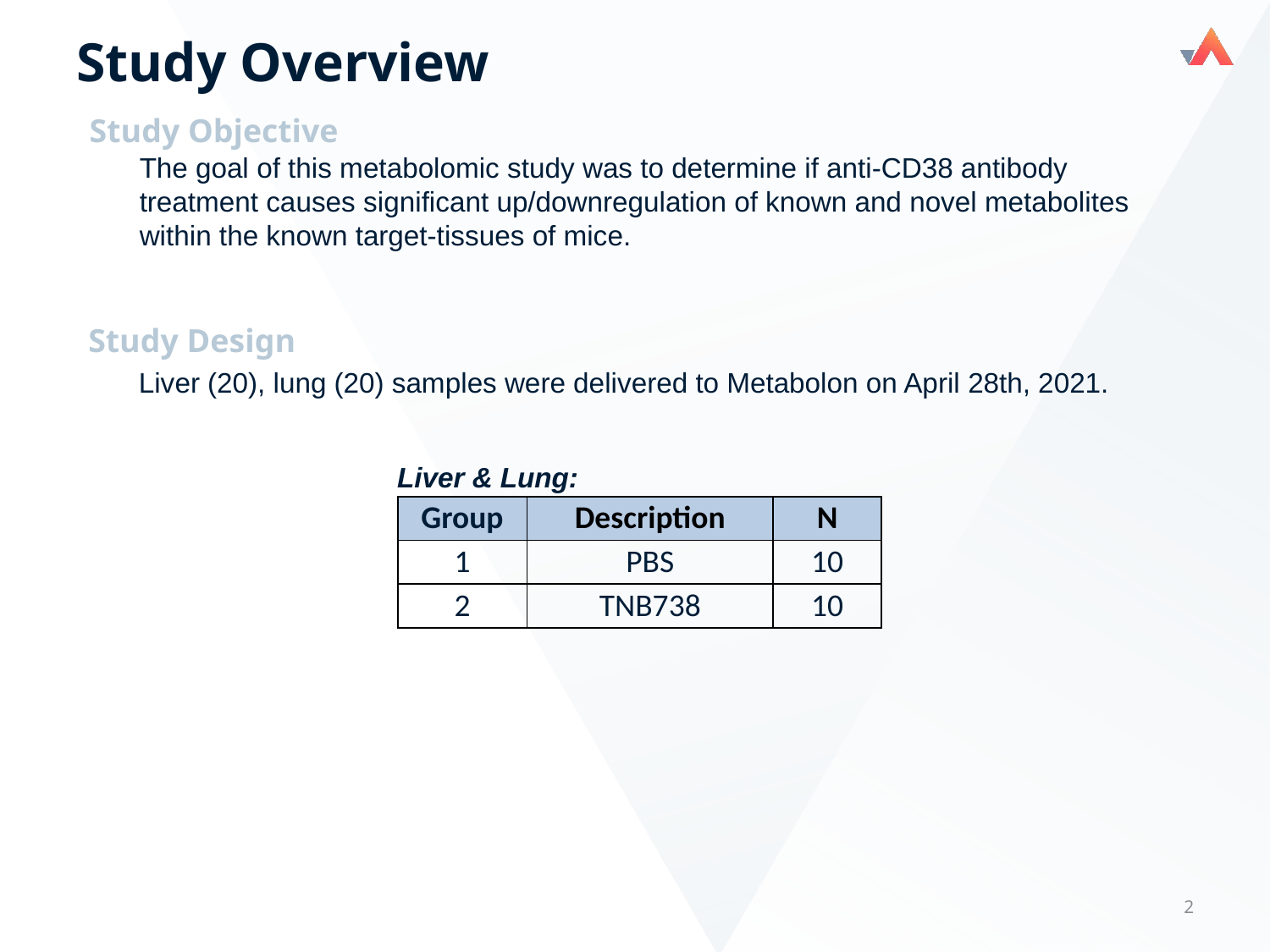

# Study Overview
Study Objective
The goal of this metabolomic study was to determine if anti-CD38 antibody treatment causes significant up/downregulation of known and novel metabolites within the known target-tissues of mice.
Study Design
Liver (20), lung (20) samples were delivered to Metabolon on April 28th, 2021.
Liver & Lung:
| Group | Description | N |
| --- | --- | --- |
| 1 | PBS | 10 |
| 2 | TNB738 | 10 |
2

## Slide 3
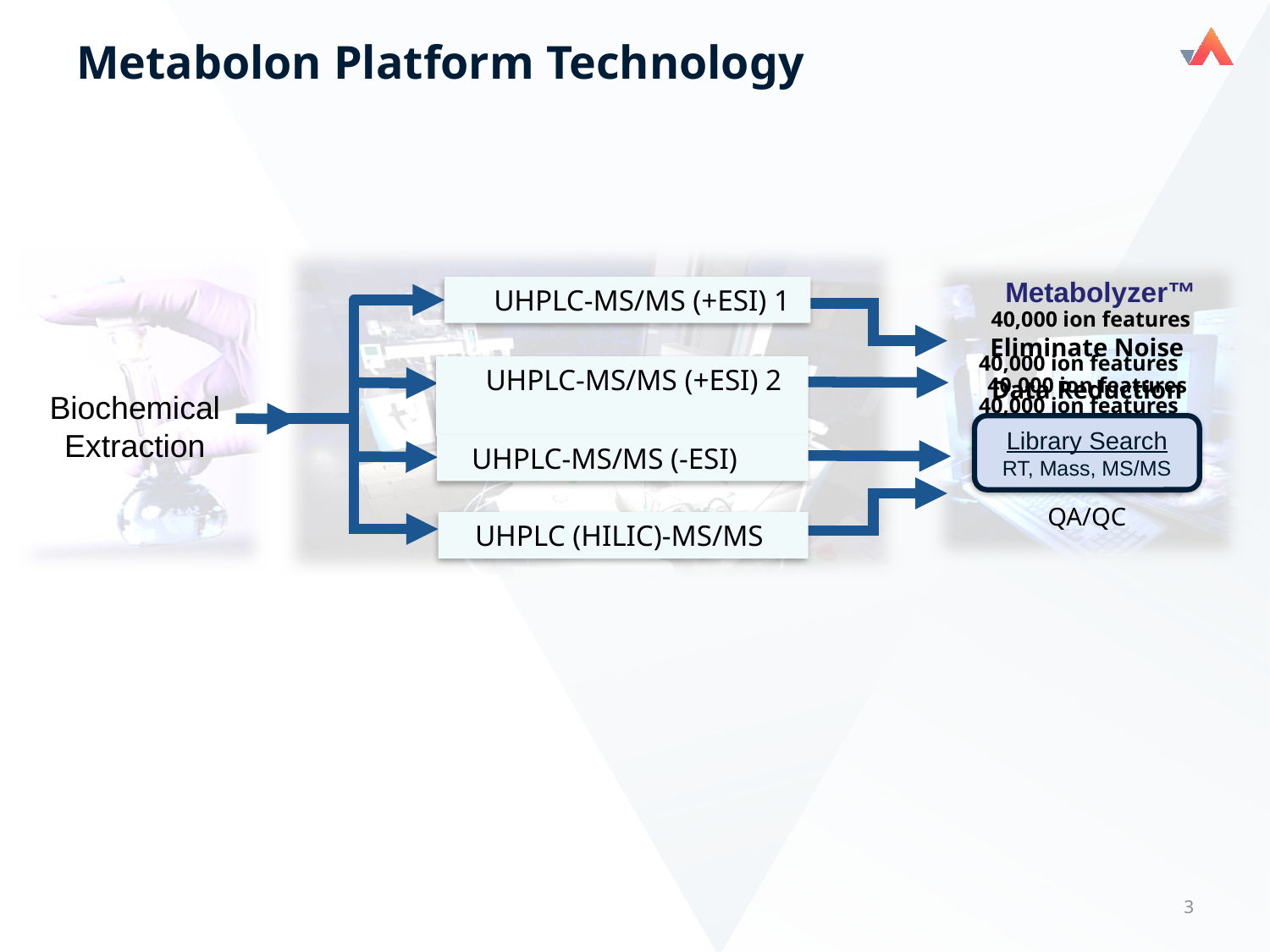

# Metabolon Platform Technology
Metabolyzer™
 UHPLC-MS/MS (+ESI) 1
40,000 ion features
40,000 ion features
40,000 ion features
40,000 ion features
Eliminate Noise
Data Reduction
Library Search
RT, Mass, MS/MS
QA/QC
 UHPLC-MS/MS (+ESI) 2
Biochemical
Extraction
 UHPLC-MS/MS (-ESI)
UHPLC (HILIC)-MS/MS
3

## Slide 4
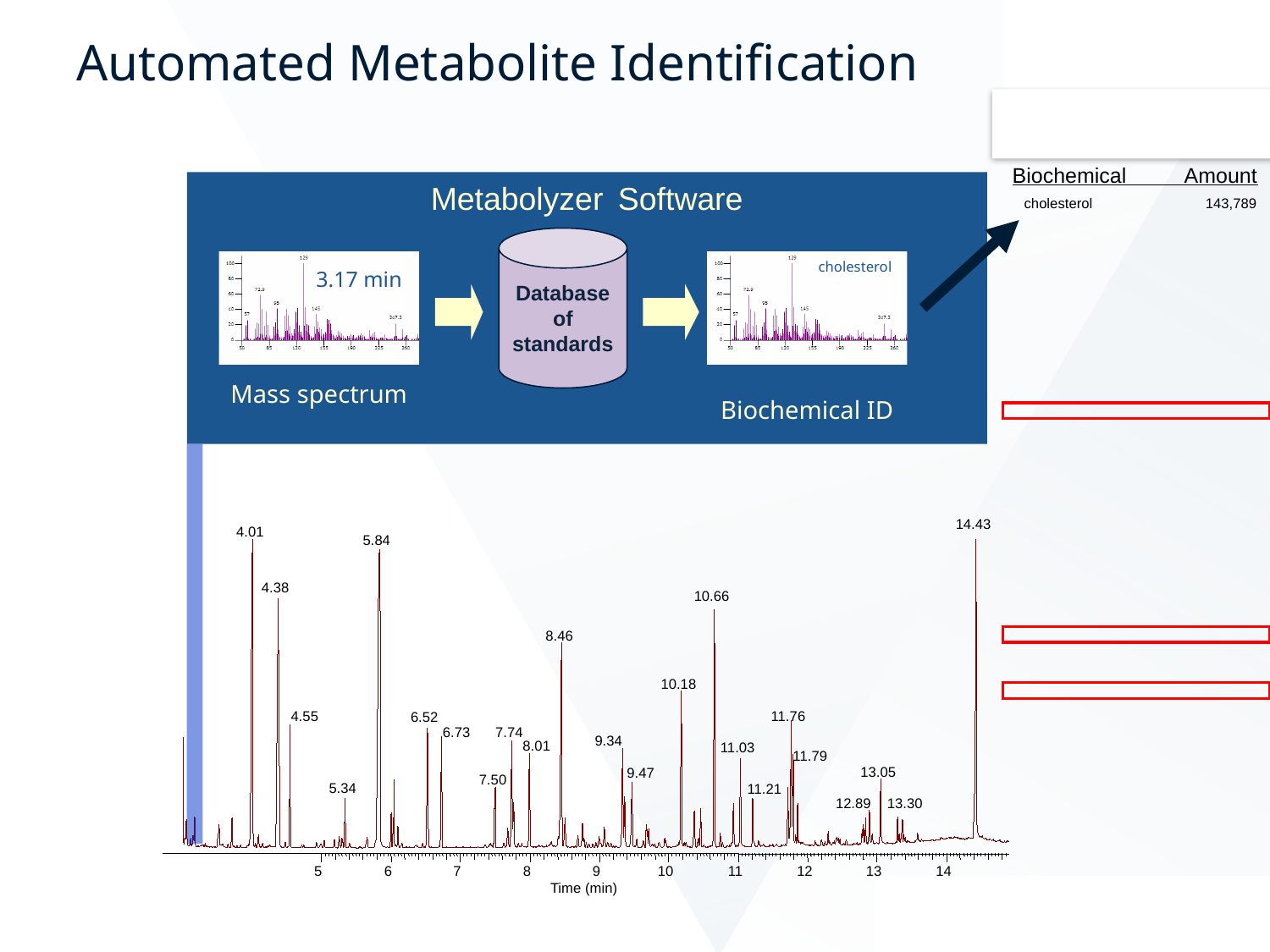

# Automated Metabolite Identification
Biochemical Amount
Metabolyzer Software
cholesterol
143,789
Database
of
standards
cholesterol
Biochemical ID
3.17 min
Mass spectrum
14.43
4.01
5.84
4.38
10.66
8.46
10.18
4.55
11.76
6.52
6.73
7.74
9.34
8.01
11.03
11.79
13.05
9.47
7.50
5.34
11.21
12.89
13.30
5
6
7
8
9
10
11
12
13
14
Time (min)
4

## Slide 5
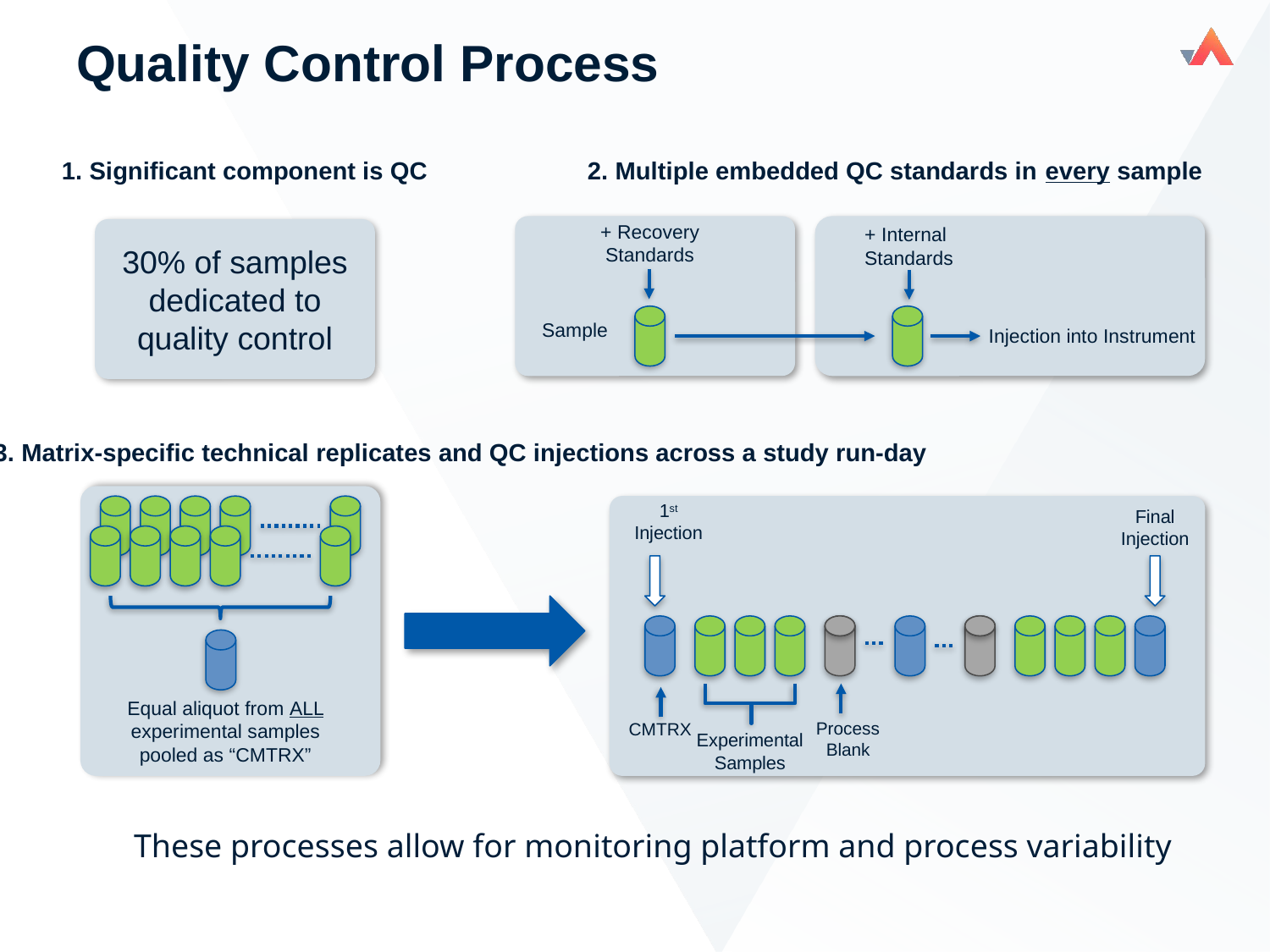

# Quality Control Process
1. Significant component is QC 2. Multiple embedded QC standards in every sample
30% of samples dedicated to quality control
+ Recovery Standards
+ Internal Standards
Sample
Injection into Instrument
3. Matrix-specific technical replicates and QC injections across a study run-day
1st Injection
Final
Injection
Equal aliquot from ALL experimental samples pooled as “CMTRX”
CMTRX
Process Blank
Experimental Samples
These processes allow for monitoring platform and process variability

## Slide 6
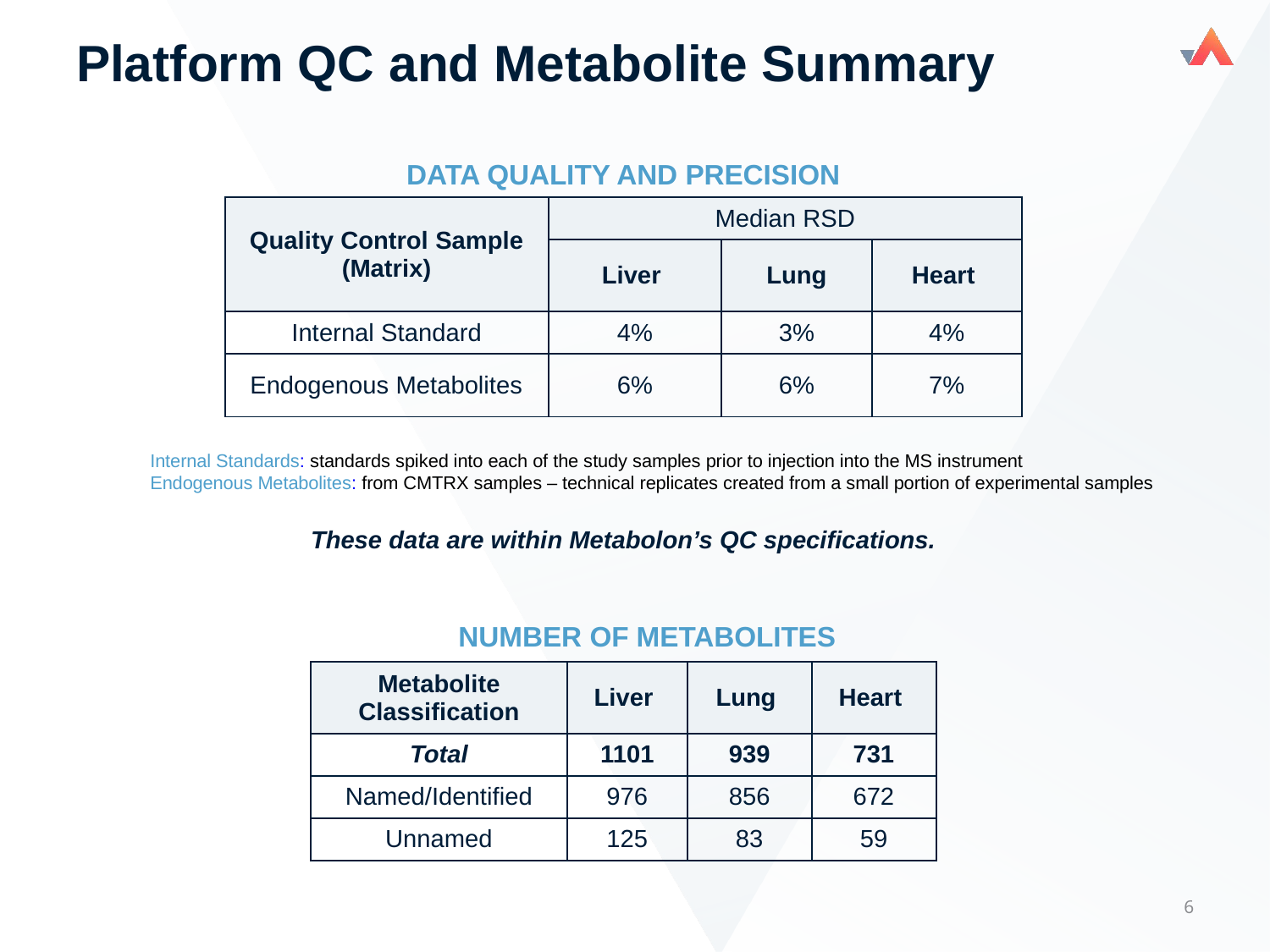

# Platform QC and Metabolite Summary
Data Quality and Precision
| Quality Control Sample (Matrix) | Median RSD | | |
| --- | --- | --- | --- |
| | Liver | Lung | Heart |
| Internal Standard | 4% | 3% | 4% |
| Endogenous Metabolites | 6% | 6% | 7% |
Internal Standards: standards spiked into each of the study samples prior to injection into the MS instrument
Endogenous Metabolites: from CMTRX samples – technical replicates created from a small portion of experimental samples
These data are within Metabolon’s QC specifications.
Number of Metabolites
| Metabolite Classification | Liver | Lung | Heart |
| --- | --- | --- | --- |
| Total | 1101 | 939 | 731 |
| Named/Identified | 976 | 856 | 672 |
| Unnamed | 125 | 83 | 59 |
6

## Slide 7
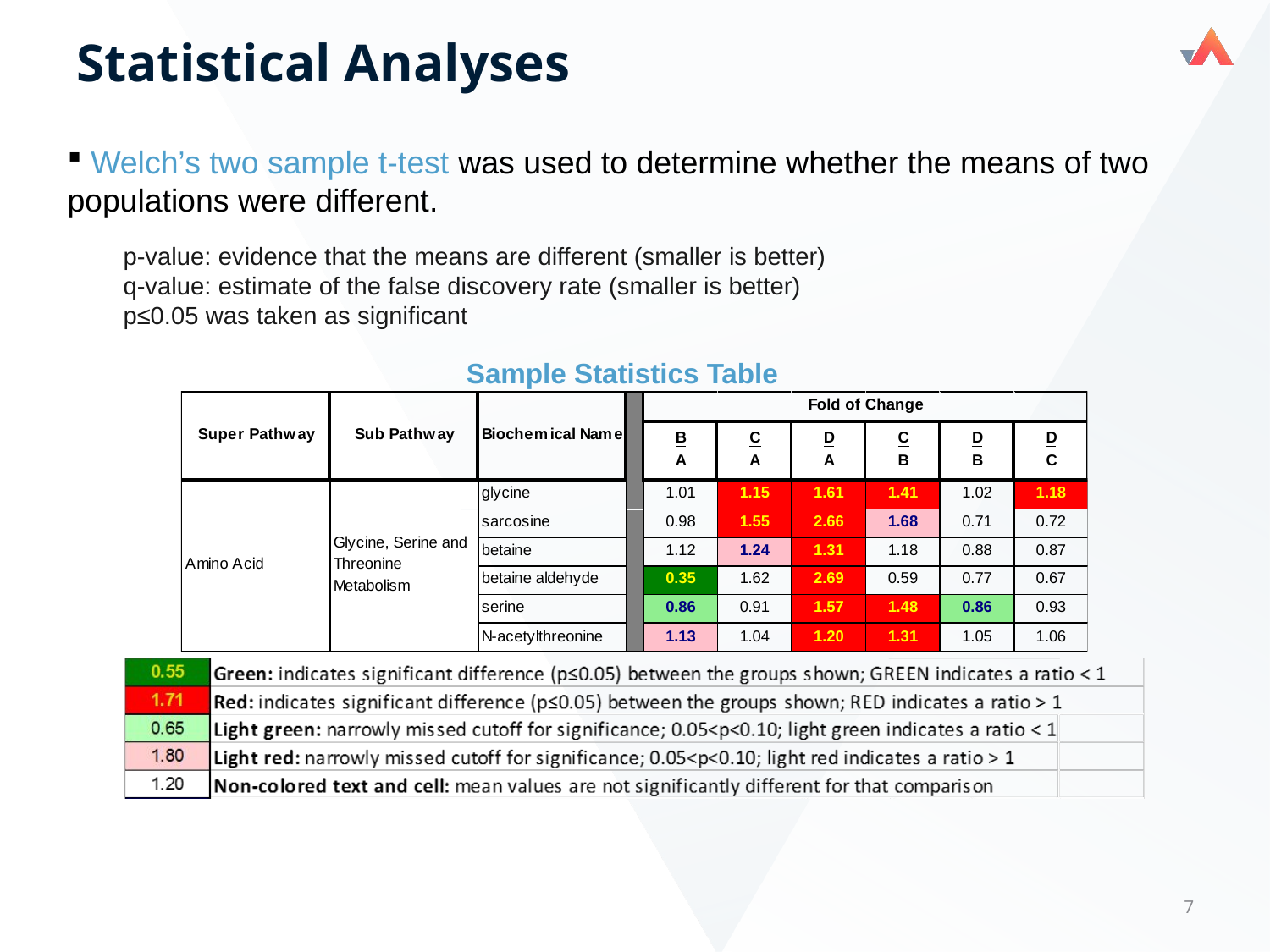

# Statistical Analyses
 Welch’s two sample t-test was used to determine whether the means of two populations were different.
 p-value: evidence that the means are different (smaller is better)
 q-value: estimate of the false discovery rate (smaller is better)
 p≤0.05 was taken as significant
Sample Statistics Table
7

## Slide 8
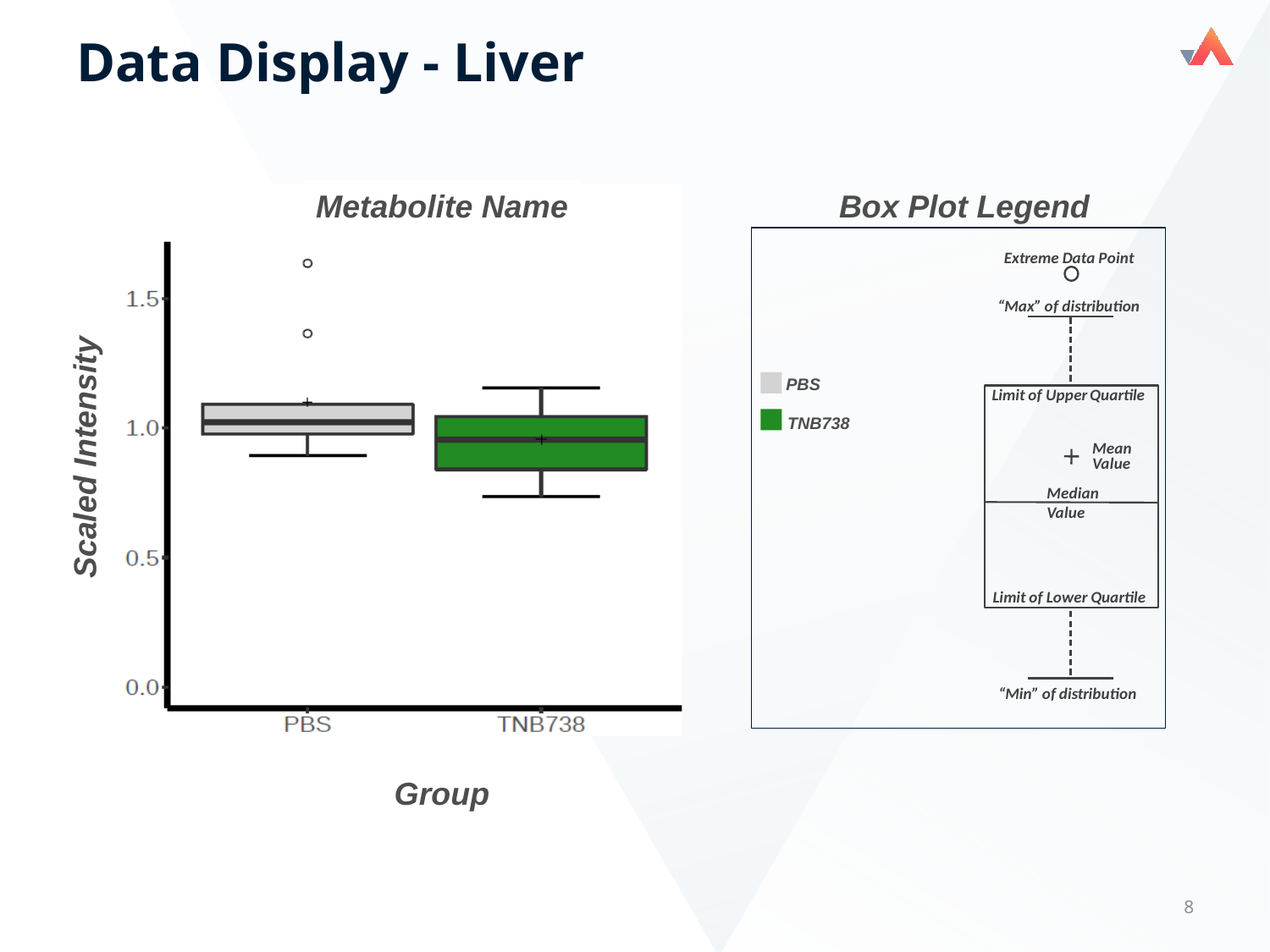

# Data Display - Liver
Metabolite Name
Scaled Intensity
Group
Box Plot Legend
PBS
TNB738
8

## Slide 9
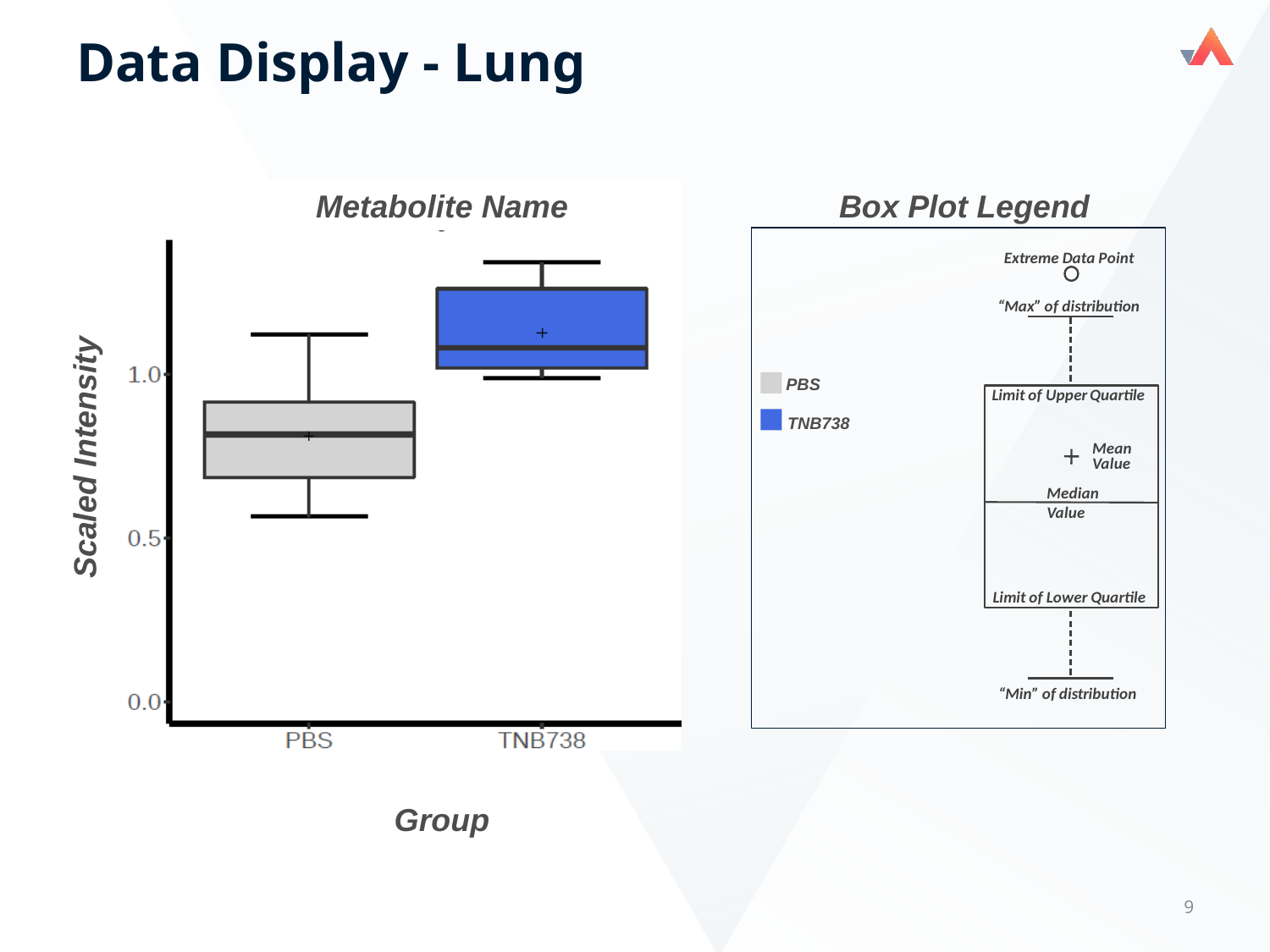

# Data Display - Lung
Metabolite Name
Scaled Intensity
Group
Box Plot Legend
PBS
TNB738
9

## Slide 10
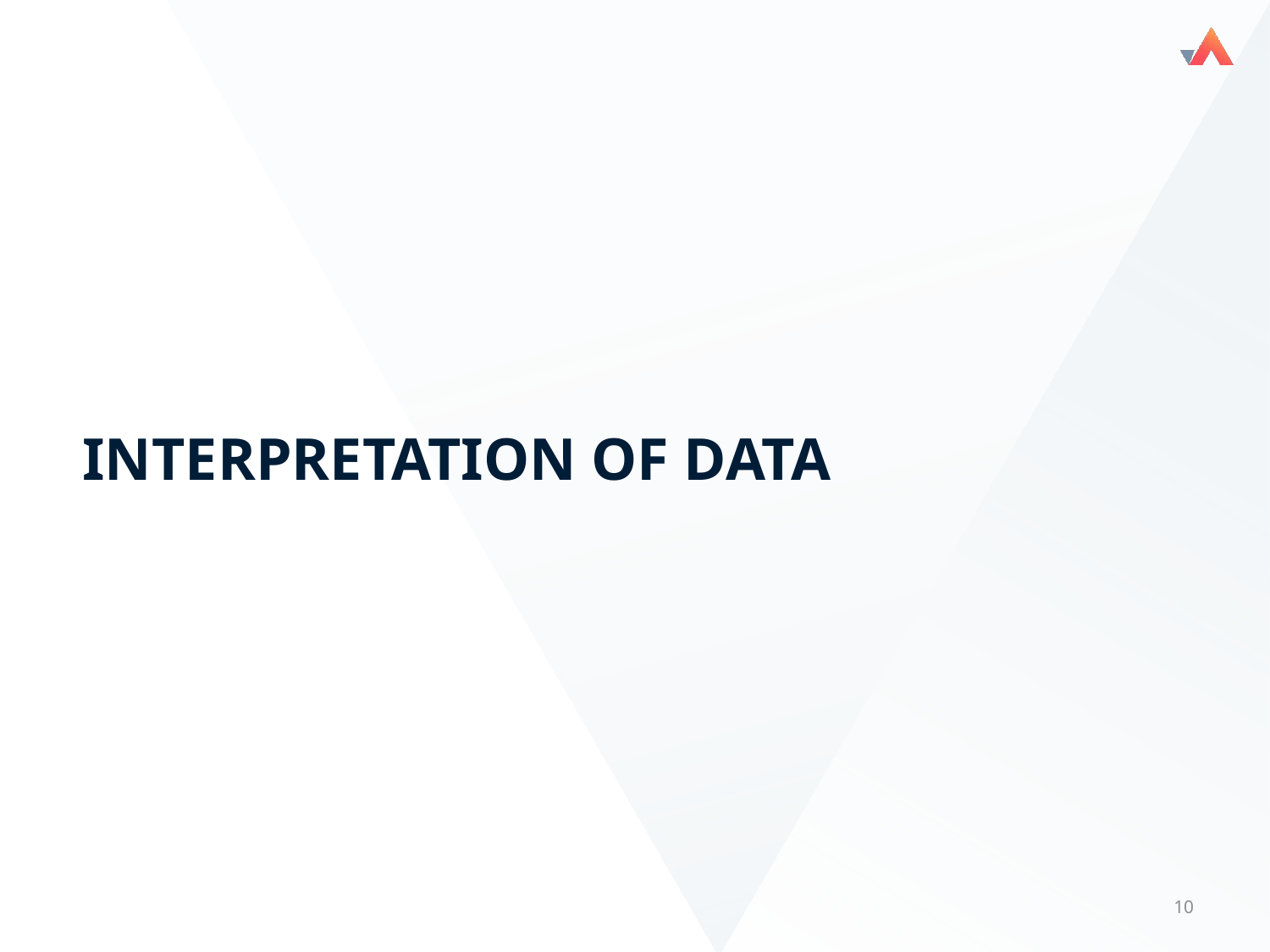

Interpretation of data
10

## Slide 11
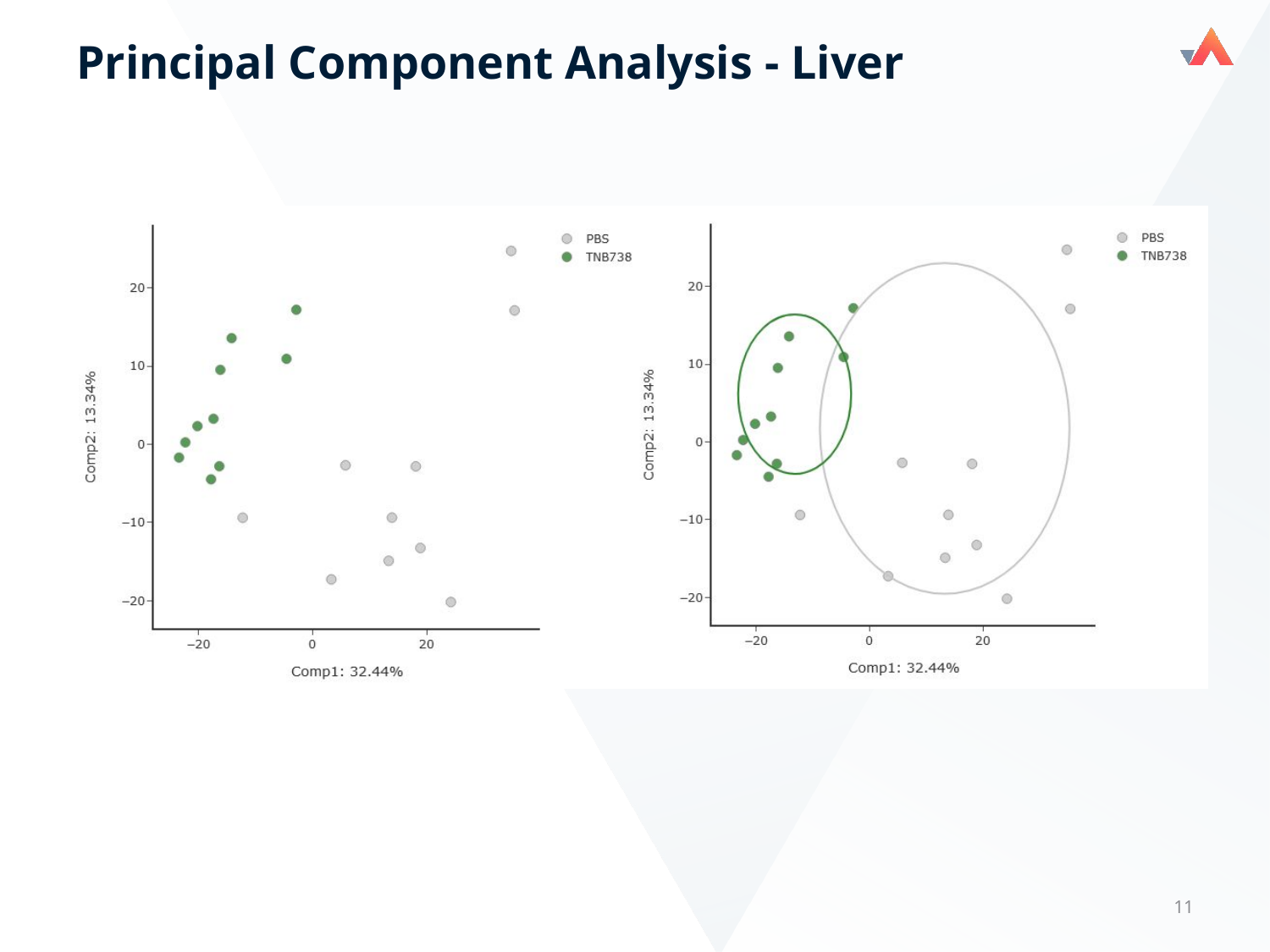

# Principal Component Analysis - Liver
11

## Slide 12
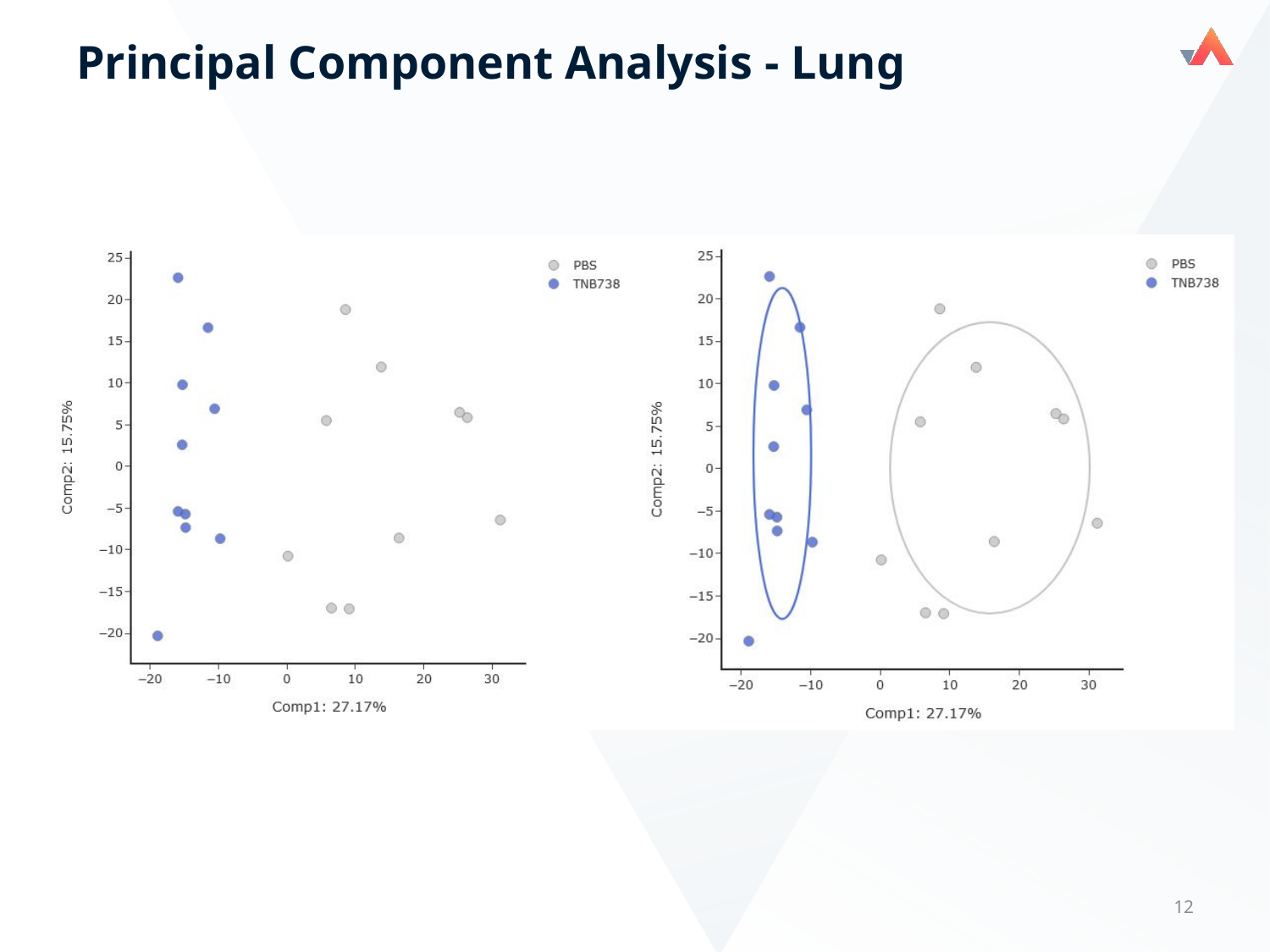

# Principal Component Analysis - Lung
12

## Slide 13
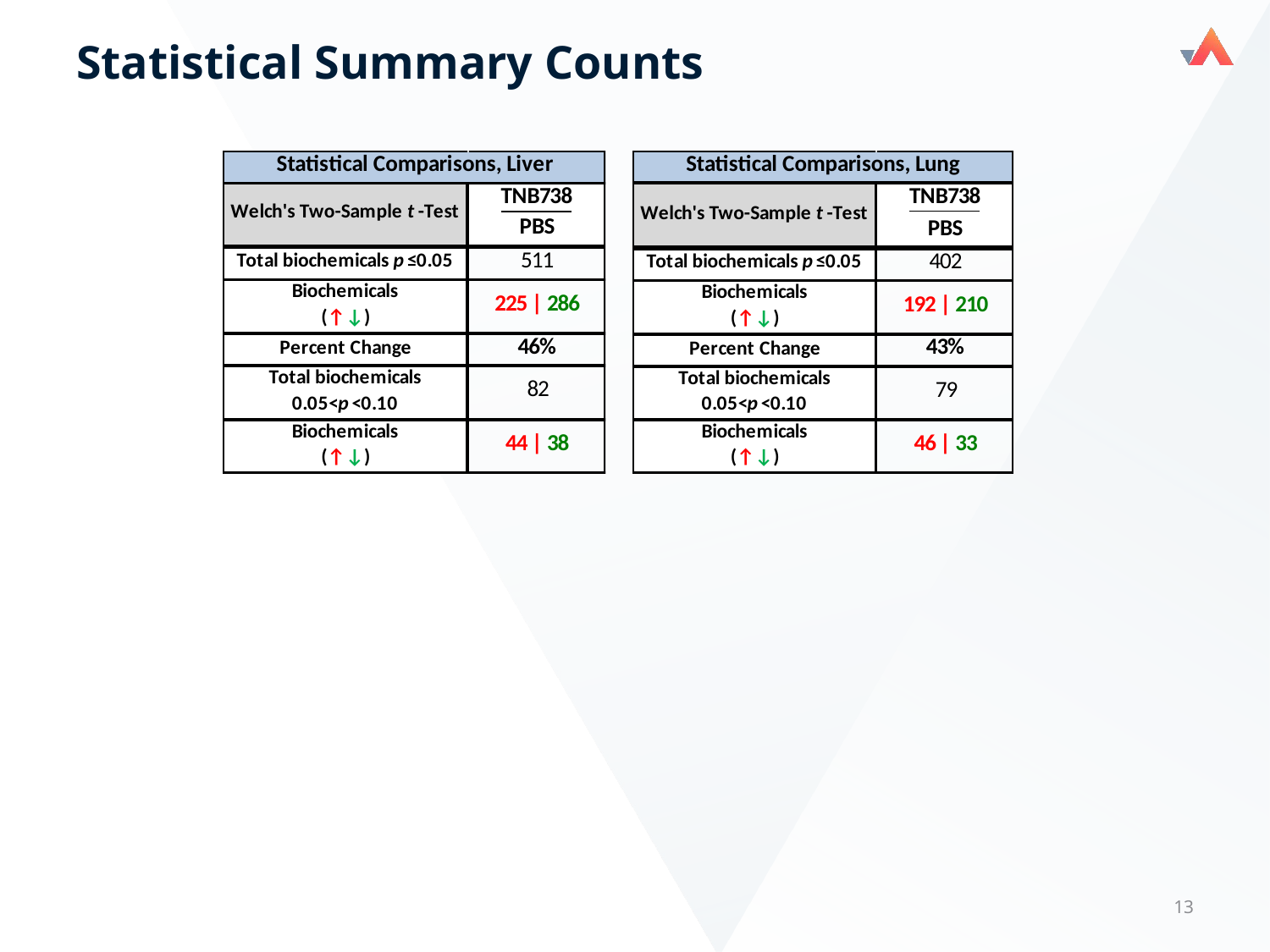

# Statistical Summary Counts
13

## Slide 14
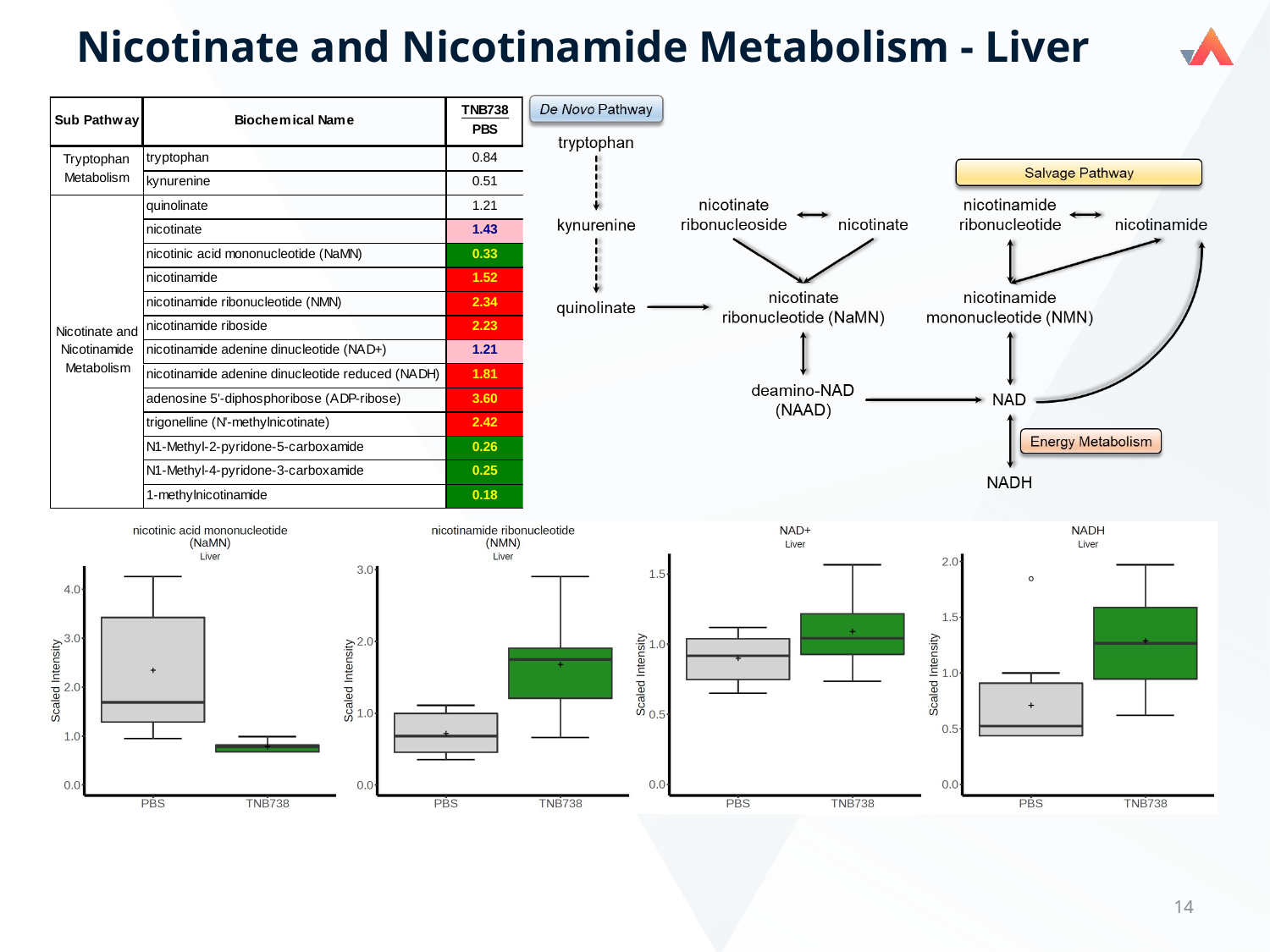

# Nicotinate and Nicotinamide Metabolism - Liver
14

## Slide 15
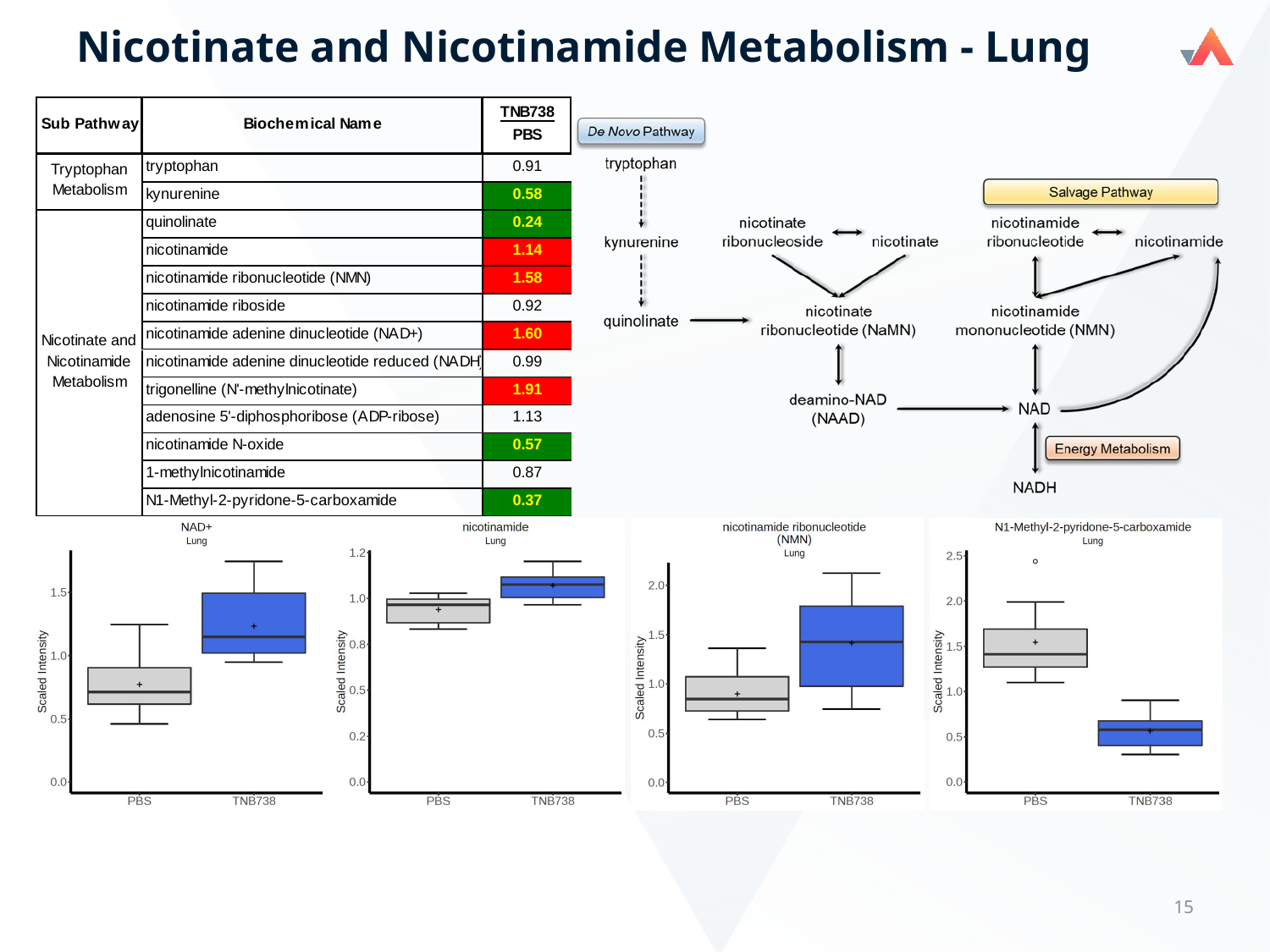

# Nicotinate and Nicotinamide Metabolism - Lung
15

## Slide 16
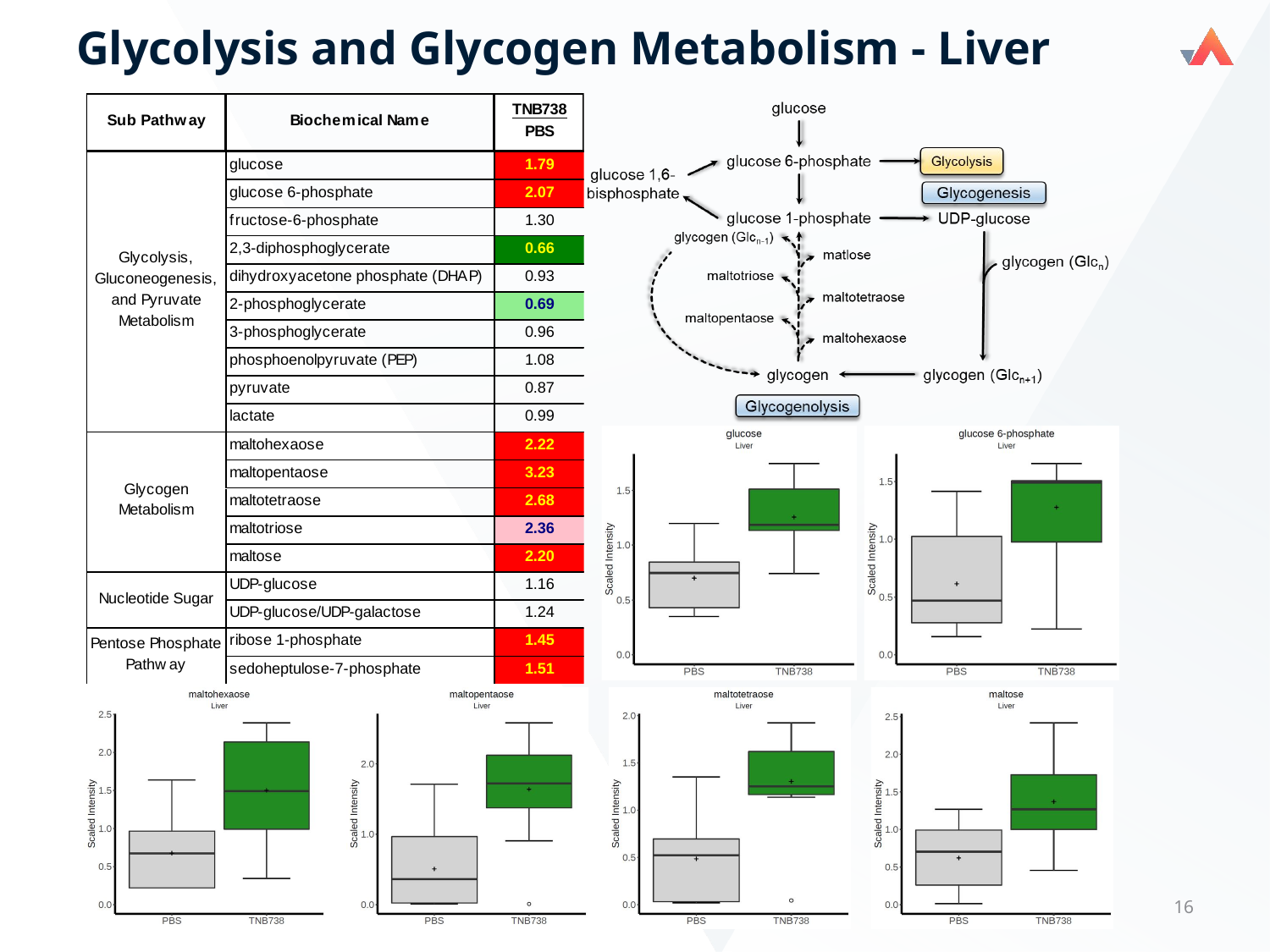

# Glycolysis and Glycogen Metabolism - Liver
16

## Slide 17
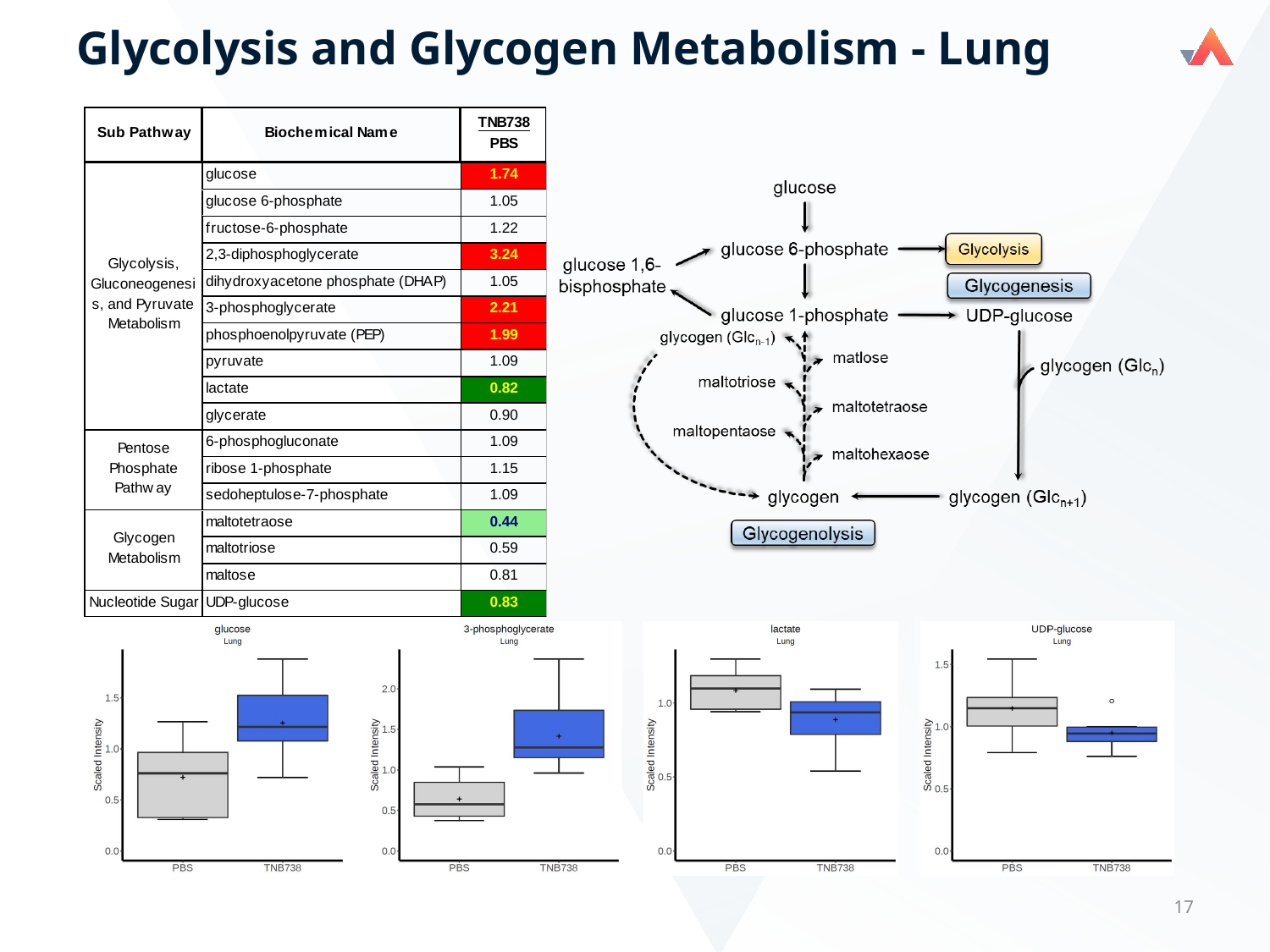

# Glycolysis and Glycogen Metabolism - Lung
17

## Slide 18
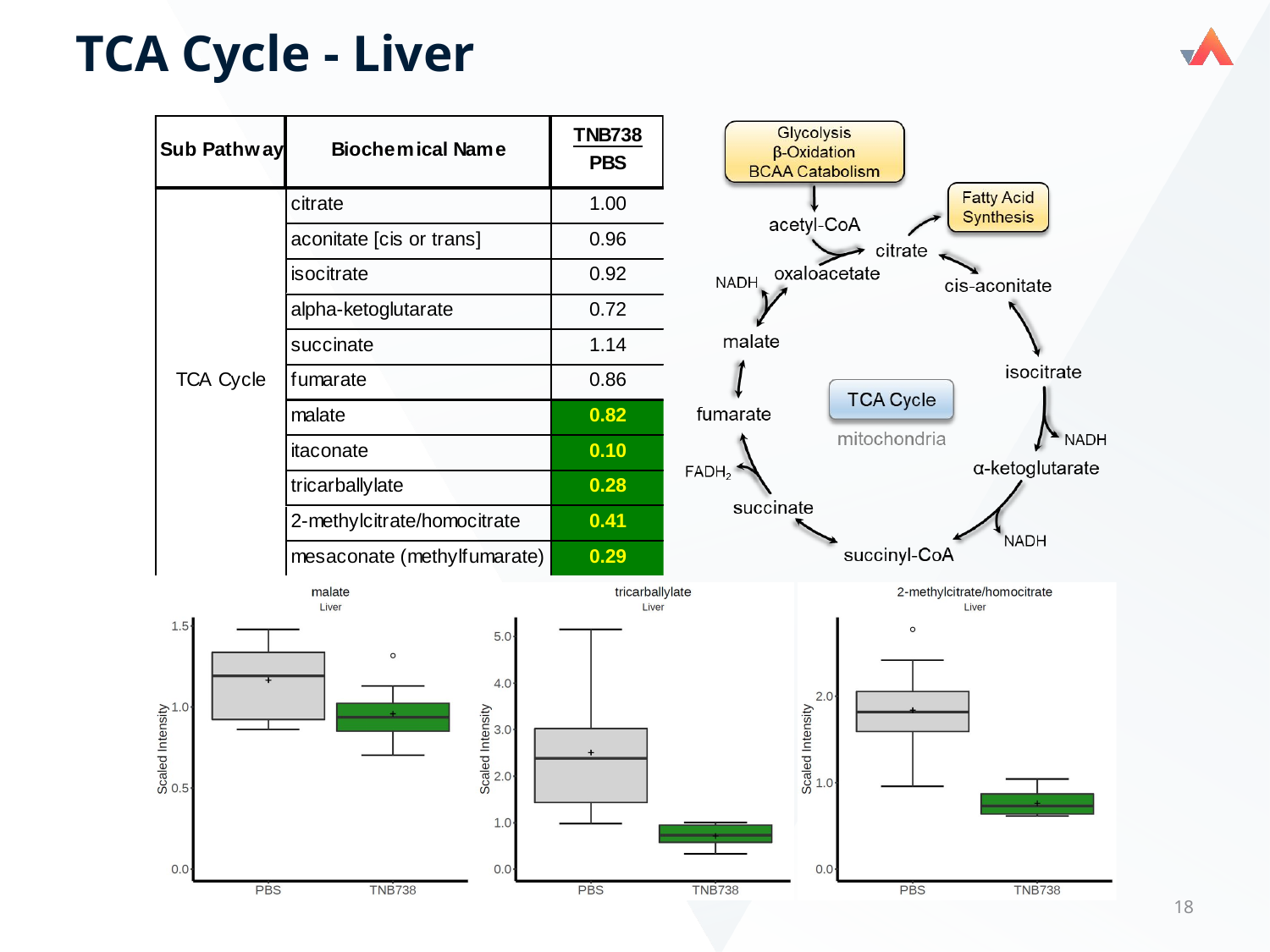

TCA Cycle - Liver
18

## Slide 19
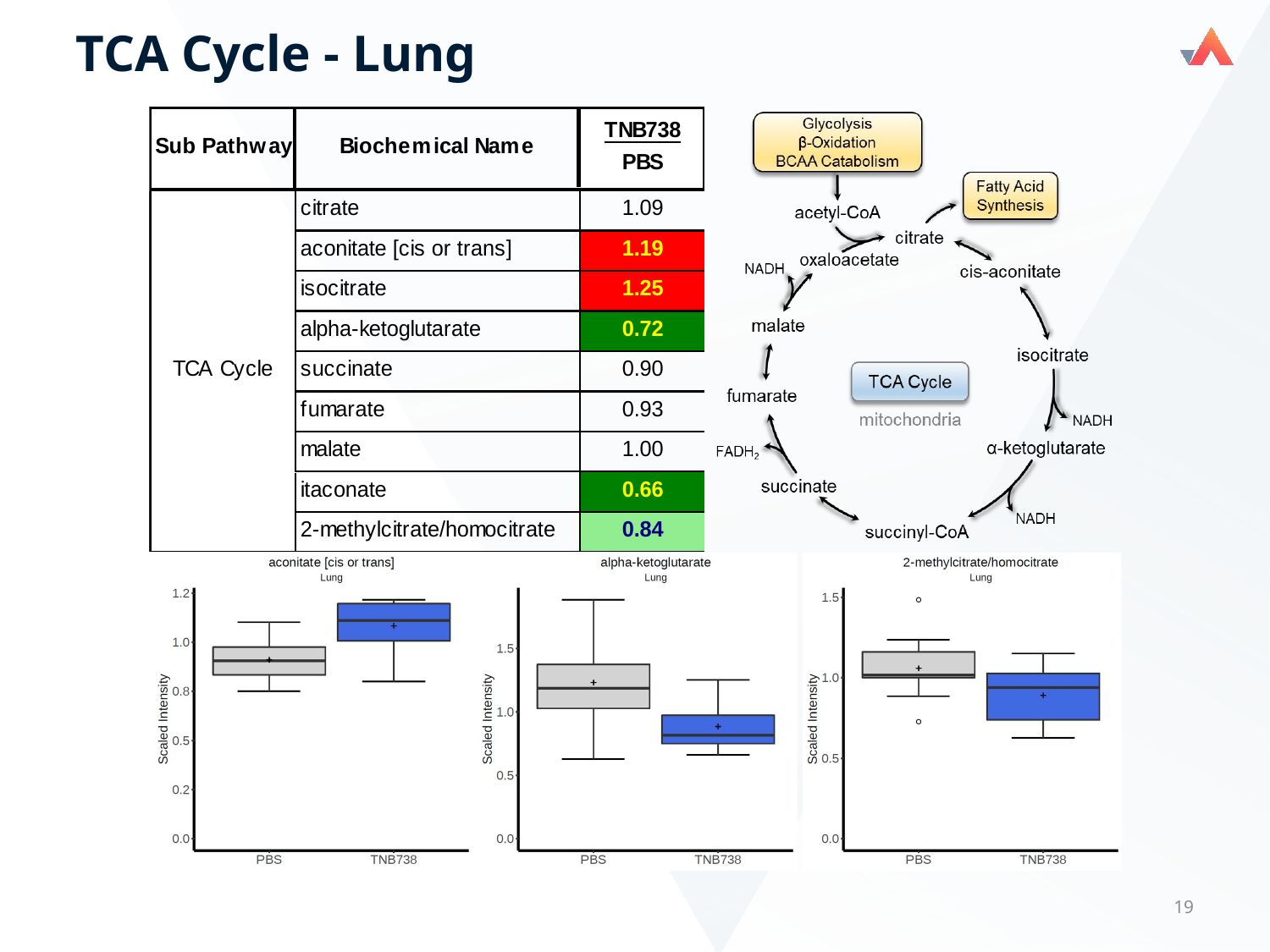

TCA Cycle - Lung
19

## Slide 20
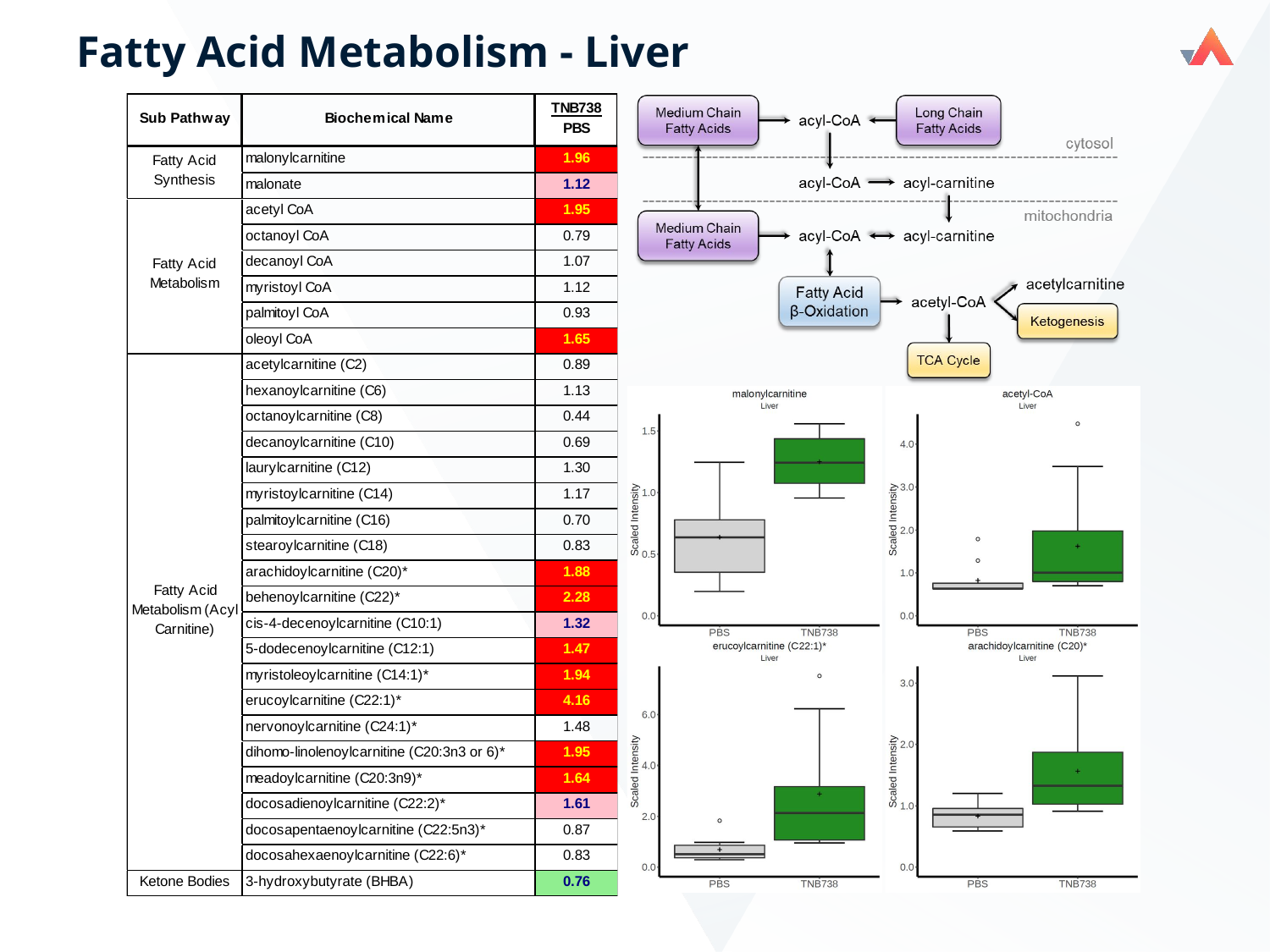

# Fatty Acid Metabolism - Liver

## Slide 21
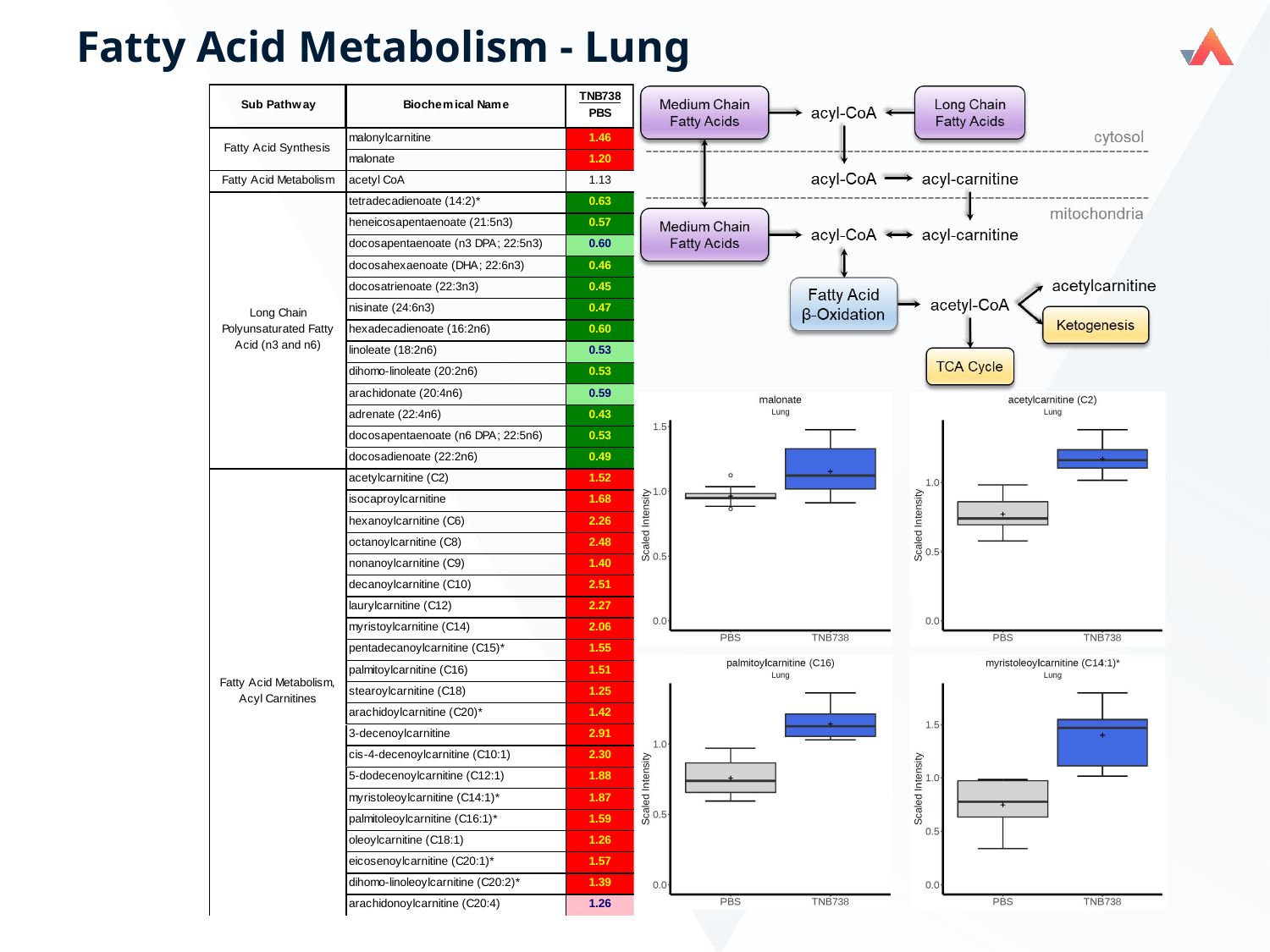

# Fatty Acid Metabolism - Lung

## Slide 22
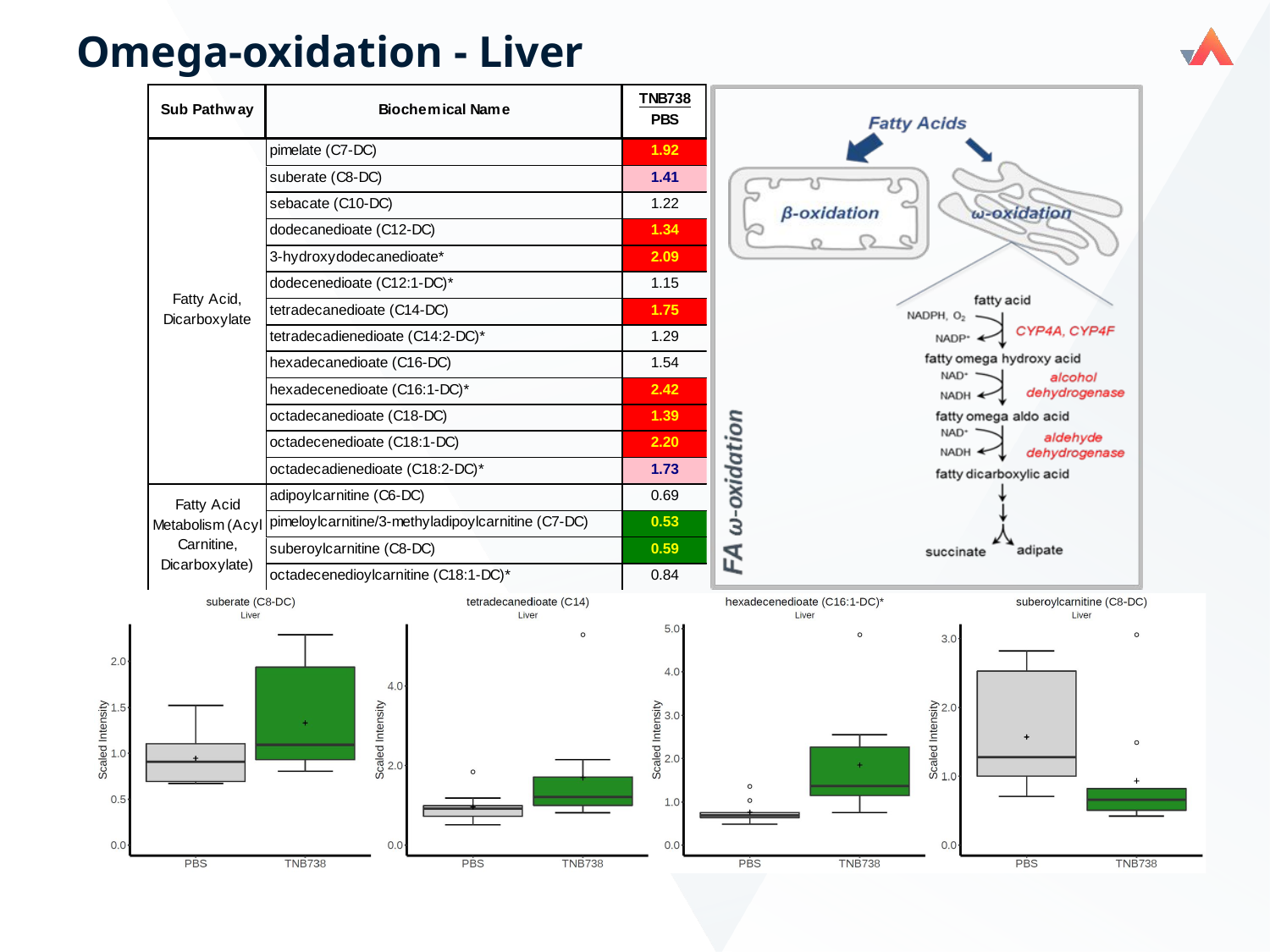

# Omega-oxidation - Liver

## Slide 23
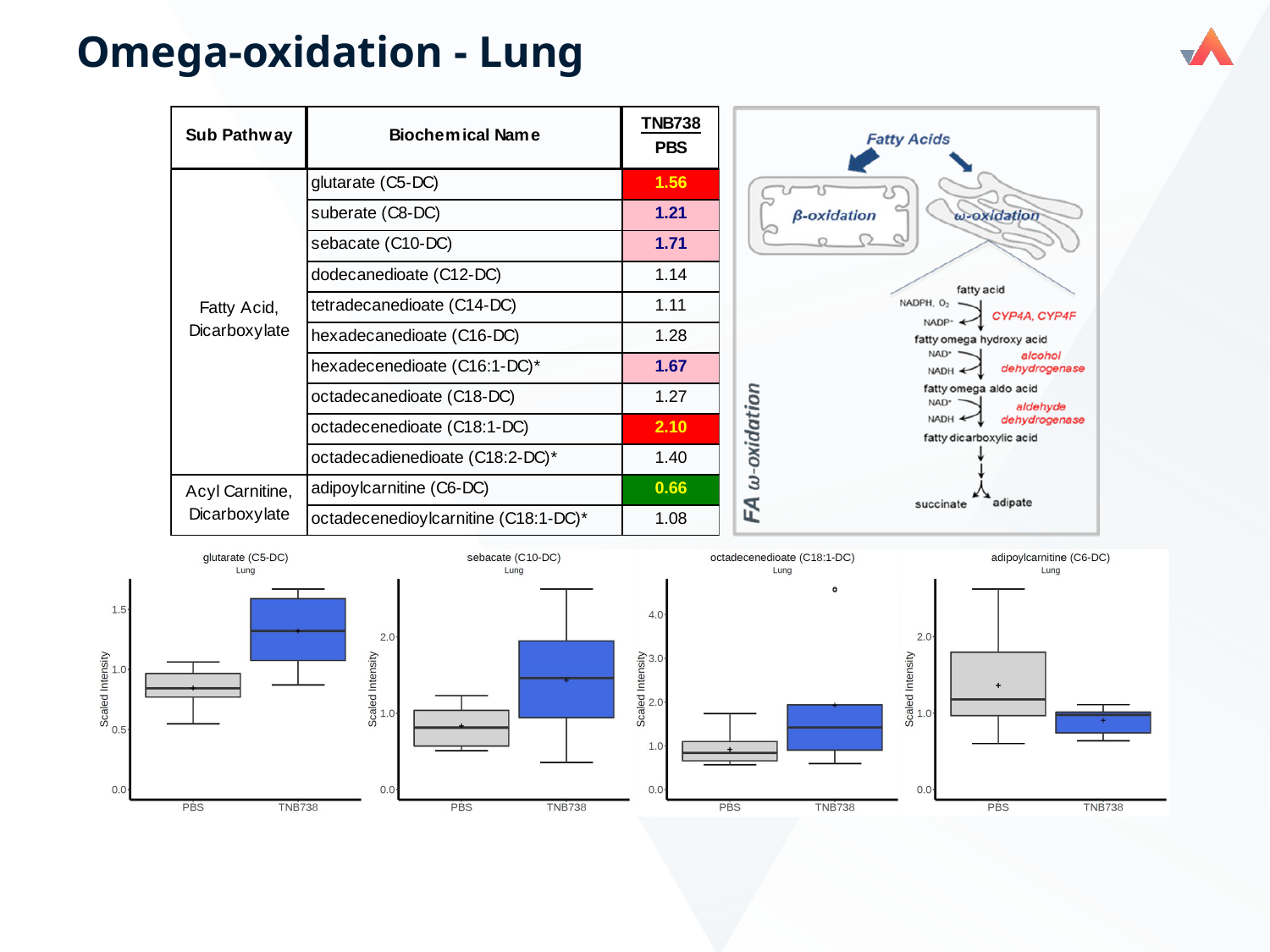

# Omega-oxidation - Lung

## Slide 24
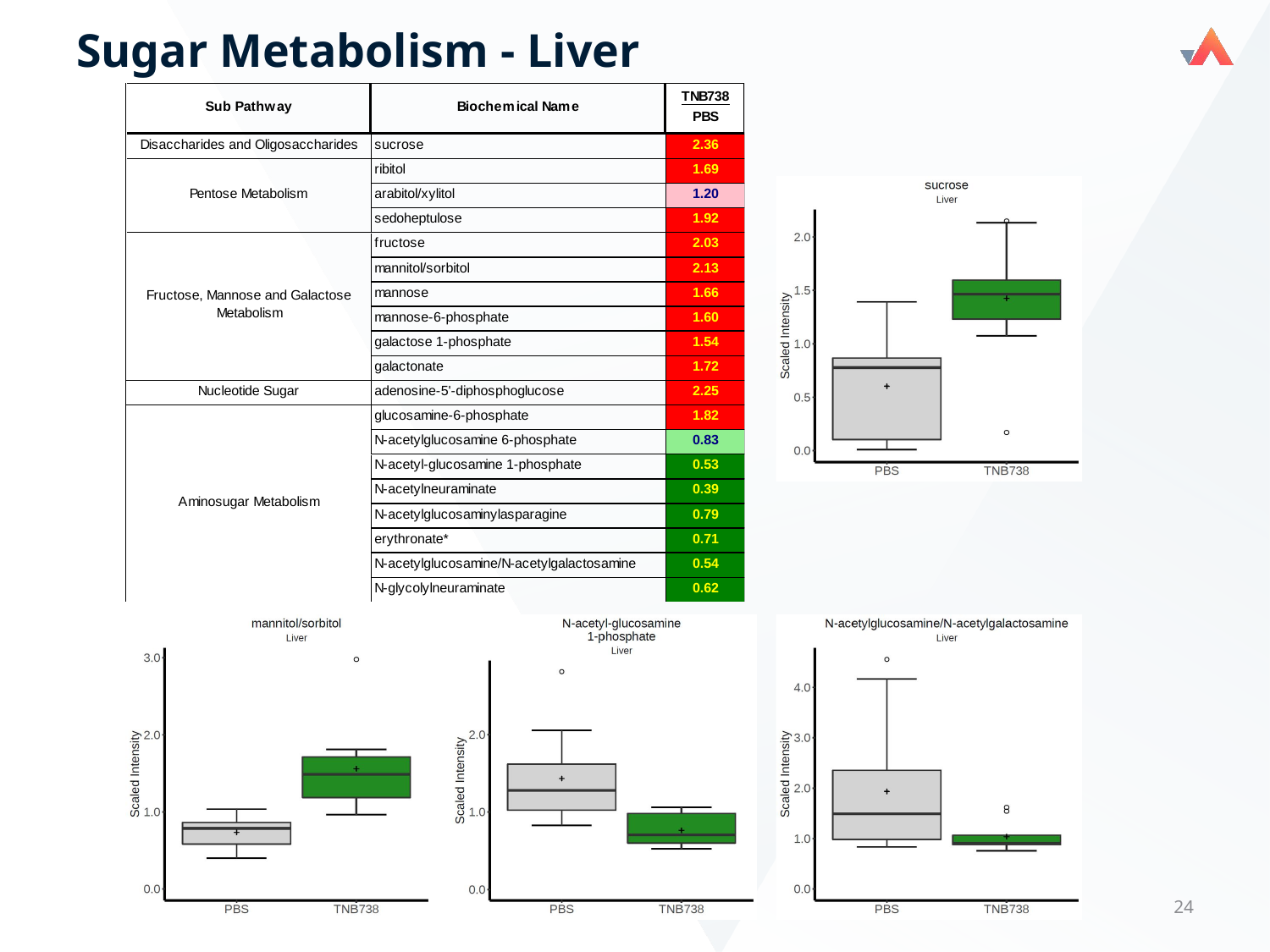

# Sugar Metabolism - Liver
24

## Slide 25
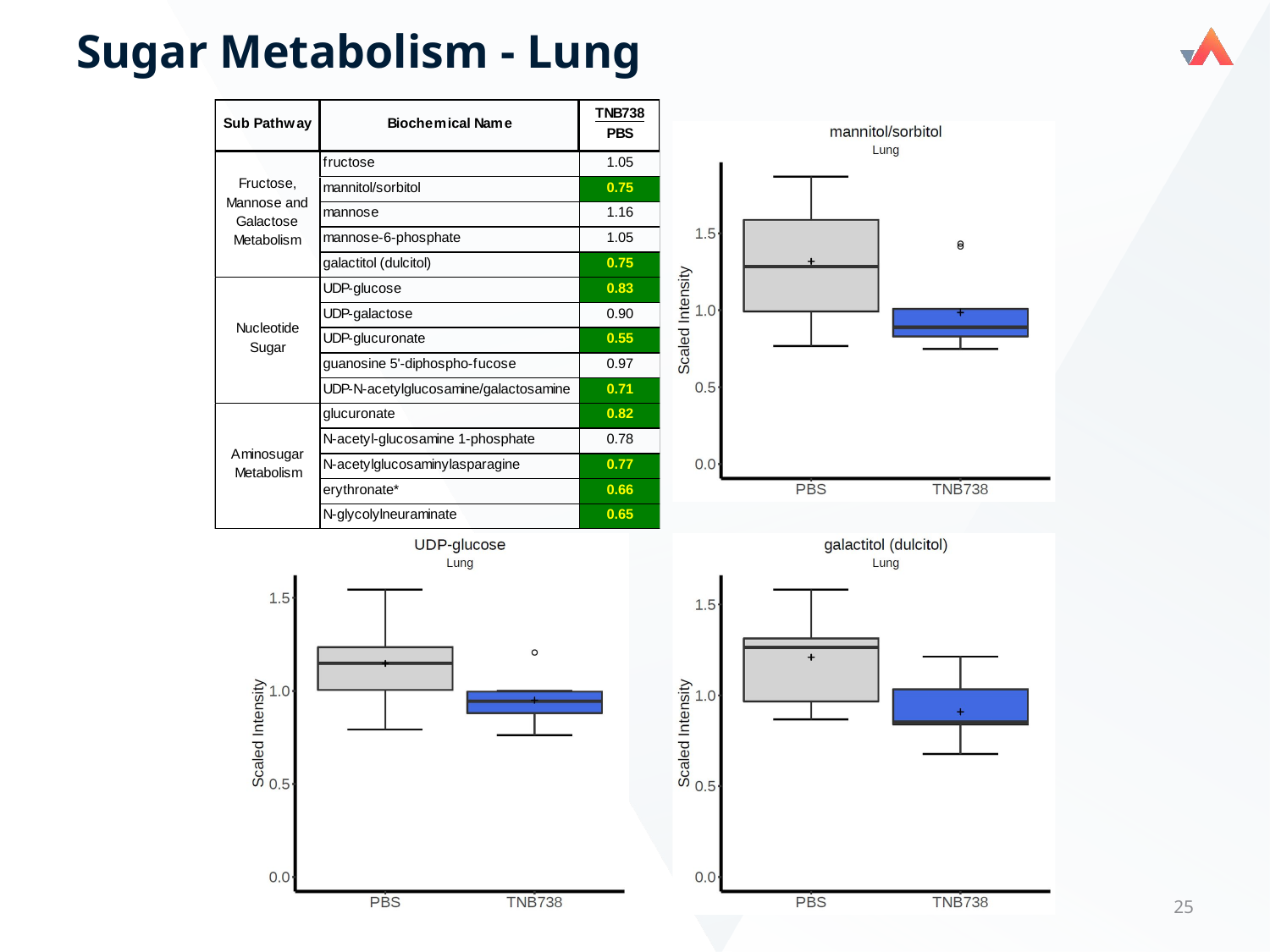

# Sugar Metabolism - Lung
25

## Slide 26
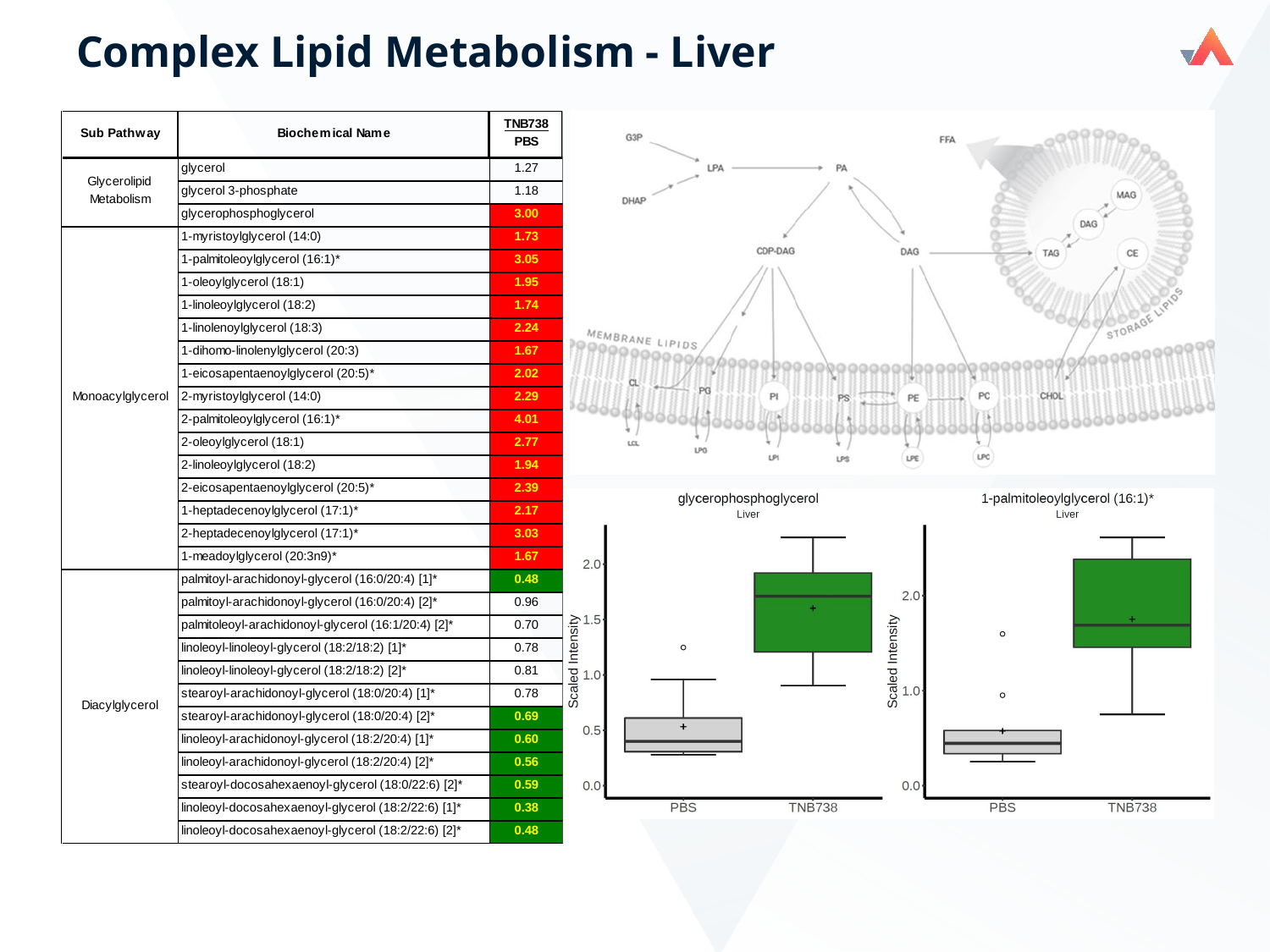

# Complex Lipid Metabolism - Liver

## Slide 27
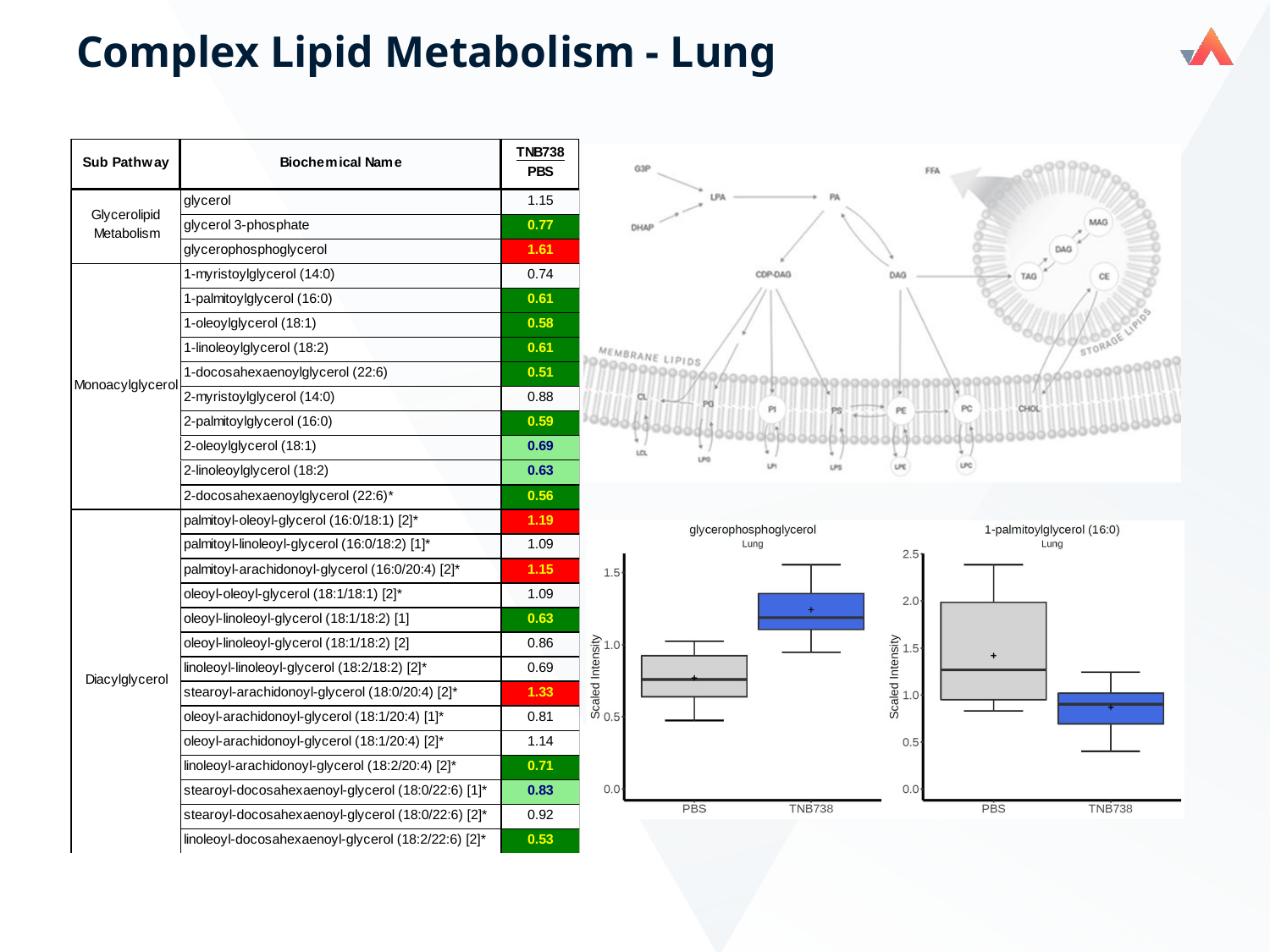

# Complex Lipid Metabolism - Lung

## Slide 28
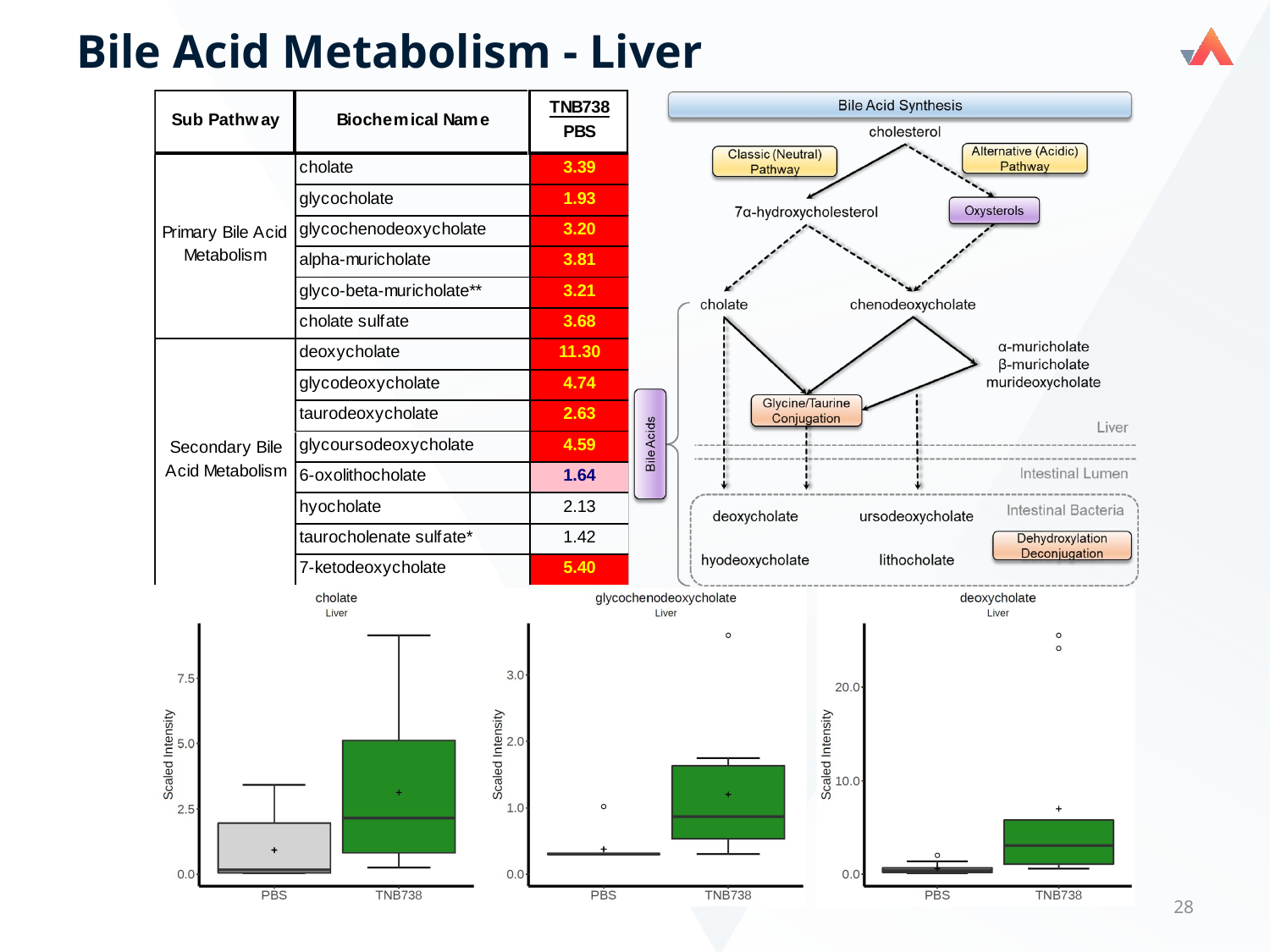

# Bile Acid Metabolism - Liver
28

## Slide 29
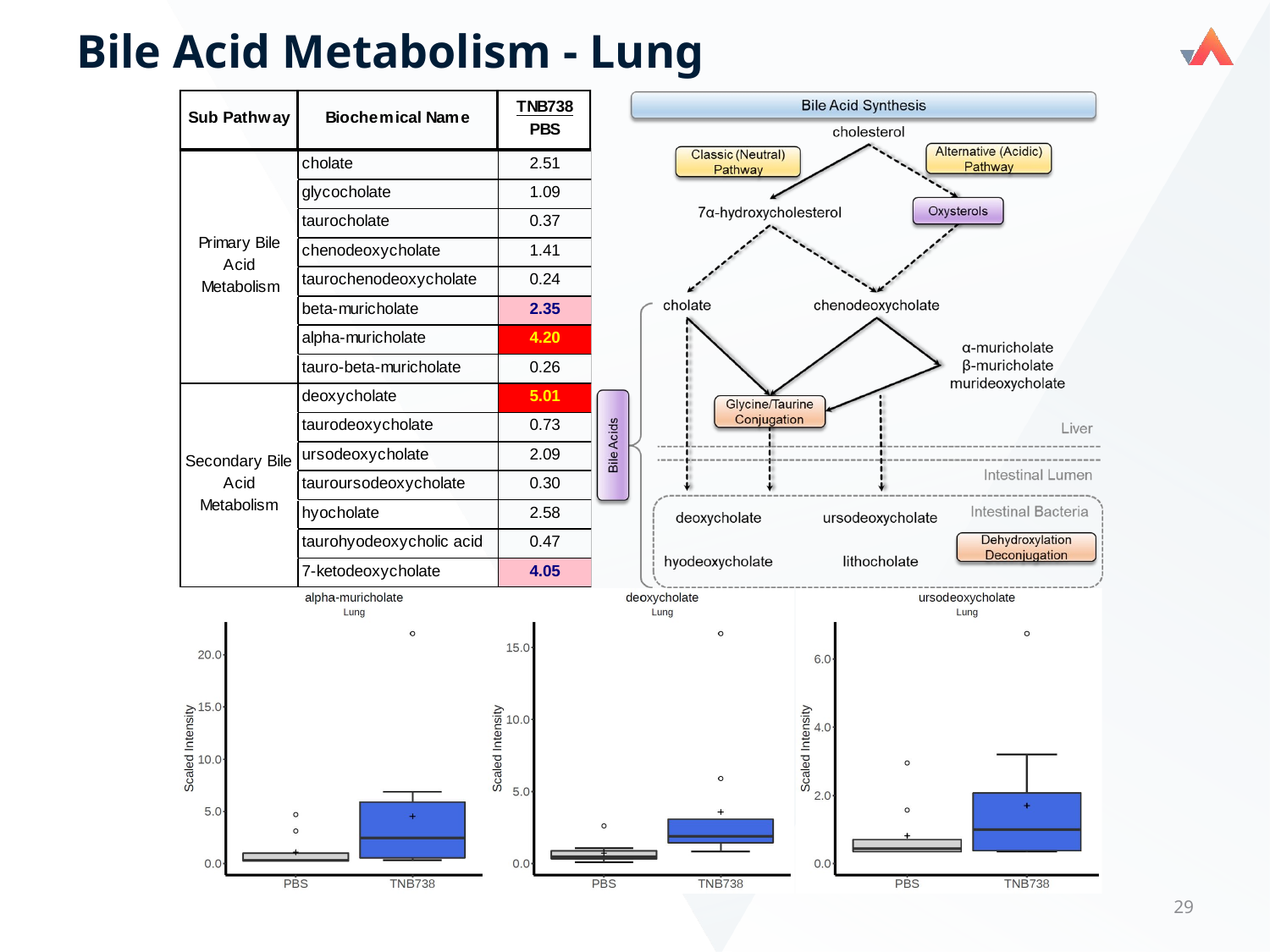

# Bile Acid Metabolism - Lung
29

## Slide 30
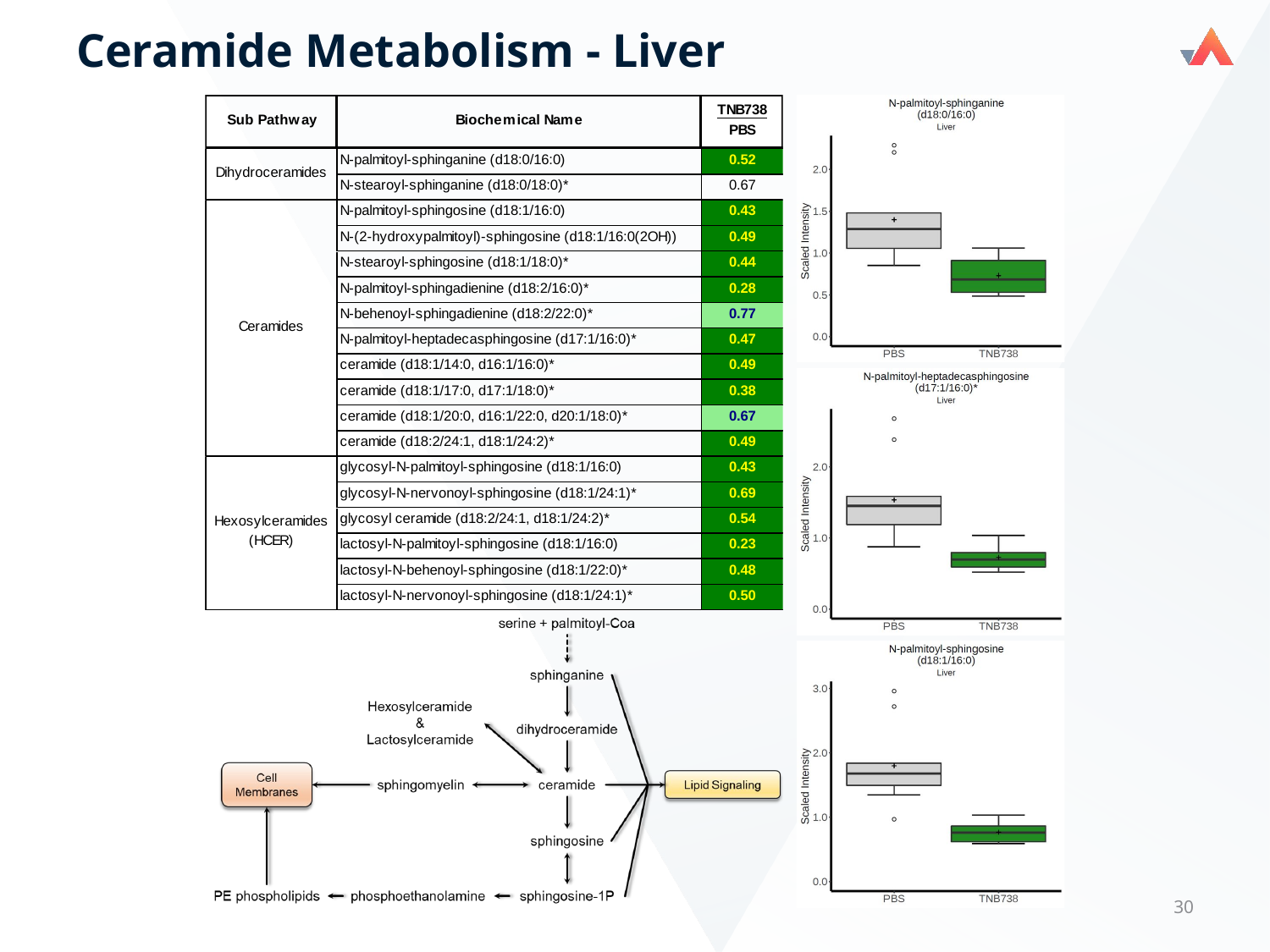

# Ceramide Metabolism - Liver
30

## Slide 31
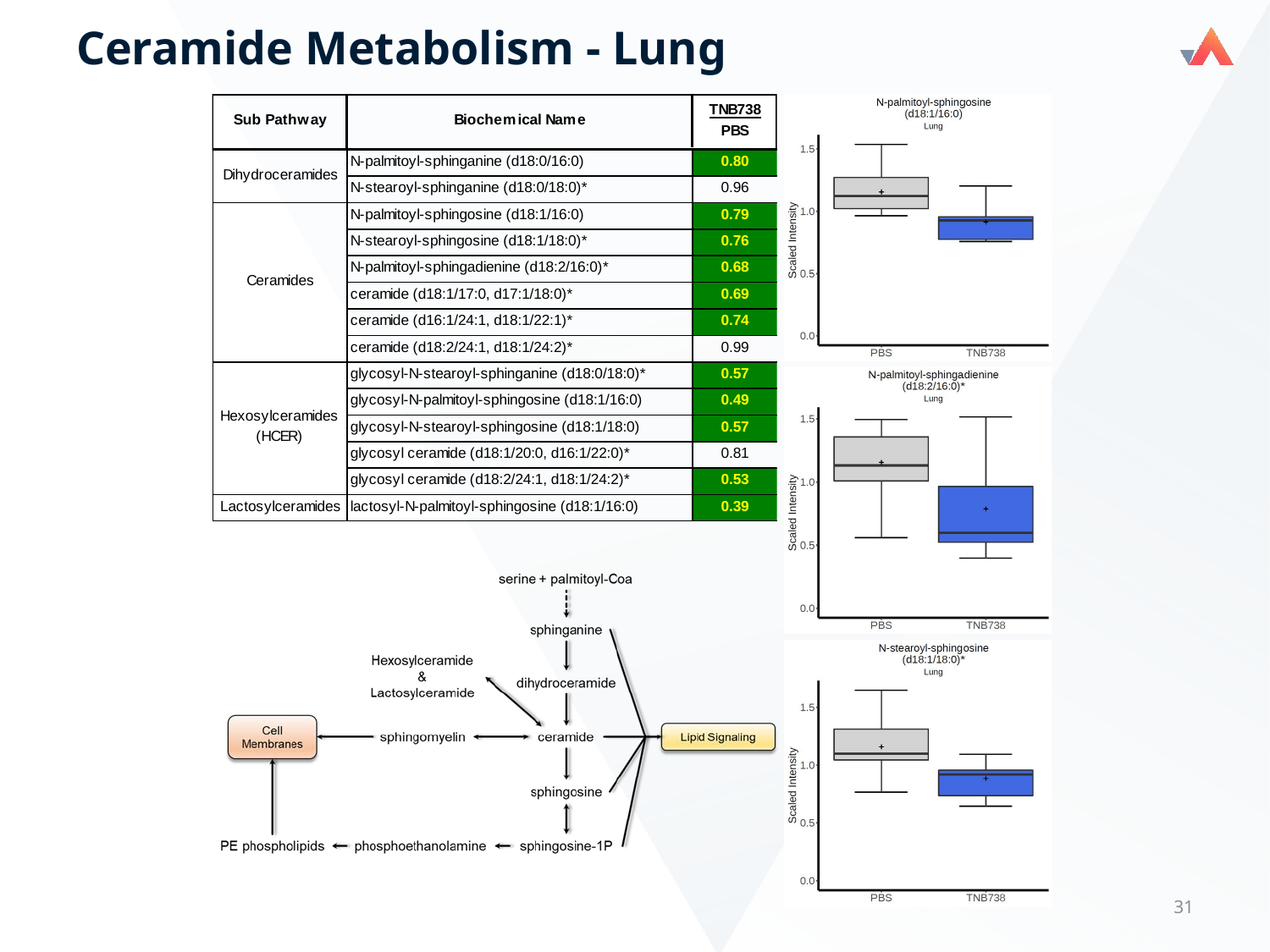

# Ceramide Metabolism - Lung
31

## Slide 32
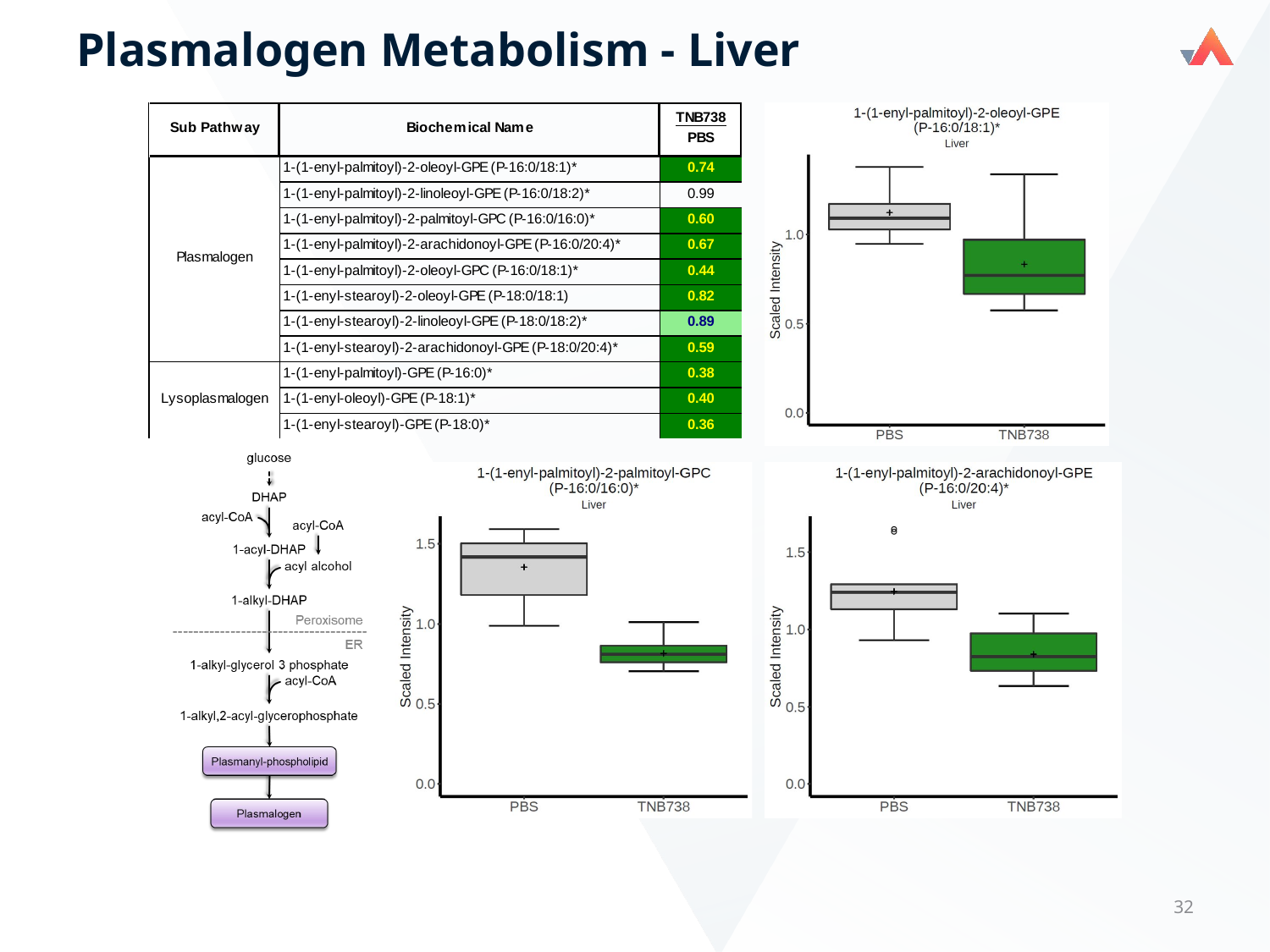

# Plasmalogen Metabolism - Liver
32

## Slide 33
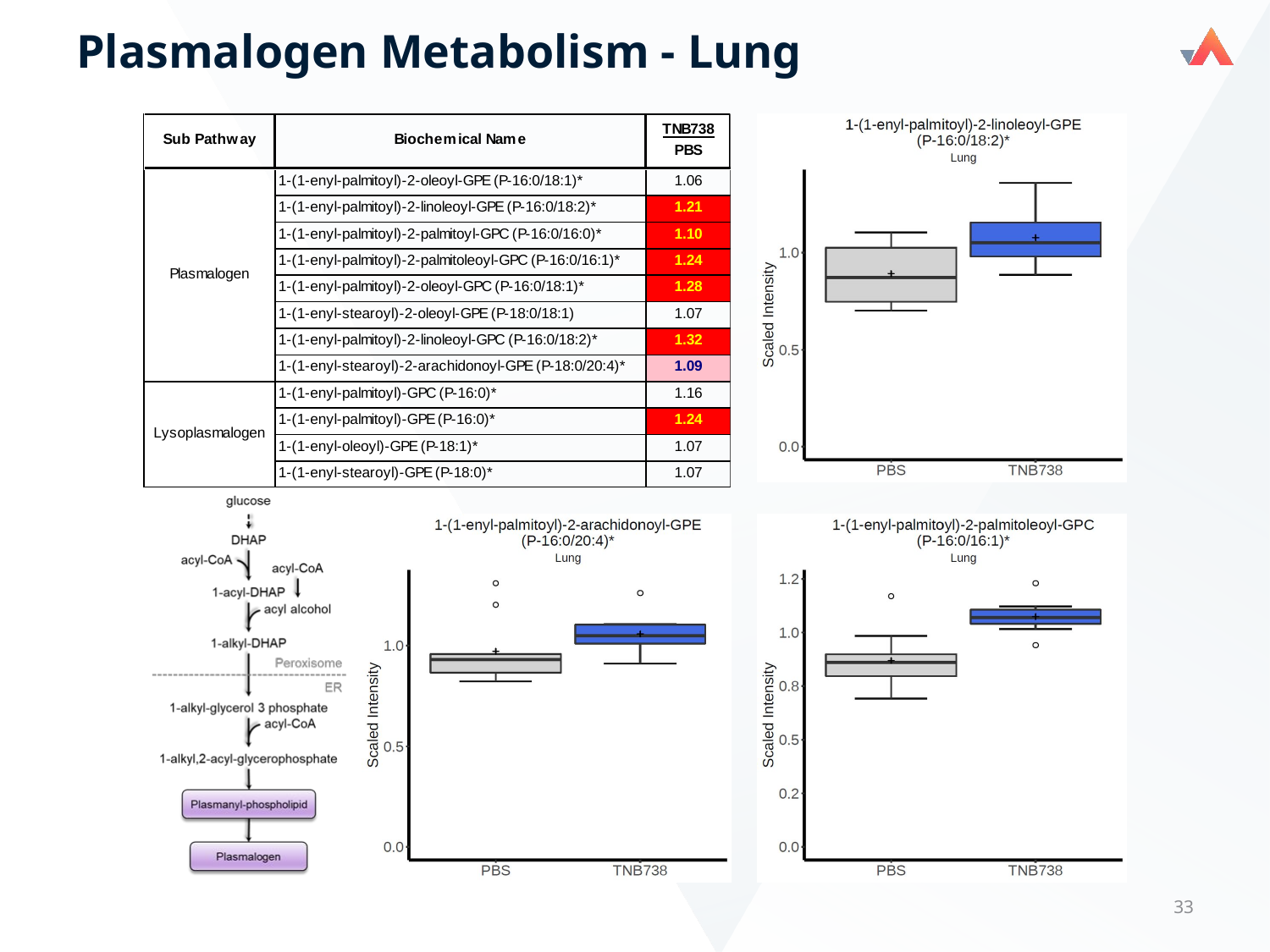

# Plasmalogen Metabolism - Lung
33

## Slide 34
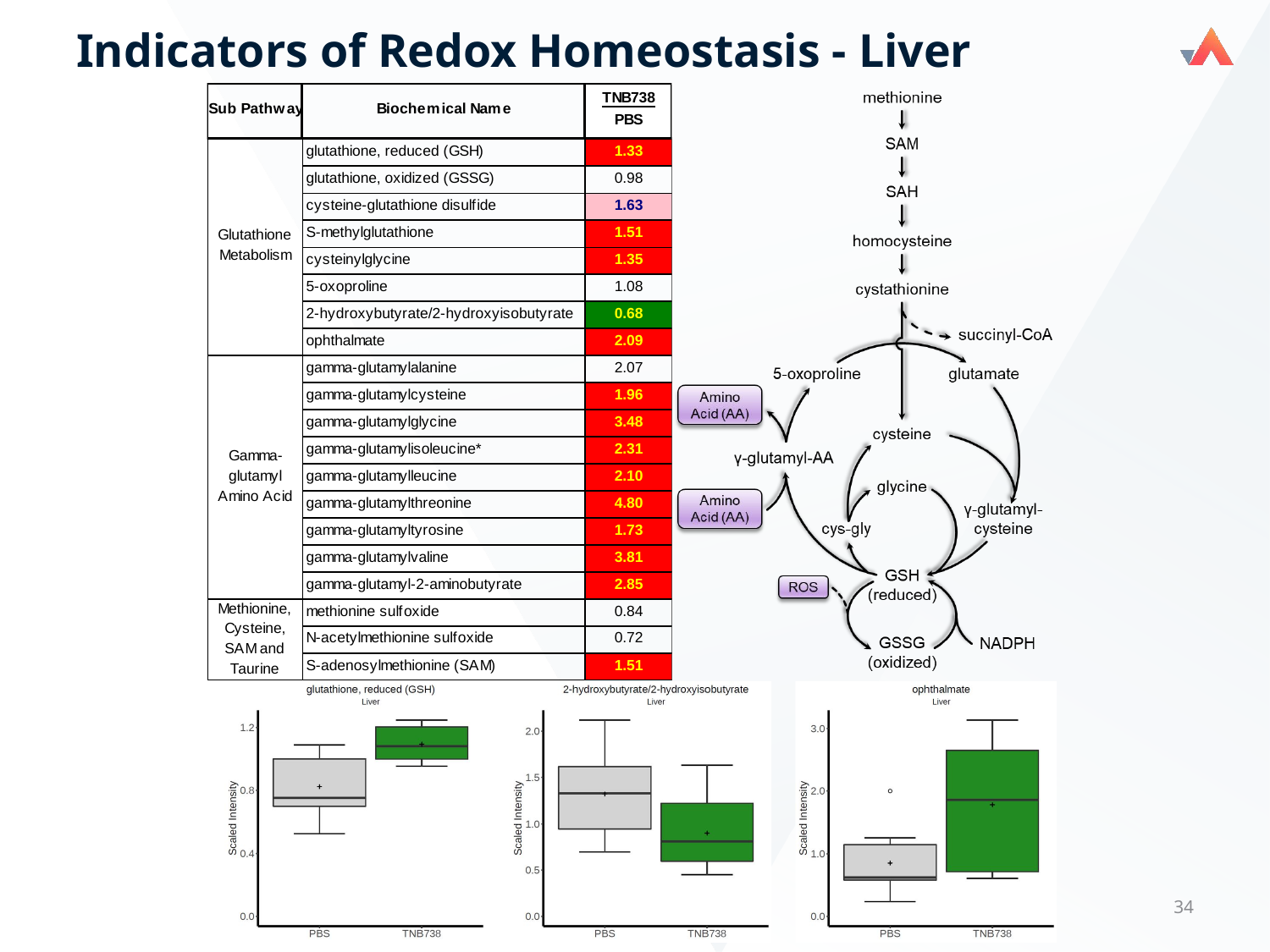

# Indicators of Redox Homeostasis - Liver
34

## Slide 35
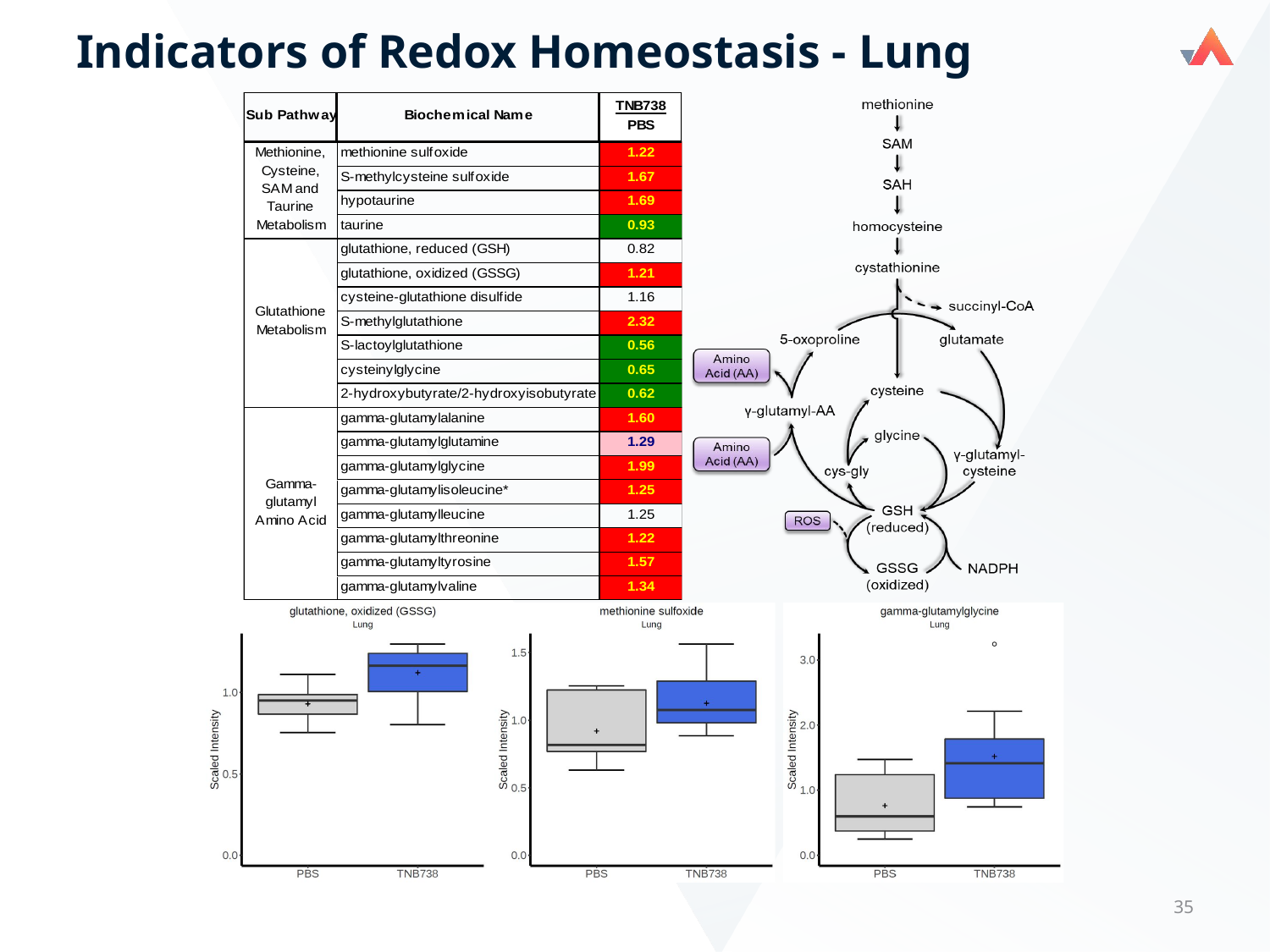

# Indicators of Redox Homeostasis - Lung
35

## Slide 36
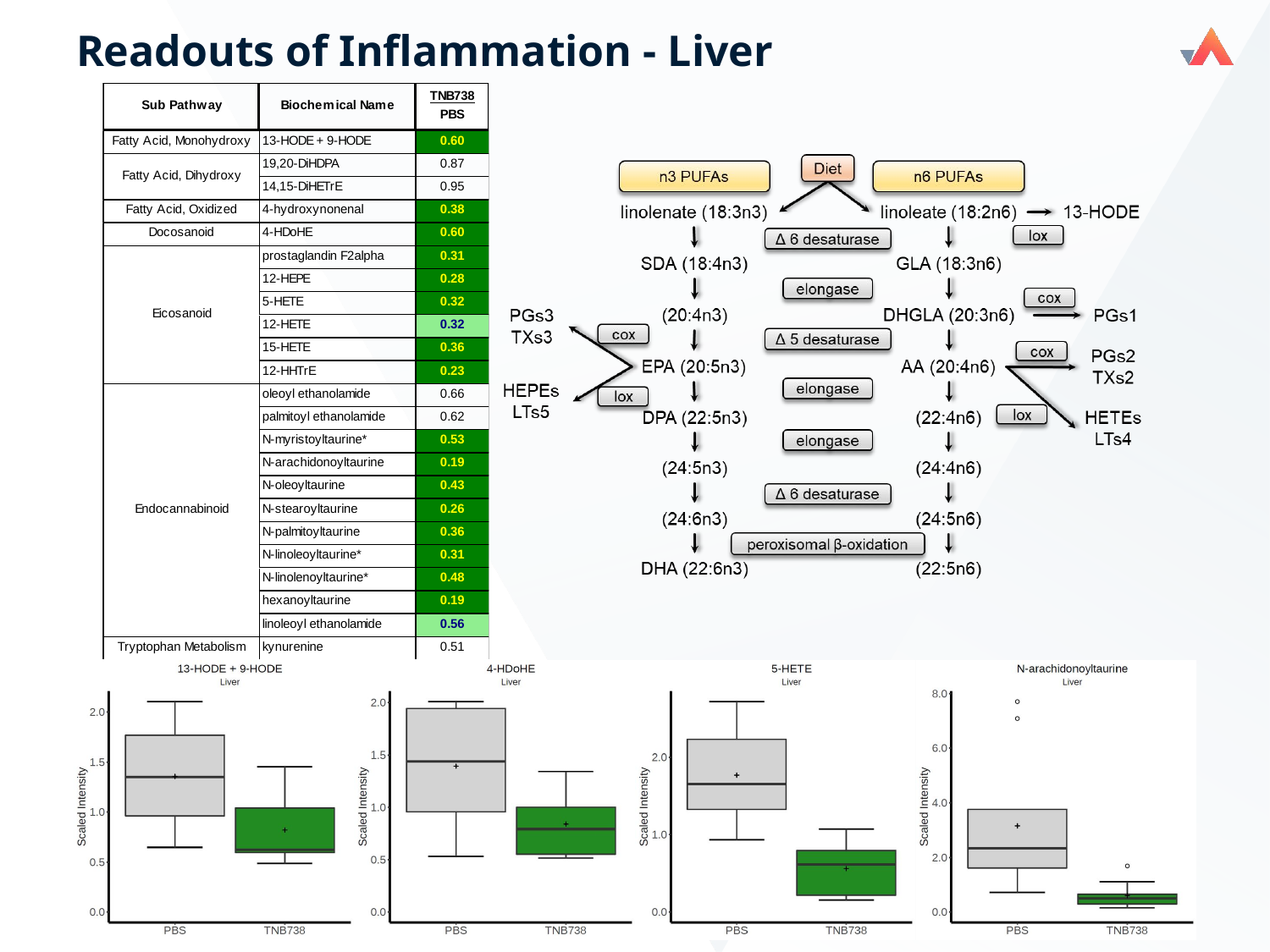

# Readouts of Inflammation - Liver

## Slide 37
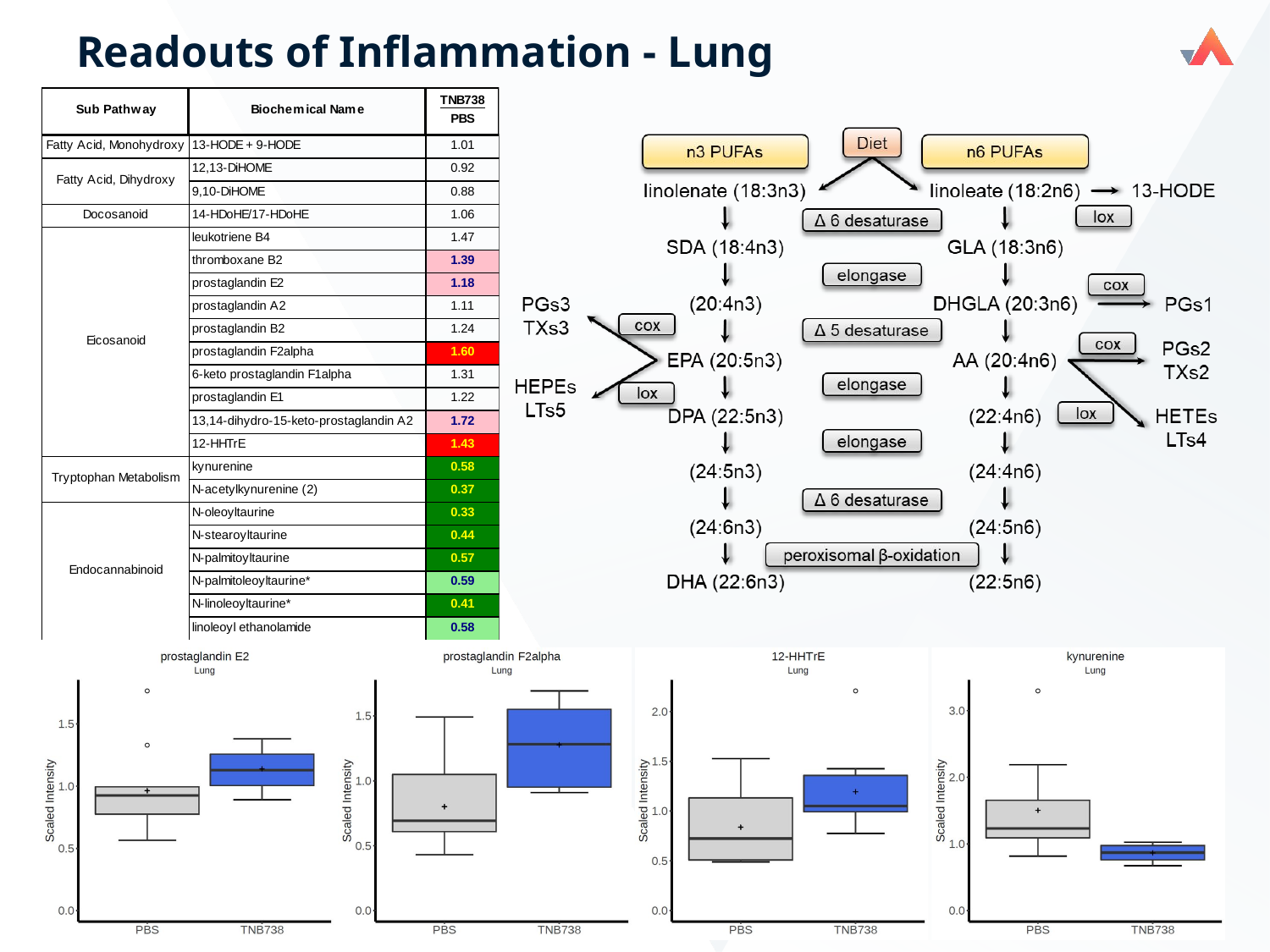

# Readouts of Inflammation - Lung

## Slide 38
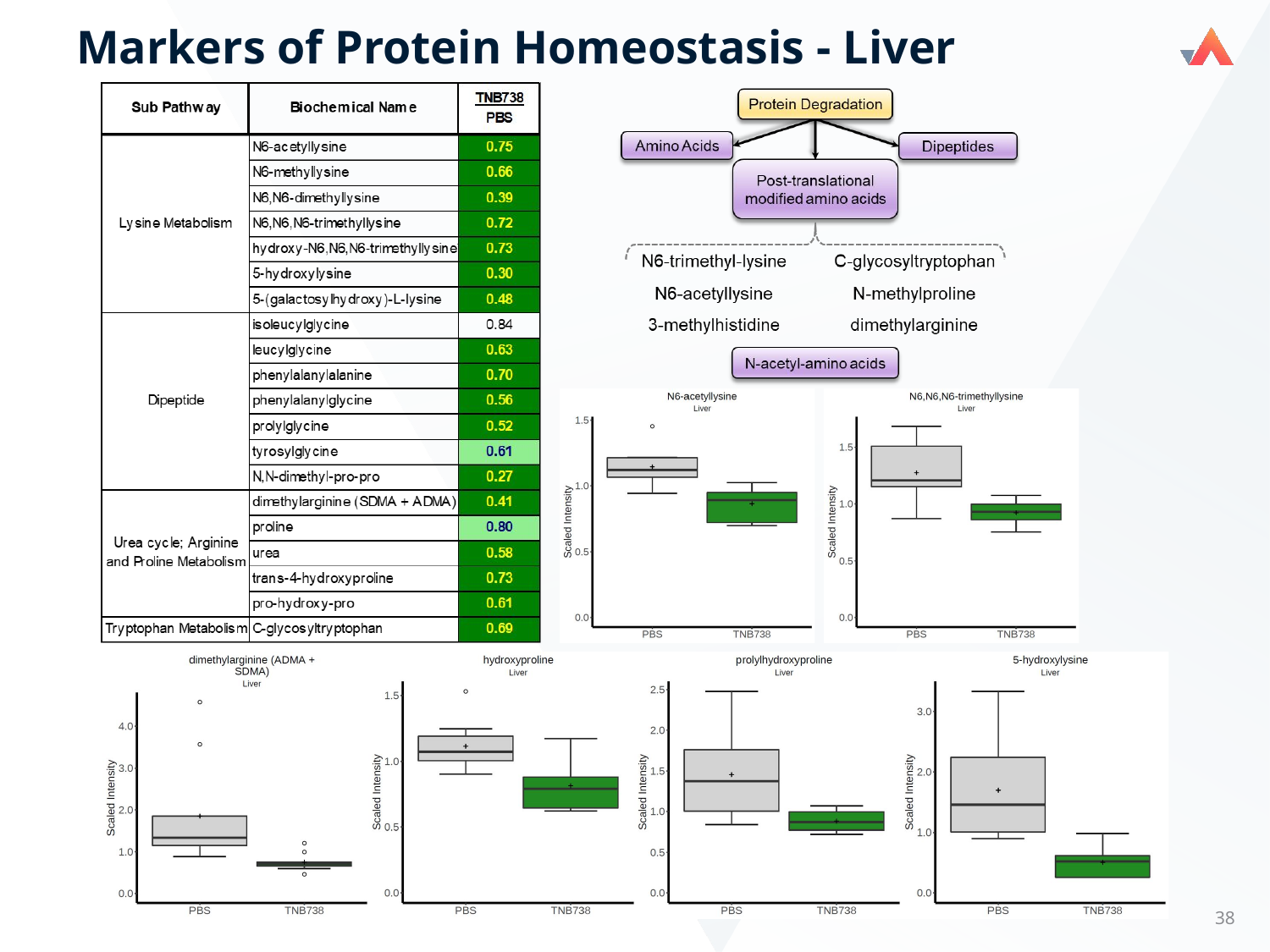

# Markers of Protein Homeostasis - Liver
38

## Slide 39
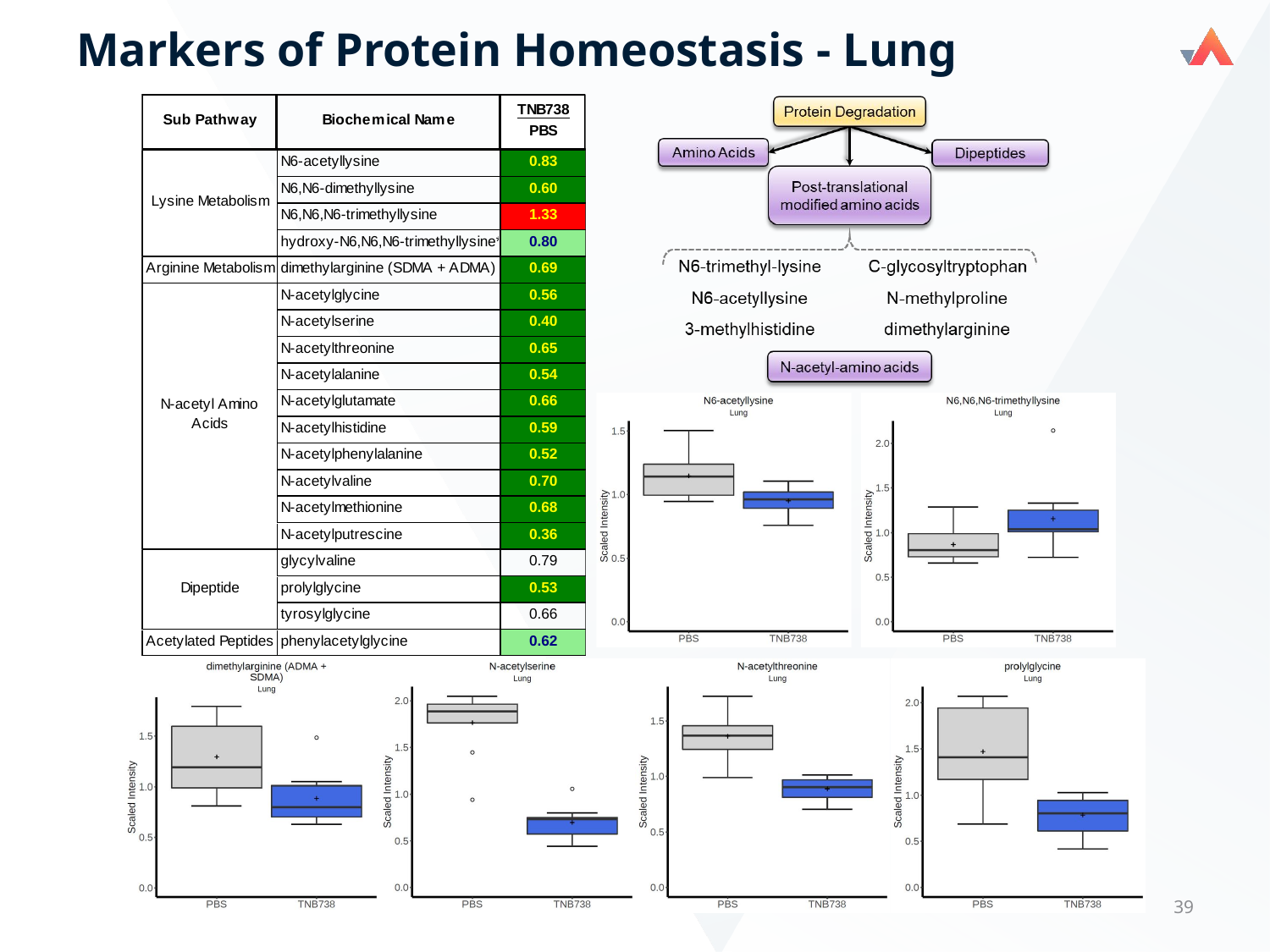

# Markers of Protein Homeostasis - Lung
39

## Slide 40
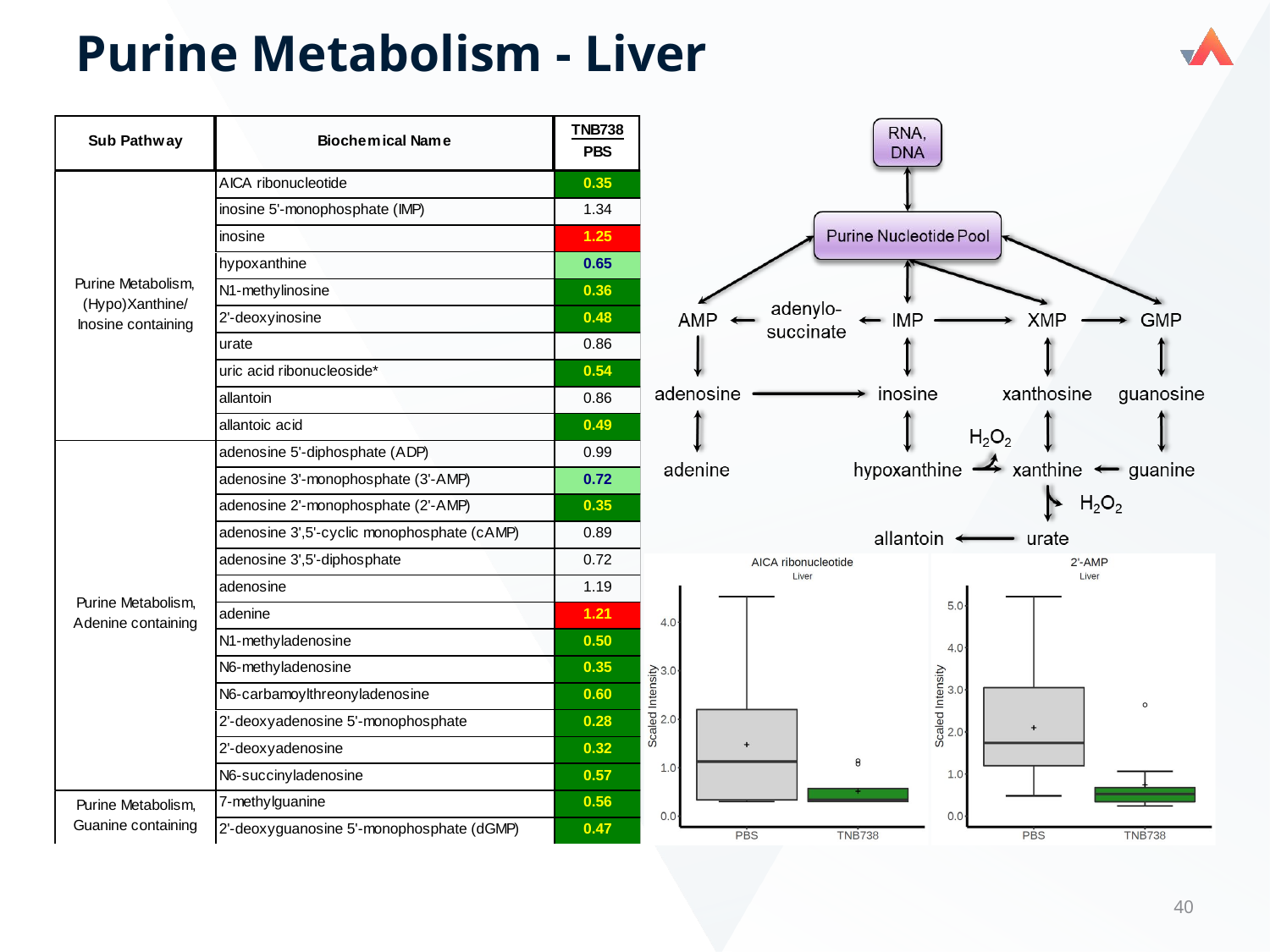

Purine Metabolism - Liver
40

## Slide 41
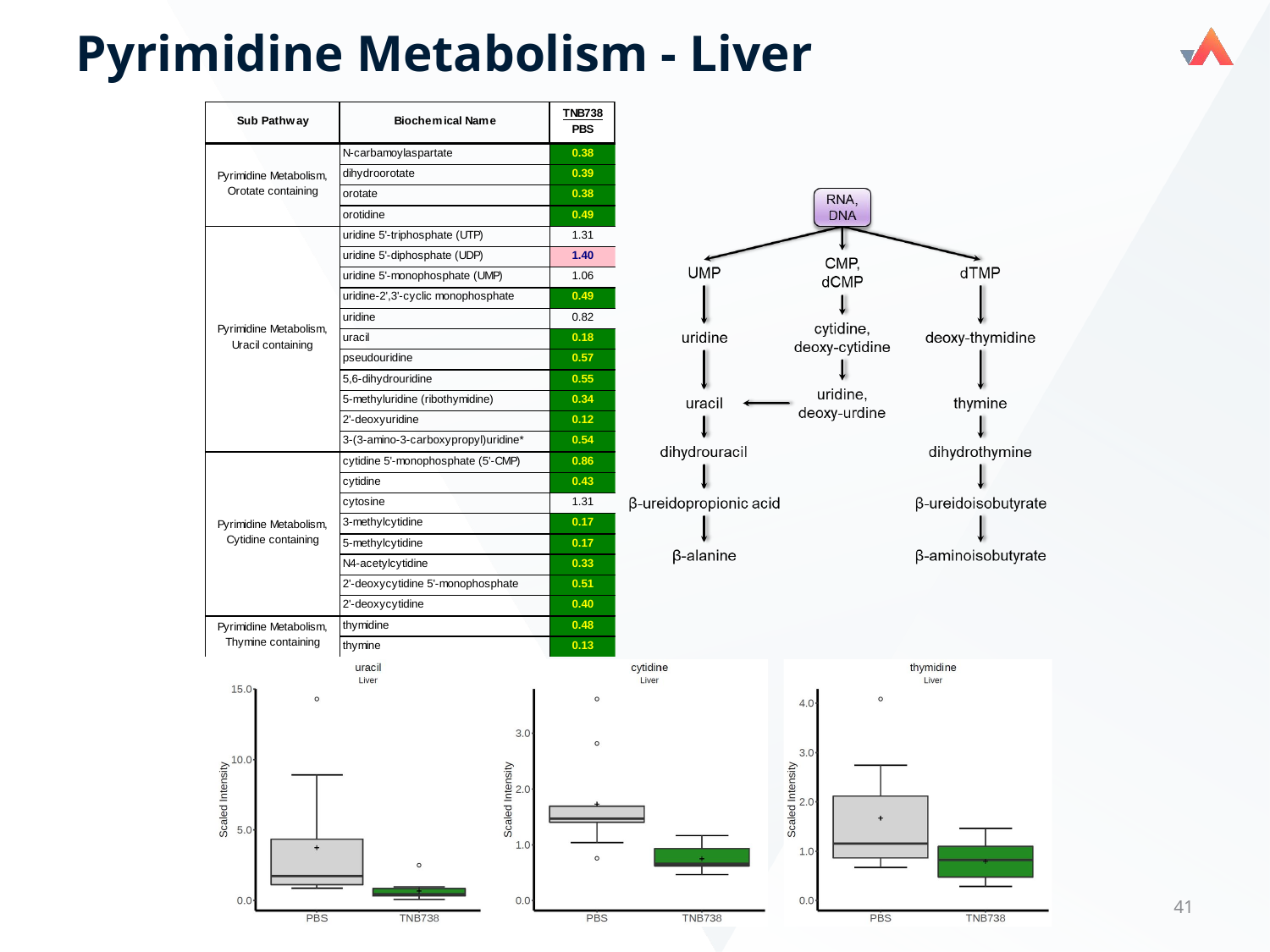

Pyrimidine Metabolism - Liver
41

## Slide 42
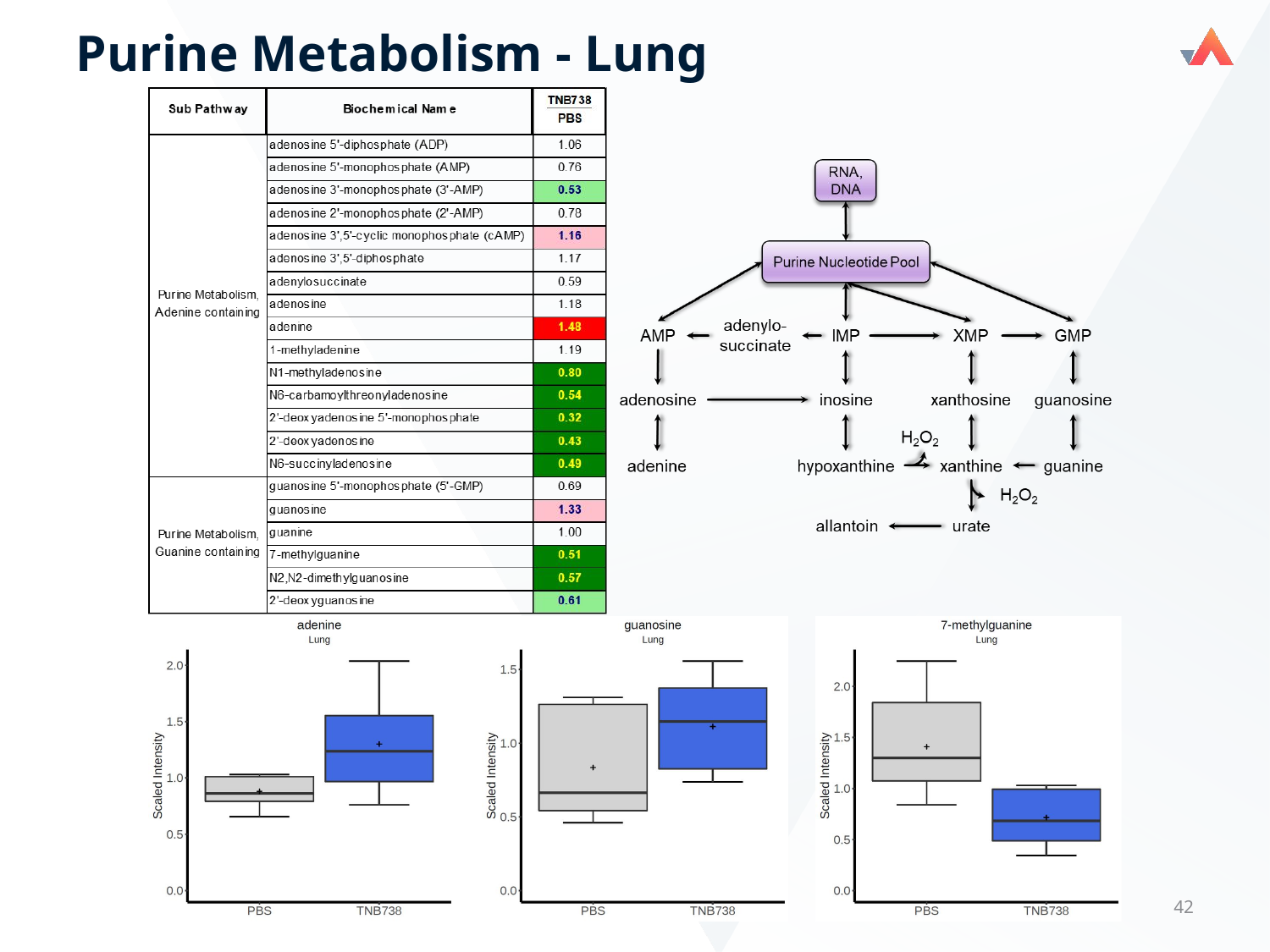

Purine Metabolism - Lung
42

## Slide 43
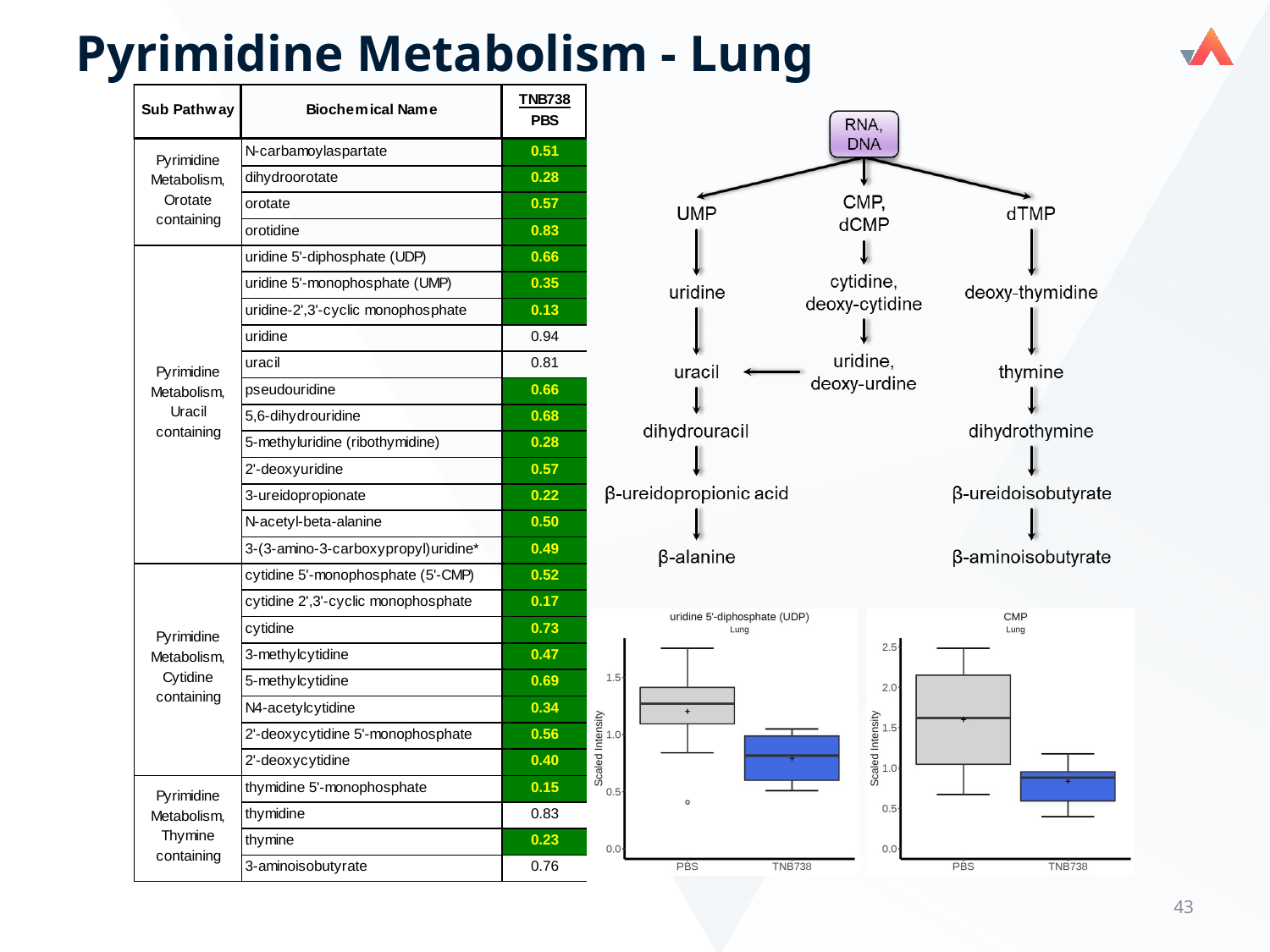

Pyrimidine Metabolism - Lung
43

## Slide 44
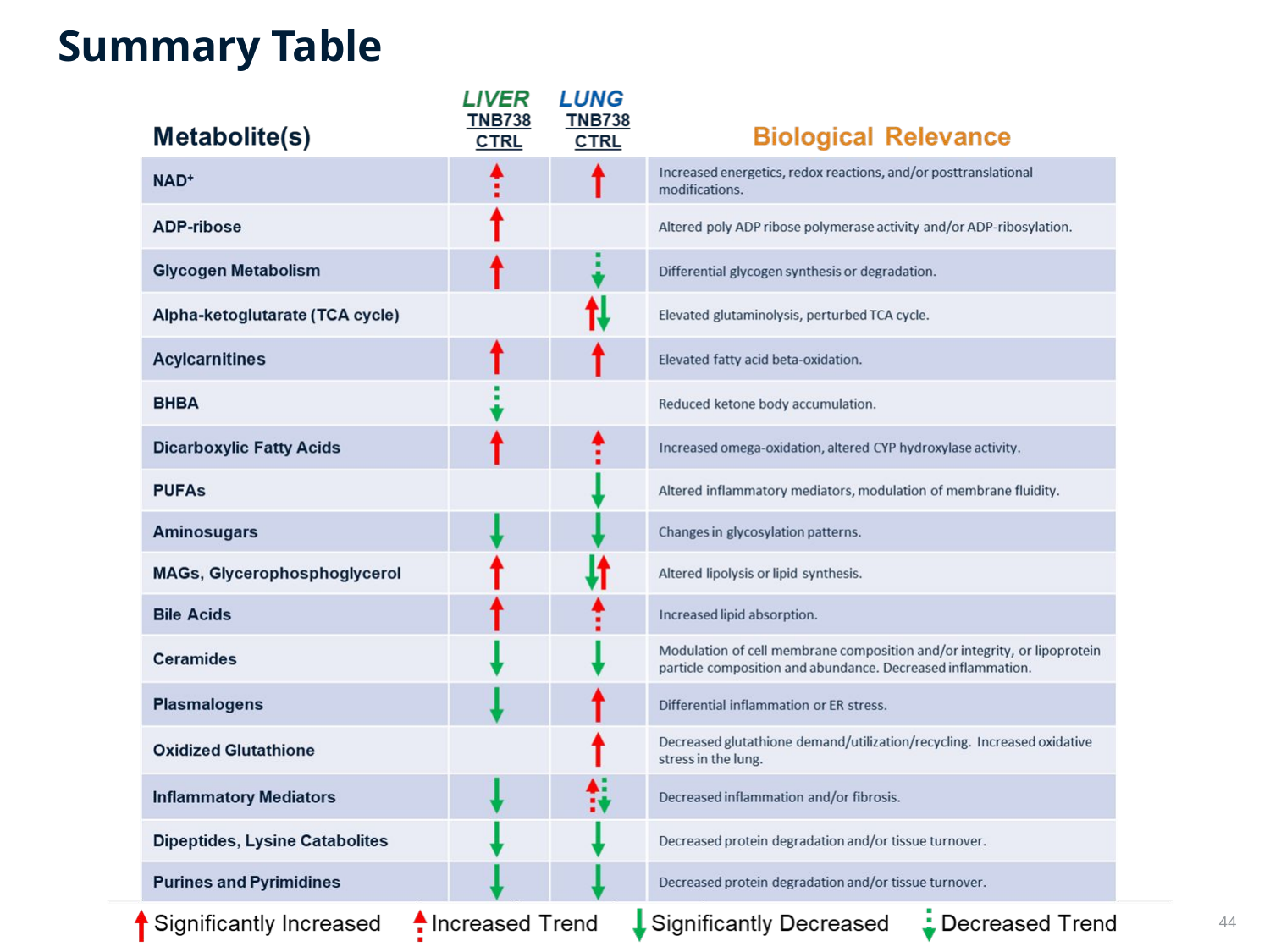

# Summary Table
44

## Slide 45
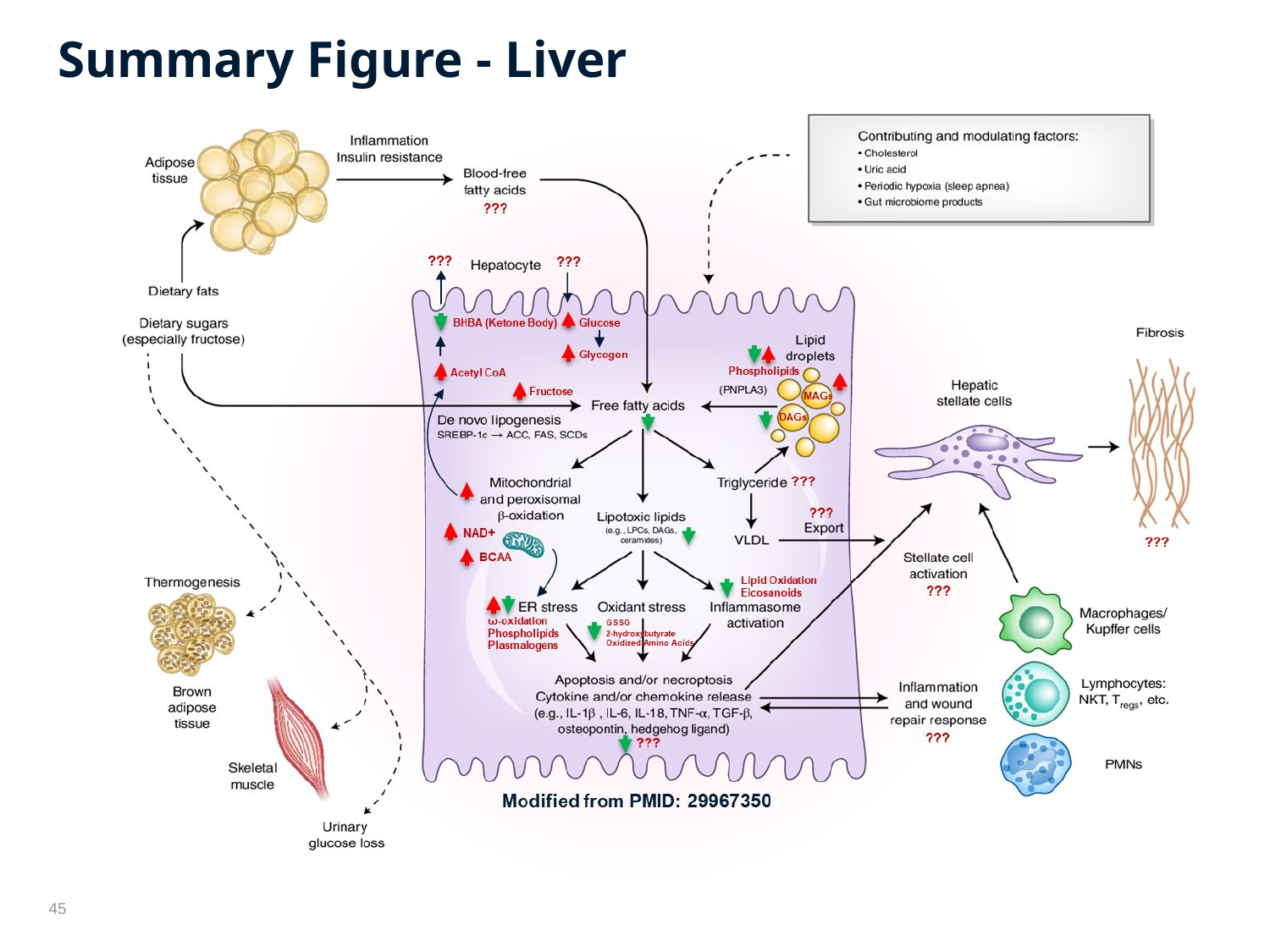

# Summary Figure - Liver
45

## Slide 46
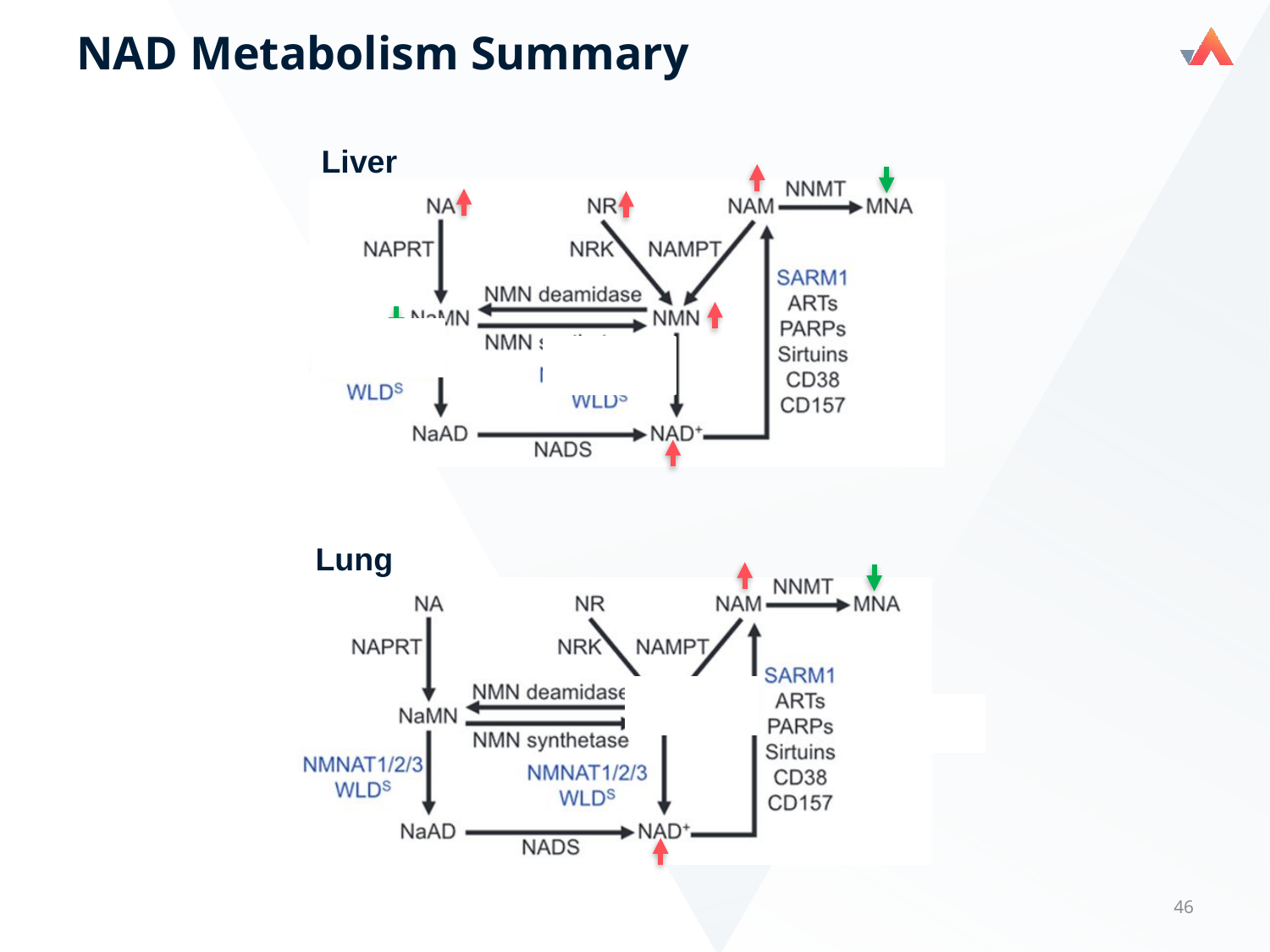

# NAD Metabolism Summary
Liver
Lung
46

## Slide 47
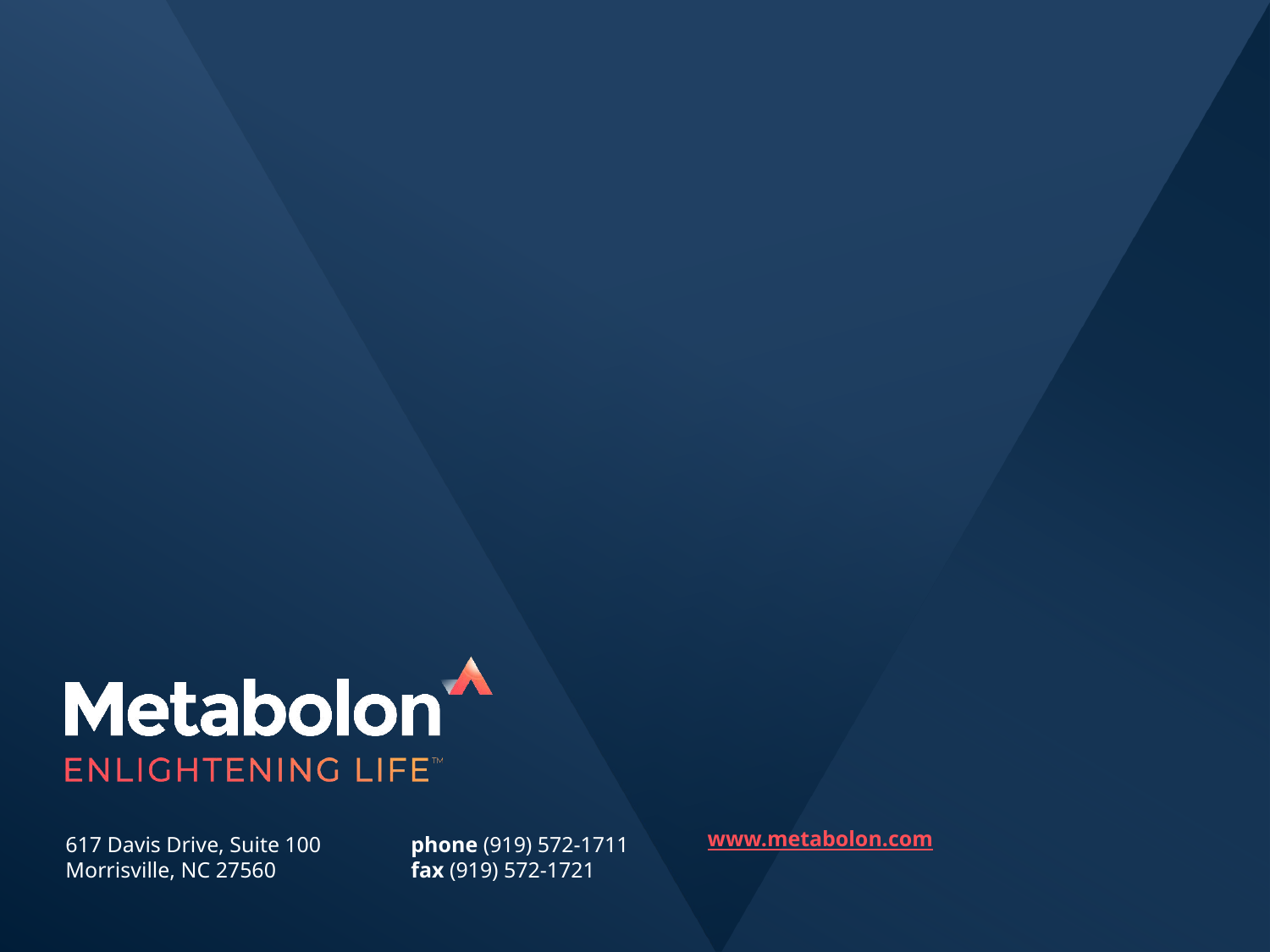

Supplement: Supplementary file 4 [file Supplementaryfile3.pptx]
